# Identification of four novel susceptibility loci for oestrogen receptor negative breast cancer

**DOI:** 10.1038/ncomms11375

**Published:** 2016-04-27

**Authors:** Fergus J. Couch, Karoline B. Kuchenbaecker, Kyriaki Michailidou, Gustavo A. Mendoza-Fandino, Silje Nord, Janna Lilyquist, Curtis Olswold, Emily Hallberg, Simona Agata, Habibul Ahsan, Kristiina Aittomäki, Christine Ambrosone, Irene L. Andrulis, Hoda Anton-Culver, Volker Arndt, Banu K. Arun, Brita Arver, Monica Barile, Rosa B. Barkardottir, Daniel Barrowdale, Lars Beckmann, Matthias W. Beckmann, Javier Benitez, Stephanie V. Blank, Carl Blomqvist, Natalia V. Bogdanova, Stig E. Bojesen, Manjeet K. Bolla, Bernardo Bonanni, Hiltrud Brauch, Hermann Brenner, Barbara Burwinkel, Saundra S. Buys, Trinidad Caldes, Maria A. Caligo, Federico Canzian, Jane Carpenter, Jenny Chang-Claude, Stephen J. Chanock, Wendy K. Chung, Kathleen B. M. Claes, Angela Cox, Simon S. Cross, Julie M. Cunningham, Kamila Czene, Mary B. Daly, Francesca Damiola, Hatef Darabi, Miguel de la Hoya, Peter Devilee, Orland Diez, Yuan C. Ding, Riccardo Dolcetti, Susan M. Domchek, Cecilia M. Dorfling, Isabel dos-Santos-Silva, Martine Dumont, Alison M. Dunning, Diana M. Eccles, Hans Ehrencrona, Arif B. Ekici, Heather Eliassen, Steve Ellis, Peter A. Fasching, Jonine Figueroa, Dieter Flesch-Janys, Asta Försti, Florentia Fostira, William D. Foulkes, Tara Friebel, Eitan Friedman, Debra Frost, Marike Gabrielson, Marilie D. Gammon, Patricia A. Ganz, Susan M. Gapstur, Judy Garber, Mia M. Gaudet, Simon A. Gayther, Anne-Marie Gerdes, Maya Ghoussaini, Graham G. Giles, Gord Glendon, Andrew K. Godwin, Mark S. Goldberg, David E. Goldgar, Anna González-Neira, Mark H. Greene, Jacek Gronwald, Pascal Guénel, Marc Gunter, Lothar Haeberle, Christopher A. Haiman, Ute Hamann, Thomas V. O. Hansen, Steven Hart, Sue Healey, Tuomas Heikkinen, Brian E. Henderson, Josef Herzog, Frans B. L. Hogervorst, Antoinette Hollestelle, Maartje J. Hooning, Robert N. Hoover, John L. Hopper, Keith Humphreys, David J. Hunter, Tomasz Huzarski, Evgeny N. Imyanitov, Claudine Isaacs, Anna Jakubowska, Paul James, Ramunas Janavicius, Uffe Birk Jensen, Esther M. John, Michael Jones, Maria Kabisch, Siddhartha Kar, Beth Y. Karlan, Sofia Khan, Kay-Tee Khaw, Muhammad G. Kibriya, Julia A. Knight, Yon-Dschun Ko, Irene Konstantopoulou, Veli-Matti Kosma, Vessela Kristensen, Ava Kwong, Yael Laitman, Diether Lambrechts, Conxi Lazaro, Eunjung Lee, Loic Le Marchand, Jenny Lester, Annika Lindblom, Noralane Lindor, Sara Lindstrom, Jianjun Liu, Jirong Long, Jan Lubinski, Phuong L. Mai, Enes Makalic, Kathleen E. Malone, Arto Mannermaa, Siranoush Manoukian, Sara Margolin, Frederik Marme, John W. M. Martens, Lesley McGuffog, Alfons Meindl, Austin Miller, Roger L. Milne, Penelope Miron, Marco Montagna, Sylvie Mazoyer, Anna M. Mulligan, Taru A. Muranen, Katherine L. Nathanson, Susan L. Neuhausen, Heli Nevanlinna, Børge G. Nordestgaard, Robert L. Nussbaum, Kenneth Offit, Edith Olah, Olufunmilayo I. Olopade, Janet E. Olson, Ana Osorio, Sue K. Park, Petra H. Peeters, Bernard Peissel, Paolo Peterlongo, Julian Peto, Catherine M. Phelan, Robert Pilarski, Bruce Poppe, Katri Pylkäs, Paolo Radice, Nazneen Rahman, Johanna Rantala, Christine Rappaport, Gad Rennert, Andrea Richardson, Mark Robson, Isabelle Romieu, Anja Rudolph, Emiel J. Rutgers, Maria-Jose Sanchez, Regina M. Santella, Elinor J. Sawyer, Daniel F. Schmidt, Marjanka K. Schmidt, Rita K. Schmutzler, Fredrick Schumacher, Rodney Scott, Leigha Senter, Priyanka Sharma, Jacques Simard, Christian F. Singer, Olga M. Sinilnikova, Penny Soucy, Melissa Southey, Doris Steinemann, Marie Stenmark-Askmalm, Dominique Stoppa-Lyonnet, Anthony Swerdlow, Csilla I. Szabo, Rulla Tamimi, William Tapper, Manuel R. Teixeira, Soo-Hwang Teo, Mary B. Terry, Mads Thomassen, Deborah Thompson, Laima Tihomirova, Amanda E. Toland, Robert A. E. M. Tollenaar, Ian Tomlinson, Thérèse Truong, Helen Tsimiklis, Alex Teulé, Rosario Tumino, Nadine Tung, Clare Turnbull, Giski Ursin, Carolien H. M. van Deurzen, Elizabeth J. van Rensburg, Raymonda Varon-Mateeva, Zhaoming Wang, Shan Wang-Gohrke, Elisabete Weiderpass, Jeffrey N. Weitzel, Alice Whittemore, Hans Wildiers, Robert Winqvist, Xiaohong R. Yang, Drakoulis Yannoukakos, Song Yao, M Pilar Zamora, Wei Zheng, Per Hall, Peter Kraft, Celine Vachon, Susan Slager, Georgia Chenevix-Trench, Paul D. P. Pharoah, Alvaro A. N. Monteiro, Montserrat García-Closas, Douglas F. Easton, Antonis C. Antoniou

**Affiliations:** 1 Department of Laboratory Medicine and Pathology, Mayo Clinic, Rochester, Minnesota 55905, USA; 2 Department of Health Sciences Research, Mayo Clinic, Rochester, Minnesota 55905, USA; 3 Centre for Cancer Genetic Epidemiology, Department of Public Health and Primary Care, University of Cambridge, Cambridge CB1 8RN, UK; 4 Cancer Epidemiology Program, H. Lee Moffitt Cancer Center and Research Institute, Tampa, Florida 33612, USA; 5 Department of Genetics, Institute for Cancer Research, Oslo University Hospital, Radiumhospitalet, N-0310 Oslo, Norway; 6 Immunology and Molecular Oncology Unit, Istituto Oncologico Veneto IOV—IRCCS, 20133 Padua, Italy; 7 Department of Health Studies, The University of Chicago, Chicago, Illinois 60637, USA; 8 Comprehensive Cancer Center, The University of Chicago, Chicago, Illinois 60637, USA; 9 Departments of Medicine and Human Genetics, The University of Chicago, Chicago, Illinois 60637, USA; 10 Department of Clinical Genetics, Helsinki University Central Hospital, 00029 Helsinki, Finland; 11 Department of Cancer Prevention and Control, Roswell Park Cancer Institute, Buffalo, New York 14263, USA; 12 Lunenfeld-Tanenbaum Research Institute of Mount Sinai Hospital, Toronto, Ontario, Canada M5G 1X5; 13 Departments of Molecular Genetics and Laboratory Medicine and Pathobiology, University of Toronto, Ontario, Canada M5B 1W8; 14 Department of Epidemiology, University of California Irvine, Irvine, California, 92697, USA; 15 Division of Clinical Epidemiology and Aging Research, German Cancer Research Center (DKFZ), 69120 Heidelberg, Germany; 16 University of Texas MD Anderson Cancer Center, Houston, Texas 77030, USA; 17 Department of Oncology, Karolinska University Hospital, SE-17176 Stockholm, Sweden; 18 Division of Cancer Prevention and Genetics, Istituto Europeo di Oncologia, 20141 Milan, Italy; 19 Department of Pathology, Landspitali University Hospital and University of Iceland School of Medicine, 101 Reykjavik, Iceland; 20 Institute for Quality and Efficiency in Health Care (IQWiG), 50670 Cologne, Germany; 21 University Breast Center Franconia, Department of Gynecology and Obstetrics, University Hospital Erlangen, Friedrich-Alexander University Erlangen-Nuremberg, Comprehensive Cancer Center Erlangen-EMN, 91054 Erlangen, Germany; 22 Human Genetics Group, Human Cancer Genetics Program, Spanish National Cancer Centre (CNIO), 28029 Madrid, Spain; 23 Genotyping Unit (CeGen), Human Cancer Genetics Program, Spanish National Cancer Centre (CNIO), 28029 Madrid, Spain; 24 Biomedical Network on Rare Diseases (CIBERER), 28029 Madrid, Spain; 25 NYU Women's Cancer Program, New York University School of Medicine, New York, New York 10016, USA; 26 Department of Oncology, University of Helsinki and Helsinki University Central Hospital, FI-00029 Helsinki, Finland; 27 Department of Radiation Oncology, Hannover Medical School, 30625 Hannover, Germany; 28 Copenhagen General Population Study, Herlev Hospital, Copenhagen University Hospital, 2730 Herlev, Denmark; 29 Dr Margarete Fischer-Bosch-Institute of Clinical Pharmacology, 70376 Stuttgart, Germany; 30 University of Tübingen 72074 Tübingen, Germany; 31 Division of Preventive Oncology, German Cancer Research Center (DKFZ) and National Center for Tumor Diseases (NCT), 69120 Heidelberg, Germany; 32 Department of Obstetrics and Gynecology, University of Heidelberg, 69120 Heidelberg, Germany; 33 Department of Medicine, Huntsman Cancer Institute, University of Utah School of Medicine, Salt Lake City Utah 84112, USA; 34 Molecular Oncology Laboratory, Hospital Clinico San Carlos, IdISSC, Madrid 28040, Spain; 35 Section of Genetic Oncology, Department of Laboratory Medicine, University and University Hospital of Pisa, I-56126 Pisa, Italy; 36 Genomic Epidemiology Group, German Cancer Research Center (DKFZ), 69120 Heidelberg, Germany; 37 Australian Breast Cancer Tissue Bank, Westmead Millennium Institute, University of Sydney, Sydney, New South Wales 2145, Australia; 38 Division of Cancer Epidemiology, German Cancer Research Center (DKFZ), 69120 Heidelberg, Germany; 39 Division of Cancer Epidemiology and Genetics, National Cancer Institute, Rockville, Maryland 20850, USA; 40 Departments of Pediatrics and Medicine, Columbia University, New York, New York 10032, USA; 41 Center for Medical Genetics, Ghent University, 9000 Ghent, Belgium; 42 Sheffield Cancer Research Centre, Department of Oncology, University of Sheffield, Sheffield S10 2RX, UK; 43 Academic Unit of Pathology, Department of Neuroscience, University of Sheffield, Sheffield S10 2HQ, UK; 44 Department of Medical Epidemiology and Biostatistics, Karolinska Institutet, SE-17177 Stockholm, Sweden; 45 Department of Clinical Genetics, Fox Chase Cancer Center, Philadelphia, Pennsylvania 19111, USA; 46 INSERM U1052, CNRS UMR5286, Université Lyon, Centre de Recherche en Cancérologie de Lyon, 69373 Lyon, France; 47 Department of Human Genetics and Department of Pathology, Leiden University Medical Center, Leiden 2333 ZC, The Netherlands; 48 Oncogenetics Group, University Hospital Vall d'Hebron, Vall d'Hebron Institute of Oncology (VHIO) and Universitat Autònoma de Barcelona, 08035 Barcelona, Spain; 49 Department of Population Sciences, Beckman Research Institute of City of Hope, Duarte, California 91010, USA; 50 Cancer Bioimmunotherapy Unit, CRO Aviano National Cancer Institute, 33081 Aviano , Italy; 51 Abramson Cancer Center, Perelman School of Medicine, University of Pennsylvania, Pennsylvania 19104, USA; 52 Department of Genetics, University of Pretoria, Pretoria 0007, South Africa; 53 Department of Non-Communicable Disease Epidemiology, London School of Hygiene and Tropical Medicine, London, WC1E 7HT, UK; 54 Cancer Genomics Laboratory, Centre Hospitalier Universitaire de Québec and Laval University, Quebec City, Quebec, Canada G1V 4G2; 55 Centre for Cancer Genetic Epidemiology, Department of Oncology, University of Cambridge, Cambridge CB1 8RN, UK; 56 Faculty of Medicine, University of Southampton, University Hospital Southampton, Southampton, Hampshire SO16 6YD, UK; 57 Department of Immunology, Genetics and Pathology, Uppsala University, Uppsala SE-751 85, Sweden; 58 Department of Clinical Genetics, Lund University Hospital, SE-22185 Lund, Sweden; 59 Institute of Human Genetics, University Hospital Erlangen, Friedrich-Alexander University Erlangen-Nuremberg, 91054 Erlangen, Germany; 60 Comprehensive Cancer Center -EMN, 91054 Erlangen, Germany; 61 Channing Division of Network Medicine, Brigham and Women's Hospital and Harvard Medical School, Boston, Massachusetts 02115, USA; 62 Department of Epidemiology, Harvard School of Public Health, Boston, Massachusetts 02115, USA; 63 Department of Cancer Epidemiology/Clinical Cancer Registry and Institute for Medical Biometrics and Epidemiology, University Clinic Hamburg-Eppendorf, 20246 Hamburg, Germany; 64 Division of Molecular Genetic Epidemiology, German Cancer Research Center (DKFZ), 69120 Heidelberg, Germany; 65 Center for Primary Health Care Research, Lund University, SE-221 00 Malmö, Sweden; 66 Molecular Diagnostics Laboratory, INRASTES, National Centre for Scientific Research ‘Demokritos', Aghia Paraskevi Attikis, 15310 Athens, Greece; 67 Program in Cancer Genetics, McGill University, Montreal, Quebec, Canada H3A 0G4; 68 University of, Philadelphia, Pennsylvania 19104, USA; 69 Susanne Levy Gertner Oncogenetics Unit, Sheba Medical Center, Tel-Hashomer 52621, Israel; 70 Department of Epidemiology, University of, Chapel Hill, North Carolina 27599-7400, USA; 71 UCLA Schools of Medicine and Public Health, Division of Cancer Prevention and Control Research, Jonsson Comprehensive Cancer Center, Los Angeles, California 90095-6900, USA; 72 Epidemiology Research Program, American Cancer Society, Atlanta, Georgia 30303, USA; 73 Cancer Risk and Prevention Clinic, Dana Farber Cancer Institute, Boston, Massachusetts 02215, USA; 74 Department of Biomedical Sciences, Cedars Sinai Medical Center, Los Angeles, California 90048, USA; 75 Department of Clinical Genetics, Rigshospitalet, Copenhagen University Hospital, DK-2100 Copenhagen, Denmark; 76 Cancer Epidemiology Centre, Cancer Council Victoria, Melbourne, Victoria 3010, Australia; 77 Department of Pathology and Laboratory Medicine, University of Kansas Medical Center, Kansas City, Kansas, 66205, USA; 78 Department of Medicine, McGill University, Montreal, Quebec, Canada H3G 2M1; 79 Division of Clinical Epidemiology, McGill University Health Centre, Royal Victoria Hospital, Montreal, Quebec, Canada H4A 3J1; 80 Department of Dermatology, Huntsman Cancer Institute, University of Utah School of Medicine, Salt Lake City, Utah 84132, USA; 81 Human Genotyping-CEGEN Unit, Human Cancer Genetics Program, Spanish National Cancer Research Centre (CNIO), 28029 Madrid, Spain; 82 Clinical Genetics Branch, Division of Cancer Epidemiology and Genetics, National Cancer Institute, National Institutes of Health, Rockville, Maryland 20850-9772, USA; 83 Department of Genetics and Pathology, Pomeranian Medical University, Szczecin, Poland; 84 Inserm (National Institute of Health and Medical Research), CESP (Center for Research in Epidemiology and Population Health), U1018, Environmental Epidemiology of Cancer, 70-115 Villejuif, France; 85 Department of of Epidemiology and Biostatistics, School of Public Health, Imperial College London, London, SW7 2AZ, UK; 86 Department of Preventive Medicine, Keck School of Medicine, University of Southern California Norris Comprehensive Cancer Center, Los Angeles, California 90033, USA; 87 Molecular Genetics of Breast Cancer, German Cancer Research Center (DKFZ), 69120 Heidelberg, Germany; 88 Center for Genomic Medicine, Rigshospitalet, Copenhagen University Hospital, DK-2100 Copenhagen, Denmark; 89 Department of Genetics, QIMR Berghofer Medical Research Institute, Brisbane, Queensland 4029, Australia; 90 Helsinki University Central Hospital, FI-00029 Helsinki, Finland; 91 Clinical Cancer Genetics, for the City of Hope Clinical Cancer Genetics Community Research Network, Duarte, California 91010, USA; 92 Family Cancer Clinic, Netherlands Cancer Institute, Amsterdam 1000 BE, The Netherlands; 93 Department of Medical Oncology, Erasmus MC Cancer Institute, Rotterdam 3008 AE, The Netherlands; 94 Department of Medical Oncology, Family Cancer Clinic, Erasmus University Medical Center, Rotterdam 3008 AE, The Netherlands; 95 Centre for Epidemiology and Biostatistics, Melbourne School of Population and Global Health, The University of Melbourne, Melbourne, Victoria 3010, Australia; 96 Program in Molecular and Genetic Epidemiology, Harvard School of Public Health, Boston, Massachusetts 02115, USA; 97 N.N. Petrov Institute of Oncology, 197758 St Petersburg, Russia; 98 Lombardi Comprehensive Cancer Center, Georgetown University, Washington, DC 20007, USA; 99 Familial Cancer Centre, Peter MacCallum Cancer Centre, Melbourne, Victoria 8006, Australia; 100 Department of Oncology, The University of Melbourne, Melbourne, Victoria 8006, Australia; 101 State Research Institute Centre for Innovative Medicine, LT-08661 Vilnius, Lithuania; 102 Department of Clinical Genetics, Aarhus University Hospital, 8200 Aarhus N, Denmark; 103 Department of Epidemiology, Cancer Prevention Institute of California, Fremont, California 94538, USA; 104 Division of Genetics and Epidemiology, Institute of Cancer Research, Sutton SM2 5NG, UK; 105 Women's Cancer Program at the Samuel Oschin Comprehensive Cancer Institute, Cedars-Sinai Medical Center, Los Angeles, California, 90048, USA; 106 Department of Obstetrics and Gynecology, University of Helsinki and Helsinki University Central Hospital, FI-00029 Helsinki, Finland; 107 Department of Public Health and Primary Care, University of Cambridge, Strangeways Research Laboratory, Cambridge CB1 8RN, UK; 108 Prosserman Centre for Health Research, Lunenfeld-Tanenbaum Research Institute of Mount Sinai Hospital, Toronto, Ontario, Canada M5G 1X5; 109 Department of Internal Medicine, Evangelische Kliniken Bonn gGmbH, Johanniter Krankenhaus, 53113 Bonn, Germany; 110 School of Medicine, Institute of Clinical Medicine, Pathology and Forensic Medicine, University of Eastern Finland, FI-70211 Kuopio, Finland; 111 The Hong Kong Hereditary Breast Cancer Family Registry, Cancer Genetics Center, Hong Kong Sanatorium and Hospital, Hong Kong; 112 Department of Surgery, The University of Hong Kong, Hong Kong, China; 113 Vesalius Research Center, VIB, 3000 Leuven, Belgium; 114 Molecular Diagnostic Unit, Hereditary Cancer Program, IDIBELL-Catalan Institute of Oncology, 08908 Barcelona, Spain; 115 Department of Preventive Medicine, University of Southern California, Los Angeles, California 90032, USA; 116 Cancer Epidemiology Program, University of Cancer Center, Honolulu, Hawaii 96813, USA; 117 Department of Molecular Medicine and Surgery, Karolinska Institutet, SE-17177 Stockholm, Sweden; 118 Health Sciences Research, Mayo Clinic, Scotsdale, Arizona 85259, USA; 119 Program in Genetic Epidemiology and Statistical Genetics, Harvard School of Public Health, Boston, Massachusetts 02115, USA; 120 Human Genetics Division, Genome Institute of Singapore, Singapore 138672, Singapore; 121 Division of Epidemiology, Department of Medicine, Vanderbilt Epidemiology Center and Vanderbilt-Ingram Cancer Center, Vanderbilt University School of Medicine, Nashville, Tennessee 37203, USA; 122 Division of Public Health Sciences, Fred Hutchinson Cancer Research Center, Seattle, Washington 98109, USA; 123 Department of Epidemiology, School of Public Health and Community Medicine, University of Washington, Seattle, Washington 98195, USA; 124 Unit of Medical Genetics, Department of Preventive and Predictive Medicine, Fondazione IRCCS Istituto Nazionale Tumori (INT), 20133 Milan, Italy; 125 Department of Gynaecology and Obstetrics, Technical University of Munich, 81675 Munich, Germany; 126 NRG Oncology Statistics and Data Management Center, Roswell Park Cancer Institute, Buffalo, New York 14263, USA; 127 Department of Genomics and Genome Sciences, Case Western Reserve University Medical School, Cleveland, Ohio 44106, USA; 128 Laboratory Medicine Program, University Health Network, Toronto, Ontario, Canada M5B 1W8; 129 Department of Laboratory Medicine and Pathobiology, University of Toronto, Toronto, Ontario, Canada M5B 1W8; 130 Invitae Corporation, San Francisco, California 94107, USA; 131 Department of Medicine, Memorial Sloan-Kettering Cancer Center, New York, New York 10065, USA; 132 Department of Molecular Genetics, National Institute of Oncology, H-1122 Budapest, Hungary; 133 Center for Clinical Cancer Genetics and Global Health, University of Chicago Medical Center, Chicago, Illinois 60637, USA; 134 Department of Preventive Medicine and Biomedical Science, Seoul National University College of Medicine and Cancer Research Institute, Seoul National University, 110-799 Seoul, Republic of Korea; 135 Department of Epidemiology, Julius Center for Health Sciences and Primary Care, University Medical Center, Utrecht 3508 GA, The Netherlands; 136 MRC-PHE Centre for Environment and Health, Department of Epidemiology and Biostatistics, School of Public Health, Imperial College London, London SW7 2AZ, UK; 137 IFOM, Fondazione Istituto FIRC di Oncologia Molecolare, 20133 Milan, Italy; 138 Divison of Human Genetics, Department of Internal Medicine, The Comprehensive Cancer Center, The Ohio State University, Columbus, Ohio 43210, USA; 139 Laboratory of Cancer Genetics and Tumor Biology, Department of Clinical Chemistry and Biocenter Oulu, University of Oulu, NordLab Oulu/Oulu University Hospital, FI-90220 Oulu, Finland; 140 Unit of Molecular Bases of Genetic Risk and Genetic Testing, Department of Preventive and Predictive Medicine, Fondazione IRCCS Istituto Nazionale Tumori (INT), 20133 Milan, Italy; 141 Section of Cancer Genetics, Institute of Cancer Research, Sutton SM2 5NG, UK; 142 Department of Clinical Genetics, Karolinska University Hospital, SE-17176 Stockholm, Sweden; 143 Department of Obstetrics and Gynecology, Comprehensive Cancer Center, Medical University of Vienna, A 1090 Vienna, Austria; 144 Clalit National Israeli Cancer Control Center and Department of Community Medicine and Epidemiology, Carmel Medical Center and B. Rappaport Faculty of Medicine, Haifa 34362, Israel; 145 Department of Pathology, Johns Hopkins University School of Medicine, Baltimore, Maryland, 21205, USA; 146 International Agency for Research on Cancer, 69008 Lyon, France; 147 Netherlands Cancer Institute, Antoni van Leeuwenhoek Hospital, Amsterdam 1006 BE, The Netherlands; 148 Escuela Andaluza de Salud Pública. Instituto de Investigación Biosanitaria ibs.GRANADA, Hospitales Universitarios de Granada/Universidad de Granada, 18014 Granada, Spain; 149 CIBER de Epidemiología y Salud Pública (CIBERESP), Spain; 150 Department of Environmental Health Sciences, Columbia University, New York, New York, 10032, USA; 151 Research Oncology, Division of Cancer Studies, King's College London, Guy's Hospital, London SE1 9RT, UK; 152 Center for Hereditary Breast and Ovarian Cancer, Medical Faculty, University Hospital Cologne, Cologne 50931, Germany; 153 Center for Integrated Oncology (CIO), Medical Faculty, University Hospital Cologne, 50931 Cologne, Germany; 154 Center for Molecular Medicine Cologne (CMMC), University of Cologne, 50931 Cologne, Germany; 155 Division of Genetics, Hunter Area Pathology Service, John Hunter Hospital, Newcastle, New South Wales 2305, Australia; 156 Department of Hematology and Oncology, University of Kansas Medical Center, Kansas City, Kansas 66205, USA; 157 Centre Hospitalier Universitaire de Québec Research Center, Laval University, Quebec City, Quebec, Canada G1V 4G2; 158 Unité Mixte de Génétique Constitutionnelle des Cancers Fréquents, Hospices Civils de Lyon—Centre Léon Bérard, 69373 Lyon, France; 159 Department of Pathology, The University of Melbourne, Melbourne, Victoria, Australia; 160 Hannover Medical School, 30625 Hannover, Germany; 161 Division of Clinical Genetics, Department of Clinical and Experimental Medicine, Linköping University, SE-58185 Linköping, Sweden; 162 Institut Curie, Department of Tumour Biology, 75248 Paris, France; 163 Université Paris Descartes, Sorbonne Paris Cité, 75248 Paris, France; 164 National Human Genome Research Institute, National Institutes of Health, Bethesda, Maryland 20892-2152, USA; 165 Department of Genetics, Portuguese Oncology Institute, Porto, 4200-072, Portugal; 166 Biomedical Sciences Institute (ICBAS), Porto University, 4200-072 Porto, Portugal; 167 Cancer Research Initiatives Foundation, Sime Darby Medical Centre, Subang Jaya 47500, Malaysia; 168 University Malaya Cancer Research Institute, Faculty of Medicine, University Malaya Medical Centre, University Malaya, Kuala Lumpur 50603, Malaysia; 169 Department of Epidemiology, Mailman School of Public Health, Columbia University, New York, New York, 10032, USA; 170 Department of Clinical Genetics, Odense University Hospital, 5000 Odense C, Denmark; 171 Latvian Biomedical Research and Study Centre, LV-1067 Riga, Latvia; 172 Department of Molecular Virology, Immunology and Medical Genetics, The Ohio State University, Columbus, Ohio, 43210, USA; 173 Department of Surgical Oncology, Leiden University Medical Center, Leiden 2333 ZC, The Netherlands; 174 Wellcome Trust Centre for Human Genetics and Oxford Biomedical Research Centre, University of Oxford, Oxford OX3 7BN, UK; 175 Genetic Counseling Unit, Hereditary Cancer Program, IDIBELL-Catalan Institute of Oncology, 08908 Barcelona, Spain; 176 Cancer Registry and Histopathology Unit, ‘Civic—M.P. Arezzo' Hospital, 97100 ASP Ragusa, Italy; 177 Department of Medical Oncology, Beth Israel Deaconess Medical Center, Boston, Massachusetts, 02215, USA; 178 Cancer Registry of Norway, Institute of Population-Based Cancer Research, N-0304 Oslo, Norway; 179 Department of Pathology, Family Cancer Clinic, Erasmus University Medical Center, Rotterdam 3000 CA, The Netherlands; 180 Institute of Human Genetics, Charite Berlin, 13353 Berlin, Germany; 181 Cancer Genomics Research Laboratory, Division of Cancer Epidemiology and Genetics, National Cancer Institute, Gaithersburg, Maryland 20877, USA; 182 University Hospital Ulm, 89075 Ulm, , Germany; 183 Department of Community Medicine, Faculty of Health Sciences, University of Tromsø—The Arctic University of Norway, 9037 Tromsø, Norway; 184 Genetic Epidemiology Group, Folkhälsan Research Center, 2016 Helsinki, Finland; 185 Department of Health Research and Policy—Epidemiology, Stanford University School of Medicine, Stanford, California 94305, USA; 186 Multidisciplinary Breast Center, Department of General Medical Oncology, University Hospitals, B-3000 Leuven, Belgium; 187 Division of Cancer Epidemiology and Genetics, National Cancer Institute, Bethesda, Maryland 20892, USA; 188 Servicio de Oncología Médica, Hospital Universitario La Paz, 28046 Madrid, Spain; 189 Department of Biostatistics, Harvard School Of Public Health, Boston, Massachusetts 02115, USA; 190 Cancer Division, QIMR Berghofer Medical Research Institute, Brisbane, Queensland 4029, Australia; 191 Division of Cancer Epidemiology and Genetics, National Cancer Institute, National Institutes of Health, Rockville, Maryland 20850, USA

## Abstract

Common variants in 94 loci have been associated with breast cancer including 15 loci with genome-wide significant associations (*P*<5 × 10^−8^) with oestrogen receptor (ER)-negative breast cancer and *BRCA1*-associated breast cancer risk. In this study, to identify new ER-negative susceptibility loci, we performed a meta-analysis of 11 genome-wide association studies (GWAS) consisting of 4,939 ER-negative cases and 14,352 controls, combined with 7,333 ER-negative cases and 42,468 controls and 15,252 *BRCA1* mutation carriers genotyped on the iCOGS array. We identify four previously unidentified loci including two loci at 13q22 near *KLF5*, a 2p23.2 locus near *WDR43* and a 2q33 locus near *PPIL3* that display genome-wide significant associations with ER-negative breast cancer. In addition, 19 known breast cancer risk loci have genome-wide significant associations and 40 had moderate associations (*P*<0.05) with ER-negative disease. Using functional and eQTL studies we implicate *TRMT61B* and *WDR43* at 2p23.2 and *PPIL3* at 2q33 in ER-negative breast cancer aetiology. All ER-negative loci combined account for ∼11% of familial relative risk for ER-negative disease and may contribute to improved ER-negative and *BRCA1* breast cancer risk prediction.

## Abstract (web summary)

Oestrogen negative breast cancer is associated with a poor prognosis. In this study, the authors perform a meta-analysis of 11 breast cancer genome-wide association studies and identify four new loci associated with oestrogen negative breast cancer risk. These findings may aid in stratifying patients in the clinic.

## Graphical abstract

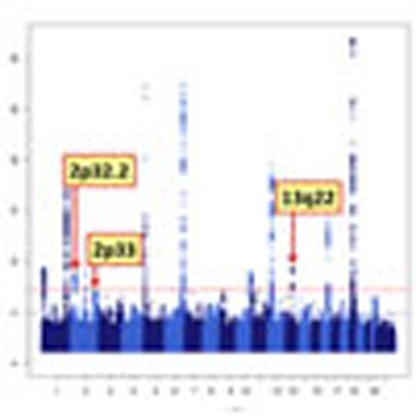

Breast cancer is a heterogeneous disease that can be separated into clinical subtypes based on tumour histological markers, such as the oestrogen receptor (ER). ER-negative disease accounts for 20–30% of all breast cancers, is more common in women diagnosed at young age and in women of African ancestry^1^, and is associated with worse short-term outcome than ER-positive disease. ER-negative and ER-positive breast cancer also exhibit different patterns of genetic susceptibility^2^. Currently, 94 loci containing common breast cancer risk-associated variants have been associated with breast cancer through genome-wide association studies (GWAS), and large replication studies^3^,^4^,^5^,^6^,^7^,^8^,^9^,^10^,^11^,^12^,^13^,^14^,^15^,^16^,^17^,^18^. However, only 14 loci have shown genome-wide significant associations (*P*<5 × 10^−8^) with ER-negative disease^3^,^17^,^18^,^19^,^20^. While this partly reflects the smaller sample size for ER-negative disease, the majority of the known breast cancer loci show differences in relative risk by subtype. In particular, 6 of the 14 loci associated with ER-negative disease at genome-wide significance show no evidence of association with ER-positive disease^20^. The alleles associated with ER-negative breast cancer^3^,^17^ at these loci have also been associated with breast cancer risk in *BRCA1* mutation carriers^21^,^22^, consistent with the finding that the majority of breast tumours arising in *BRCA1* mutation carriers show low/absent expression of ER^23^,^24^,^25^. These observations suggest that a meta-analysis of results from ER-negative breast cancer and *BRCA1* breast cancer association studies could identify additional ER-negative susceptibility loci that were not found previously because of limited sample size.

In this study, we carried out a meta-analysis of breast cancer GWAS studies and found four new loci associated with developing ER-negative breast cancer.

## Results

### Associations with ER-negative breast cancer

Genotype data for this meta-analysis were obtained from three sources: (1) 11 breast cancer GWAS included 5,139 ER-negative breast cancer cases and 14,352 controls (Supplementary Table 1); (2) The Breast Cancer Association Consortium (BCAC) included 7,333 ER-negative breast cancer cases and 42,468 study-matched controls genotyped on the iCOGS (Collaborative Oncological Gene-environment Study) custom array^3^; (3) The Consortium of Investigators of Modifiers of *BRCA1/2* (CIMBA)^26^ included 15,252 *BRCA1* mutation carriers (7,797 with breast cancer and 7,455 unaffected) genotyped on the iCOGS array (Supplementary Tables 2–4). Imputation was performed using the 1000 Genomes project as a reference^20^,^27^, and a meta-analysis was performed based on 10,909,381 common single-nucleotide polymorphisms (SNPs) that passed quality control (Supplementary Table 1).

**Figure 1:**
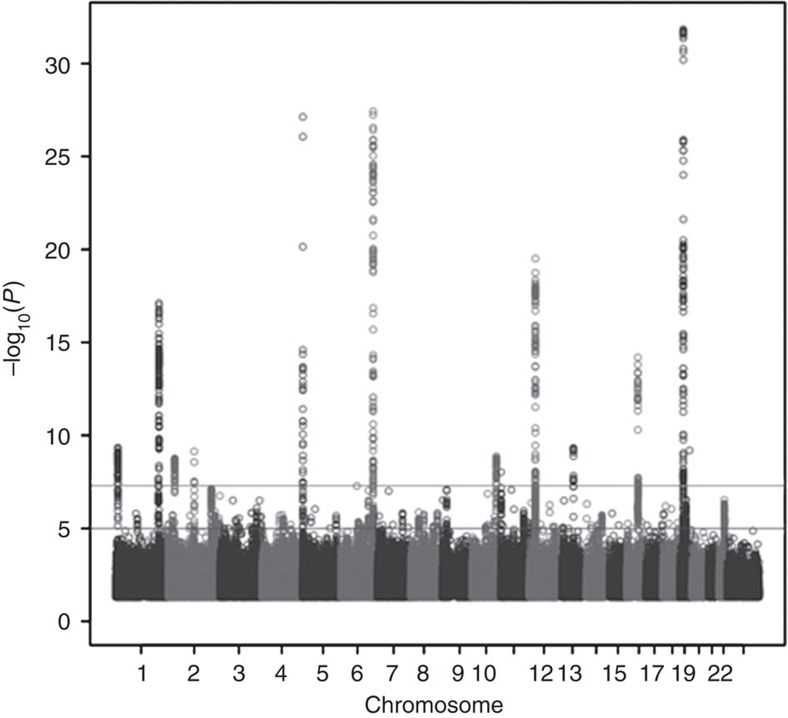
Manhattan plot of ER-negative breast cancer meta-analysis. The Manhattan plot displays the strength of genetic association (−log_10_ *P*) versus chromosomal position (Mb), where each dot presents a genotyped or imputed (black circle) SNP. The black horizontal line represents the threshold for genome-wide significance (*P*=5 × 10^−8^).

We first considered SNPs in 94 regions in which genome-wide significant associations for breast cancer had been identified (Methods)^20^. In 55 of these, the SNP most significantly associated with overall breast cancer risk was significantly associated (*P*<0.05) with ER-negative breast cancer in the meta-analysis. Four more were associated with ER-negative breast cancer in the general population (*P*<0.05) but not in the meta-analysis, and 15 displayed genome-wide significant (*P*<5 × 10^−8^) associations with ER-negative breast cancer (Supplementary Table 5). In addition, new SNPs in three loci (rs10864459 from 1p36.2 *PEX14*, rs11903787 from *INHBB* and rs4980383 from 11p15.5 *LSP1*) were found to have genome-wide significant associations with ER-negative disease (Table 1, Fig. 1, Supplementary Table 5). Likewise, SNPs in the *TCF7L2* locus previously associated with *BRCA1* breast cancer^22^ and ER-positive breast cancer^3^,^20^ showed genome-wide significant associations with ER-negative breast cancer (Table 1). Interestingly multiple independent signals in several loci were associated with ER-negative breast cancer. In particular, three independent regions in the *TERT* locus^28^, two regions in PTHLH, and two regions in ESR1 displayed genome-wide significant associations with ER-negative breast cancer (Table 1). Furthermore, while previous studies established genome-wide significant associations with ER-negative disease for rs11075995 in one 16q12.2 *FTO* locus^17^, rs17817449 (*r*^2^=0.035) from a second *FTO* locus located 40 kb proximal to the rs11075995 tagged locus^17^ also displayed near-genome-wide significance (*P*=5.26 × 10^−8^) with ER-negative breast cancer in the meta-analysis (Table 1). In addition to the breast cancer loci established in studies of European women, three additional breast cancer risk loci were recently identified in GWAS of Asian women. To generalize the results to other populations, associations between the three SNPs and breast cancer in the European, African American and Asian populations in the iCOGS study were evaluated. SNP rs2290203 showed only weak evidence of association (*P*=0.02), and rs4951011 and rs10474352 SNPs showed no evidence of association with ER-negative breast cancer in the white European meta-analysis (Supplementary Table 6).

Among the 94 known risk loci from white European and three from Asian populations, only 24 contained SNPs with some evidence of association (*P*<0.05) with breast cancer risk among *BRCA1* mutation carriers alone. These included 21 loci based on known index SNPs (Supplementary Table 5) along with new SNPs from the meta-analysis in the *PEX14* (rs10864459), *INHBB* (rs11903787) and *PTHLH* (rs7297051) loci (Table 1). Only the *ESR1* (rs2046210), *TERT* (rs2242652) and two 19p13.1 (rs8170; rs56069439) loci had genome-wide significant associations with breast cancer risk for *BRCA1* mutation carriers alone (Table 1, Supplementary Table 5). However, 15 of the 19 risk loci that reached genome-wide significance for ER-negative disease in the meta-analysis showed some evidence of association (*P*<0.05) with breast cancer risk for *BRCA1* mutation carriers using a retrospective likelihood analysis^12^. These SNPs had hazard ratio (HR) estimates in *BRCA1* carriers that were similar to the odds ratio (OR) estimates for ER-negative breast cancer (Table 1). In contrast, four SNPs in the *LGR6*, *2p24.1*, *ZNF365* and *FTO* loci had HR estimates ranging from 0.97 to 1.01 and were not significantly associated (*P*>0.05) with breast cancer risk for *BRCA1* mutation carriers. No significant interactions between the known risk SNPs were observed when pairwise interactions were evaluated separately in the general population (BCAC-iCOGS) or in *BRCA1* carriers after adjusting for multiple testing.

### Genome-wide associations with ER-negative breast cancer

**Figure 2:**
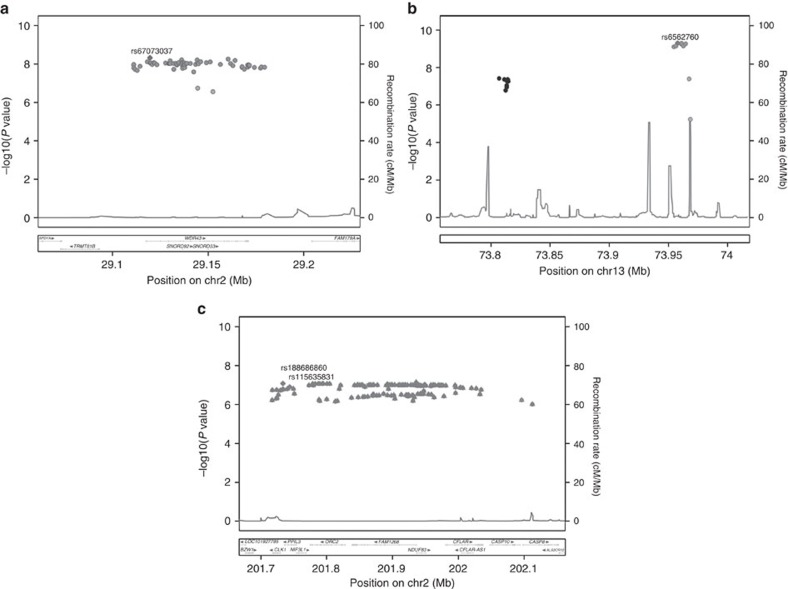
Novel ER-negative breast cancer loci. The chromosomal position and strength of genetic association (−log_10_ *P*) is shown for all SNPs (*P*<1 × 10^−6^) in BCAC/iCOGS data in the four novel risk loci. (**a**). 2p23 locus. The most significant SNP (rs67073037) is shown as a diamond. (**b**). 13q22 loci. The most significant SNP (rs6562760) is shown as a diamond. The second locus is shown in black. (**c**). 2q33 locus. The most significant SNPs (rs188686860; rs115635831) are shown as diamonds.

Novel genome-wide significant associations (*P*<5 × 10^−8^) were detected with imputed and genotyped SNPs on chromosomes 2p23.2 and 13q22 (Table 2, Fig. 2, Supplementary Fig. 1). At 2p23.2, 79 SNPs exhibited genome-wide significant associations with ER-negative breast cancer (Fig. 2, Supplementary Fig. 2, Supplementary Table 7). The most significant genotyped and imputed SNPs at these two loci were rs4577244 (*P*=1.0 × 10^−8^) and rs67073037 (*P*=4.76 × 10^−9^), respectively (Table 2). To investigate the presence of independent signals at the 2p23.2 locus, conditional analyses were conducted adjusting for the lead SNP. However, no significant (*P*<0.05) associations were observed at 2p23.2 after adjusting for rs67073037. In the 13q22 locus, rs6562760 was the most strongly associated (*P*=5.0 × 10^−10^) SNP among 12 genome-wide significant SNPs (Table 2, Supplementary Table 8, Fig. 2, Supplementary Fig. 1). Conditional analysis adjusting for rs6562760 yielded several SNPs with residual associations for ER-negative breast cancer, with rs17181761 (*r*^2^=0.51) as the most significantly associated (*P*=6.0 × 10^−6^) (Supplementary Table 9). No associations at *P*<10^−4^ remained after conditioning on both rs6562760 and rs17181761. Thus, 13q22 appears to contain two independent ER-negative risk loci.

When considering only the data from the general population using the BCAC-iCOGS studies, no association between rs67073037 at 2p23.2 and ER-positive breast cancer was observed (Supplementary Table 10). Consistent with this observation, a significant difference (*P*_diff_=4.45 × 10^−6^) in the per-allele ORs for ER-positive and ER-negative breast cancer was detected. In contrast, rs17181761 at 13q22 was weakly associated with ER-positive breast cancer (OR=1.03; *P*=0.030), but more strongly associated with ER-negative breast cancer (OR=1.08; *P*_diff_=5.82 × 10^−3^; Supplementary Table 10). Likewise, rs6562760 at 13q22 was more strongly associated with ER-negative than ER-positive breast cancer (ER-positive OR=0.98 versus ER-negative OR=0.92; *P*_diff_=0.028) (Supplementary Table 10). Among ER-negative cases, no significant differences in the ORs for triple negative (ER-negative, progesterone receptor negative, HER2 negative) and non-triple-negative cases was observed (rs67073037, *P*_diff_=0.26; rs6562760, *P*_diff_=0.36; rs17181761, *P*_diff_=0.69). Q-tests were used to assess heterogeneity. These results suggest that the three risk loci are largely specific to ER-negative but not triple-negative breast cancer, in contrast to loci in the *MDM4*, *LGR6*, 19p13.1 and *TERT* regions^3^,^17^. To also investigate the impact of bilateral disease on the associations with ER-negative breast cancer in the general population, analyses were performed separately for BBCS alone, which oversampled for bilateral cases, and after exclusion of BBCS. The risk estimates for each SNP (both in iCOGS and in the meta-analysis), after excluding BBCS, did not differ from the main results (Supplementary Table 11), and do not appear to be substantially influenced by bilateral cases.

Using the retrospective likelihood approach, index SNPs in the three 2p23.2 and 13q22 loci were all associated with *BRCA1* breast cancer (rs67073037, *P*=4.58 × 10^−4^; rs6562760, *P*=2.85 × 10^−6^; rs17181761, *P*=9.29 × 10^−3^; Table 2). There were no significant differences in the associations with ER-positive and ER-negative disease among *BRCA1* carriers (Supplementary Table 12). A competing risks analysis in *BRCA1* mutation carriers that accounted for simultaneous associations with breast and ovarian cancer risks found similar HR estimates for breast cancer and no evidence of association with ovarian cancer risk (Supplementary Table 13). None of the SNPs were associated with overall breast cancer risk for *BRCA2* mutation carriers (Supplementary Table 10). There was also no significant evidence of heterogeneity (*P*<0.05) between the effect estimates for *BRCA1* mutation carriers and ER-negative breast cancer in the general population (BCAC-iCOGS; Intraclass Correlation)^27^. Finally, no significant interactions between the three index SNPs and any of the 94 previously known loci were observed in *BRCA1* carriers or in the general population after adjusting for multiple testing (Supplementary Table 14).

### Association with ER-negative breast cancer in the 2q33 locus

Analysis of genotyped and imputed SNPs around known risk loci also detected near-genome-wide significant associations with ER-negative breast cancer in a region on 2q33 containing several genes including *PPIL3* and the known *CASP8* risk locus (Table 2). rs115635831 (*P*=1.26 × 10^−7^) and rs188686860 (*P*=8.34 × 10^−8^; *r*^2^=1.0), were the genotyped and imputed SNPs, respectively, most significantly associated with ER-negative breast cancer in this region. These SNPs, along with the most proximal rs74943274 SNP (*r*^2^=0.97 with rs115635831), are located in *CLK1* (Cdc-like kinase-1) and *PPIL3* (Peptidylproplyl isomerase-Like 3) and are 350 kb upstream of *CASP8* (Table 2, Fig. 2). All 157 SNPs with highly significant associations (*P*<1 × 10^−6^) in this region, were in high linkage disequilibrium with rs188686860 and rs115635831 (*r*^2^>0.90), and were located proximal (Hg19: 201,717,014-201,995,860) to the *CASP8* gene (Supplementary Table 15). Fine mapping of the *CASP8* locus has recently identified four independent signals associated with overall breast cancer risk^29^. The index SNPs for these independent signals range across a 350-kb region from 202,036,478 to 202,379,828. To determine whether these *CASP8*-associated signals accounted for the ER-negative associations in the meta-analysis, conditional analyses were conducted using the BCAC-iCOGS data. After accounting for the four *CASP8* signals, rs74943274 retained evidence of an association with overall breast cancer (*P*=1.44 × 10^−3^) and a strong association with ER-negative breast cancer (*P*=1.34 × 10^−5^; Supplementary Table 16; Supplementary Fig. 2), suggesting that rs74943274 and rs115635831 represents a novel locus associated with ER-negative breast cancer.

Further consideration of the BCAC-iCOGS data found no association for rs115635831 at 2q33 with ER-positive breast cancer (*P*=0.23) but identified a significant difference (*P*_diff_=2.9 × 10^−4^) in the per-allele ORs for ER-positive and ER-negative breast cancer (Q-test, Supplementary Table 10). No influence of bilateral disease was observed in sensitivity analyses (Supplementary Table 11). However, the index SNPs in the 2q33 locus were significantly associated with *BRCA1* breast cancer (rs115635831, *P*=0.018; rs188686860, *P*=0.012; Table 2). While there were no significant differences in the associations with ER-positive and ER-negative disease among *BRCA1* carriers (*P*Het=0.12), the associations were stronger for ER-negative (rs115635831 HR=1.32, *P*=3 × 10^−3^) than ER-positive breast cancer (rs115635831 overall HR=1.21, *P*=0.018) using the retrospective likelihood model (Supplementary Table 12). In addition, the associations for *BRCA1* mutation carriers were of similar magnitude as the OR estimates for ER-negative breast cancer in BCAC-iCOGS^27^ (Supplementary Table 15). There was also no evidence of intraclass heterogeneity (*P*<0.05) between the effect estimates for *BRCA1* mutation carriers and ER-negative breast cancer in the general population (BCAC-iCOGS)^27^. A competing risks analysis for *BRCA1* mutation carriers found little influence of ovarian cancer on risks of breast cancer (rs115635831 HR=1.23, *P*=0.016), and no evidence of association with ovarian cancer risk using the retrospective likelihood model (Supplementary Table 13). No association with overall breast cancer risk among *BRCA2* mutation carriers (Supplementary Table 10) was evident. Interestingly, rs114962751 at 2q33 and rs150750171 at 6p had the most significant interaction (*P*=3.9 × 10^−4^) among all known breast cancer risk SNPs in the iCOGS data, although the interaction was non-significant after adjusting for multiple testing (Supplementary Table 14). Altogether these results suggest the presence of a novel locus associated with ER-negative breast cancer that is located in the *CLK1/PPIL3* region proximal to *CASP8*.

### Expression quantitative trait locus (eQTL) analysis

To identify the genes in the novel loci influenced by the observed associations with ER-negative breast cancer, expression quantitative trait locus (eQTL) analyses were performed using gene expression data from breast tumour tissue and normal breast tissue and 1000 Genomes Project imputed SNPs in 1 Mb regions around the novel loci. In the 2p23.2 locus, the strongest cis eQTL associations for 735 TCGA breast tumours (BC765) involved *TRMT61B* expression (Supplementary Table 17). Most of the genome-wide significant ER-negative breast cancer risk SNPs in the locus displayed associations with *TRMT61B* expression, including the imputed SNPs (rs67073037, *P*=1.47 × 10^−5^; Supplementary Fig. 3; rs6734079, *P*=1.85 × 10^−5^) and the genotyped SNP (rs4577254, *P*=5.61 × 10^−5^) most significantly associated with risk (Supplementary Table 18). Similarly, in a Norwegian normal breast cohort of 116 normal breast tissues (NB116), the strongest cis eQTLs associations involved *TRMT61B* expression and the risk SNPs in the locus yielded significant associations with *TRMT61B* expression (Supplementary Table 17). While the peak eQTL SNPs (rs6419696, *P*=1.21 × 10^−17^) were not among the SNPs showing the greatest association with risk (rs6419696, *P*=2.6 × 10^−3^), conditional analyses showed that the rs6419696 eQTL SNP accounted for much of the influence of the rs4577254 SNP on ER-negative breast cancer risk (*P*=9.07 × 10^−4^) and vice versa (Supplementary Table 18). Thus, modulation of *TRMT61B* expression may contribute in part to the risk of breast cancer in this region. In the 13q22.1 locus, the strongest eQTLs in the 735 TCGA breast tumours (BC765) involved *PIBF1* (Supplementary Table 19). However, none of the SNPs strongly associated with breast cancer risk in either of the two independent 13q22 loci showed associations with gene expression (Supplementary Table 19, Supplementary Fig. 4). In contrast, significant associations with *DIS3* expression were observed in the BC241 and NB116 cohorts for many of the genome-wide significant SNPs in the locus represented by rs17181761 (NB116 eQTL *P*=2.34 × 10^−3^) (Supplementary Table 19). While non-significant after accounting for multiple testing, these observations suggest that future studies should evaluate mechanistic interactions between 13q22.1 SNPs and *DIS3* expression. Evaluation of eQTLs in the 2q33 locus for the BC765 cohort found that many of the 157 risk-associated SNPs (Table 2, Supplementary Table 15) had strong associations with *PPIL3* expression (rs188686860, *P*=1.77 × 10^−7^; rs115635831, *P*=6.08 × 10^−7^; Supplementary Fig. 5) and little evidence of any associations with other genes in the region (Supplementary Table 20). This is one of the few known breast cancer risk loci where the most significant risk SNPs are strongly associated with local gene expression. *PPIL3* is located at the proximal end of the locus, 270 kb upstream of *CASP8*, further suggesting that the 2q33 risk locus is independent of any influence on *CASP8*.

### Functional characterization of the 2p23.2 locus

To identify candidate SNPs and genes in the 2p23.2 locus driving ER-negative breast cancer risk, ENCODE chromatin biofeatures were evaluated in primary human mammary epithelial cells (HMECs), MCF7 ER-positive cells and MB-MDA-231 ER-negative cells^30^. Sixteen of the 79 most significantly associated SNPs (*P*<3 × 10^−7^) in the region overlapped with three distinct regulatory regions (Supplementary Figs 6 and 7). The most significantly associated ER-negative SNP, rs67073037 (Table 2) was located in intron 1 of *WDR43* near the transcription start site in a region containing acetylated H3K27 and trimethylated H3K4 chromatin marks in normal HMECs and MB-MDA-231 ER-negative breast tumour cells, and a DNase hypersensitivity cluster in ER-positive MCF7 cells (Supplementary Figs 6 and 7). The three risk-associated SNPs (rs4407214, rs66604446 and rs66768547) with the most significant RegulomeDB scores (2b), were located in the same chromatin marks in this region in HMEC, MD-MBA-231 and MCF7 cells (http://regulomedb.org). In addition, the top genotyped SNP (rs4577244) was located in a monomethylated H3K4 mark adjacent to the core promoter region of *WDR43* in HMECs (Supplementary Fig. 6). Separately rs11677283 and rs35617956 in introns 9 and 10 of *WDR43* were located in acetylated H3K27 and H3K9 chromatin marks in a putative regulatory region in HMECs, but not in ER-negative MD-MBA-231 cells.

**Figure 3:**
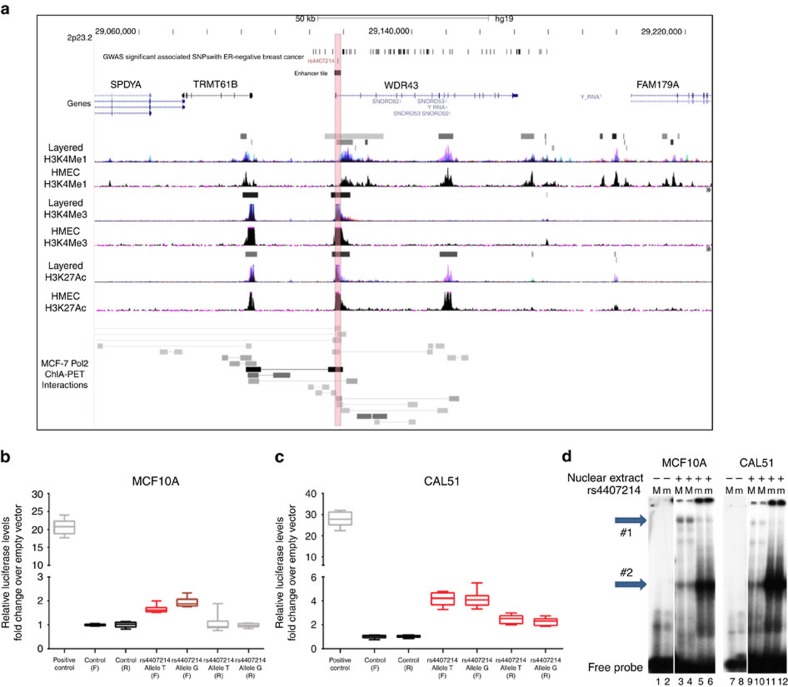
The chromatin landscape of locus 2p23.2. (**a**) The SNP rs4407214 is included in a genomic tile overlapping chromatin features indicative of promoters and enhancers, shaded red. (**b**,**c**). Luciferase assays showing activity in the tile containing SNP rs4407214 (highlighted in pink in **a**.) in MCF10A and CAL51, red box plots indicate significantly different from the control tile (*P*<0.0001). Brown box plot indicates significant difference from the reference allele (*P*= 0.0059). (**d**) Electrophoretic mobility shift assay (EMSA) showing the formation of allele-specific complexes for rs4407214. M, major allele; m, minor allele. Lines 1, 2, 7, 8—no nuclear extract. Lines 3, 4, 5, 6—10 μg of MCF10A nuclear extract. Lines 9, 10, 11, 12—10 μg of CAL51 nuclear extract. Shift detected by comparison to bands (arrows #1 and #2).

Combining the eQTL results with these predictions, we tested four genomic tiles spanning region 1 for enhancer activity in both orientations using a luciferase reporter assay in the CAL51 ER-negative breast cancer line and MCF10A normal mammary epithelial cells (Fig. 3). The tile containing rs4407214 displayed significant enhancer activity (*P*<0.0001) in at least one orientation when compared with the negative control in MCF10A and CAL51 (Fig. 3). In addition, the tile carrying the disease-associated G allele showed significantly (*P*=0.0059) higher activity than the T allele in MCF10A cells (Fig. 3). Similarly, the disease-associated G-allele showed significantly (*P*=0.0059) higher activity than the T-allele in a luciferase-based promoter assay in MCF10A cells (*P*=0.044) and CAL51 (*P*=0.0078; Supplementary Fig. 8). Consistent with these allele-specific changes in transcriptional activity different protein complexes in electrophoretic mobility shift assays were observed using CAL51 and MCF10A nuclear extracts (Fig. 3). In addition, Pol2 ChIA-PET in MCF7 breast cancer cells revealed an interaction between Region 1 and the promoter of *TRMT61B* (Fig. 3), which had the strongest eQTL signal in the locus. These results are consistent with modification of Pol2 binding to this region by rs4407214 in lymphoblastoid cells^31^ and suggest the presence of a transcriptional enhancer in the region. Separately, the ChIA-PET data further suggest that Region 2 in *WDR43* may interact with the promoter of *WDR43* (Fig. 3). Thus, *WDR43* and *TRMT61B* may be regulated by interactions of enhancers in *WDR43* with the core *WDR43* and *TRMT61B* promoters and may jointly influence breast cancer risk in this region.

### Functional characterization of the 13q22 locus

The SNPs most significantly associated with ER-negative breast cancer in the two 13q22 loci formed two small clusters in a 4-kb region around rs17181761 and a 10-kb region around rs8002929. Bioinformatics analysis and chromatin feature analysis identified weak DNaseI hypersensitivity sites, CTCF binding and monomethylated H3K4 sites in both regions in HMEC cells, consistent with weak enhancer activity (Supplementary Figs 9 and 10). Both rs17181761 and rs12870942 in the proximal locus are associated with transcriptional activity in HMECs, whereas rs8002929 and rs927683 in the distal locus are associated with enhancer and DNAse hypersensitivity sites in HMECs, respectively (http://regulomedb.org). Both 13q22 loci are located in a non-genic 600-kb region between the *KLF5* and *KLF12* kruppel-like transcription factor genes. This segment of chromosome 13 is frequently deleted in a spectrum of cancers^32^,^33^. GWAS have also identified a pancreatic cancer risk locus in the region between *KLF5* and *KLF12* (refs 34, 35, 36). However, the rs9543325 SNP from the pancreatic cancer studies was only marginally associated with ER-negative breast cancer risk (*P*=0.03) in the meta-analysis suggesting that the signals are independent.

### Functional characterization of the 2q33 locus

The SNPs most significantly associated with ER-negative breast cancer in the 2q33 locus range across a 350-kb region that contains nine genes (Supplementary Fig. 6). This region contains at least 10 strong enhancer regions in HMECs and 12 strong enhancer regions in MD-MBA-231 cells associated with acetylated H3K27 and trimethylated H3K4 chromatin marks. As noted above, many of the 157 SNPs most significantly associated with ER-negative breast cancer are associated with *PPIL3* expression. Seven of these also scored as functional candidates by RegulomeDB (score=3a; rs17467658, rs17383256, rs17467916, rs114567273, rs76377168, rs116509920 and rs116724456). Of these rs17467658 in *CLK1* and rs17383256 in the *ORC2* gene are located in DNAse hypersensitivity sites and strong enhancer regions in HMEC and MD-MBA-231 cells (http://www.roadmapepigenomics.org; Supplementary Figs 11 and 12). In addition, rs116509920 and rs116724456 are associated with *PPIL3* expression (*P*=5.85 × 10^−7^), although neither SNP is associated with an enhancer or suppressor region. The genotyped SNP most significantly associated with risk, rs114962751, is located in acetylated H3K27 and trimethylated H3K4 chromatin marks in a bidirectional promoter for *FAM126B* and *NDUFB3* in HMEC and MD-MBA-231 cells (Supplementary Figs 11 and 12). Similarly, the rs74943274 genotyped risk SNP (Table 2) is located near the 3′-untranslated region of *CLK1* and is associated with *PPIL3* expression (*P*=2.37 × 10^−6^). However, rs78258606 is perhaps a more likely candidate driver of ER-negative risk in this locus because the SNP is associated with ER-negative breast cancer (*P*=1.9 × 10^−7^), is located in the *CLK1* promoter in acetylated H3K27 and trimethylated H3K4 chromatin marks in HMEC and MD-MBA-231 cells and DNase hypersensitivity sites in MCF7 cells, and is associated with *PPIL3* expression (*P*=2.71 × 10^−7^) (Supplementary Figs 11 and 12). Further fine mapping and functional characterization of this locus is needed to resolve the underlying functional effects and identify the genes influencing ER-negative breast cancer risk.

## Discussion

When including the four 2p23.2, 13q22 and 2q33 novel loci identified in this meta-analysis, 23 independent loci have shown genome-wide significant associations with ER-negative disease, including 10 loci showing no associations or only weak associations with ER-positive disease. In total, 63 loci have shown at least marginal significance (*P*<0.05) with ER-negative breast cancer. In *BRCA1* mutation carriers, 27 independent loci (*P*<0.05) have been associated with modified breast cancer risk^27^. The percentage of the familial risk for ER-negative disease explained by SNPs is not well defined because there is currently no good estimate for the familial relative risk for ER-negative disease. However, assuming that the estimate is similar to that for overall breast cancer (twofold for a first-degree relative), and based on the estimated frequencies and ORs from the iCOGS data, the SNPs in the known breast cancer risk loci explain 9.8% of the familial risk and the SNPs in the four new loci account for a further 0.8%. The addition of these new ER-negative loci may improve overall risk prediction models for ER-negative disease in the general population and for breast cancer among *BRCA1* mutation carriers by enhancing the contribution of current polygenic risk prediction models^21^,^22^. Furthermore, fine mapping and functional studies of these loci may provide further insight into the aetiology of ER-negative breast cancer.

## Methods

### Study populations

Details of the subjects, genotyping and quality control measures for the BCAC GWAS and iCOGS data^3^, BPC3 (ref. 16), EBCG^37^, TNBCC^14^,^38^ and *BRCA1* (ref. 22) are described elsewhere. Analyses were restricted to women of European ancestry. Overall, 42 BCAC studies provided the iCOGS genotyping data for ER-negative breast cancer cases and controls. In addition, 11 breast cancer studies provided GWAS genotyping data. Forty five CIMBA studies provided iCOGS genotyping on 15,252 *BRCA1* mutation carriers, of whom 7,797 were affected with breast cancer.

### Genotype data

Genotyping and imputation details for each study are shown in Supplementary Table 1.

### Imputation

We performed imputation separately for *BRCA1* carriers, 11 GWAS, BCAC-iCOGS and TNBCC-iCOGS samples. We imputed variants from the 1000 Genomes Project data using the v3 April 2012 release^39^ as the reference panel. Imputation was based on the 1000 Genomes Project data with singletons removed. Eight BCAC GWAS were imputed in a two-step procedure, with prephasing using the SHAPEIT software and imputation of the phased data in the second with IMPUTEv2 (ref. 40). For the remaining three GWAS (BPC3, TNBCC and EBCG), imputation was performed using MACH (version 1.0.18) and Minimac (version 2012.8.15)^41^. The iCOGS data were also imputed with two-stage procedure involving SHAPEIT and IMPUTEv2. To perform the imputation we divided the data into segments of ∼5 Mb each. The iCOGS samples were divided into 10 subsets, keeping all subjects from individual studies in the same set. Estimates and s.e.'s were obtained using logistic regression adjusting for study and 9 principal components. GWAS SNPs were excluded if the imputation accuracy was *r*^2^<0.3 or if the minor allele frequency (MAF) was <0.01, TNBCC SNPs were excluded when the imputation accuracy was *r*^2^<0.9 and MAF<0.05, iCOGS SNPs were excluded when *r*^2^<=0.3 and MAF<0.005. Regions with evidence of genome-wide significant associations (*P*<5 × 10^−8^) were reimputed in iCOGS, using IMPUTEv2 but without prephasing in SHAPEIT to improve imputation accuracy. In addition, the number of MCMC iterations were increased from 30 to 90, and the buffer region was increased to ±500 kb from any significantly associated SNP in the region.

### Meta-analysis

A fixed effects meta-analysis of ER-negative breast cancer associations was conducted using an inverse variance approach assuming fixed effects, as implemented in METAL^42^. The effect estimates used were the logarithm of the per-allele HR estimate for the association with breast cancer risk in *BRCA1* and *BRCA2* mutation carriers and the logarithm of the per-allele OR estimate for the association with breast cancer status in GWAS and iCOGS analyses, both of which were assumed to approximate the same relative risk. For the associations in *BRCA1* mutation carriers, a kinship-adjusted variance estimator was used^12^. *P*-values were estimated by *z*-test.

### Heterogeneity analysis

Heterogeneity across estimates from BCAC and iCOGS were evaluated using a Cochran Q test and *I*^2^ for the proportion of total variability explained by heterogeneity in the effect sizes^43^. Associations with ER-positive and ER-negative subgroups of *BRCA1* carriers were evaluated using an extension of the retrospective likelihood approach to model the simultaneous effect of each SNP on more than one tumour subtype^27^. The consistency between breast cancer associations for breast cancer susceptibility variants in the general population and associations in *BRCA1* and *BRCA2* carriers were evaluated using the intraclass correlation (ICC)^27^. The ICC was estimated based on a one-way random-effects model and tested for agreement in absolute values of log HR.

### Locus coverage

Locus boundaries were defined so that all SNPs with *r*^2^⩾0.1 with the most significantly associated SNP were included. SNPs with MAF<0.005 were excluded. Linkage disequilibrium blocks were defined at *r*^2^⩾0.8. Each linkage disequilibrium block was evaluated for the presence of at least one genotyped or imputed SNP. If imputed, then the imputation accuracy was considered.

### Expression quantitative trait locus analysis

eQTL analysis was performed for all protein coding genes within 1 Mb, up- and downstream of the SNP most significantly associated with ER-negative breast cancer risk in each locus. Normal breast (NB116; *n*=116) and breast cancer (BC241, *n*=241) are comprised of women of Norwegian descent. Gene expression data for the majority of women in NB116 were derived from normal breast tissue in women who had not been affected with breast cancer; data for ten women were derived from normal tissue adjacent to a tumour. Gene expression data for BC241 were derived from breast tumours (70 ER-negative and 170 ER-positive). Genotyping was performed with the iCOGS SNP array, and gene expression levels were measured with the Agilent 44K array^44^,^45^. BC765 (*n*=765) is the TCGA breast cancer cohort composed of 139 ER-negative, 571 ER-positive and 55 undefined breast tumours; all non-European samples (as determined by clustering and PCA) were excluded from this analysis^46^. Germline genotype data from Affymetrix SNP 6 array were obtained from TCGA dbGAP data portal^46^. Gene expression levels for the breast tumours were assayed by RNA sequencing, RSEM (RNaseq by Expectation-Maximization21) normalized per gene, as obtained from the TCGA consortium portal^46^. The data were log2 transformed, and unexpressed genes were excluded prior to eQTL analysis. There is no overlap between women recruited to each of these studies. The genotyping data were processed as follows: SNPs with call rates <0.95 or minor allele frequencies <0.05 or Hardy–Weinberg equilibrium (*P*<10^−13^) were excluded. Samples with call rates below 80% were excluded. Identity by state was computed with the R GenABEL package^47^ and closely related samples with IBS>0.95 were removed. Imputation was performed on the iCOGS and Affymetrix6 germline genotype data using the 1000 Genomes Project March 2012 v.3 release as the reference data set. A two-stage imputation procedure was used as described above. The influence of SNPs on gene expression was assessed using a linear regression model. An additive effect was assumed by modelling copy number of the rare allele, that is, 0, 1 or 2, for a given genotype.

### Candidate gene analysis

TCGA has performed extensive genomic analysis of tumours from a large number of tissue types including over 1,000 breast tumours. All genes in the novel loci were evaluated for coding somatic sequence variants in TCGA. Breast tumours with log2 copy-number data in the TCGA data were analysed for deletion and amplification of each candidate gene using the cBio portal^48^,^49^.

### Informatics and chromatin biofeatures

Candidate SNPs were evaluated using SNPInfo (http://snpinfo.niehs.nih.gov) and SNPnexus (http://snp-nexus.org/test/snpnexus). The presence of SNPs in transcription factor binding sites using TRANSFAC and miRNA binding sites using TargetScan were noted. Regulatory potential scores (ESPERR Regulatory Potential) were obtained from the UCSC genome bioinformatics browser (http://genome.ucsc.edu/). RegulomeDB (http://regulomedb.org) was used to assess SNPs for transcription factor recognition motifs, open chromatin structure based on FAIRE and DNAse-seq analysis and protein binding sites based on ChIP-seq data. Chromatin biofeatures in HMEC and MCF7 cells were assessed using ENCODE layers on the UCSC browser (http://genome.ucsc.edu/). Enhancers active in the mammary cell types MCF7 and HMEC were cross-referenced with candidate SNPs.

### Luciferase reporter assays

Genomic tiles spanning regions containing SNPs with indication of regulatory activity by RegulomeDB were generated. Regions containing the major and minor alleles within the 2p23.2 region spanning 2,229 bp (chr2:29,117,333-29,119,561) were generated by PCR using BAC DNA CTD-3216P10 as template. Forward and reverse primers contained *att*B1 and *att*B2 sequences, respectively, to aid in recombinational cloning. Tiles were cloned in both a forward and reverse orientation upstream of the SV40 promoter by recombination in the firefly luciferase reporter vector pGL3-Pro-attb vector designed to test for enhancer regions. This vector is a modification of pGL3-Promoter (Invitrogen) adding *att*B sites surrounding the *ccdb* gene. The clone containing the tile was co-transfected in eight replicates using LipoFectamine 2000 (Life Technologies) into MCF10A or CAL51 cells with pRL-CMV (Promega), an internal control expressing Renilla luciferase, per well of 96-well plates. Luciferase activity was measured 24-h post transfection by Dual Glo Luciferase Assay (Promega). Transfections were repeated in two independent experiments with similar results. The influence of the common and rare alleles of rs4407214 on promoter activity in the pGL3-Promoter vector (Invitrogen) were assessed using the same methodology. Primers are available on request.

### Electromobility shift assays

Nuclear proteins from MCF10A and CAL51 cells were extracted using a hypotonic lysis buffer (10 mM HEPES, pH 7.9, 1.5 mM MgCl2, 10 mM KCL) supplemented with DTT and protease inhibitors, followed by an extraction buffer (20 mM HEPES, ph 7.9, 1.5 mM MgCl_2_, 0.42 M NaCl, 0.2 mM EDTA, 25% v/v glycerol) supplemented with DTT and protease inhibitors. Electrophoretic mobility shift assays probes were designed to cover each SNP ±20 base pairs, for both major and minor alleles. Probe pairs were dissolved in water and annealed at a concentration of 10 μM each. Probes were labelled with ATP (γ-32 P; Perkin Elmer) using T4 polynucleotide kinase and cleaned using the QiaQuick Nucleotide Removal Kit (Qiagen). Labelled and unlabelled probes were then incubated with protein extracts using LightShift Poly(dI–dC) (Thermo) and a binding buffer (10 mM Tris, 50 mM KCl, 1 mM DTT, pH 7.4) and electrophoresed on a 6% acrylamide gel overnight at 83 V. Gels were dried and films were exposed for 4–24 h. Probe sequences are shown in Supplementary Table 21.

## Additional information

**How to cite this article**: Couch, F. J. *et al*. Identification of four novel susceptibility loci for oestrogen receptor negative breast cancer. *Nat. Commun.* 7:11375 doi: 10.1038/ncomms11375 (2016).

## Supplementary Material

Supplementary Information Supplementary Figures 1-12, Supplementary Tables 1-2 and Supplementary Note.

## Figures and Tables

**Table 1:** Common genetic variants from known breast cancer susceptibility loci displaying most significant genome-wide associations with ER-negative breast cancer risk.

**Location**	**Position**	**Nearest gene**	**SNP**	**Alleles**	**iCOGS/GWAS ER-negative**			**BRCA1 carriers**			**Meta-analysis**
					**EAF**	**OR (95% CI)**	***P***	**EAF**	**HR (95% CI)**	***P***	P*
Variants in known loci most significantly associated with overall breast cancer											
†1p36.2	10563609	*PEX14*	rs10864459	G/A	0.32	0.90 (0.87–0.93)	2.13 × 10^−9^	0.31	0.95 (0.91–0.99)	0.01	4.60 × 10^−10^
†1q32.1	202179042	*LGR6*	rs17489300	A/C	0.4	0.90 (0.87–0.93)	9.37 × 10^−10^	0.39	0.97 (0.93–1.01)	0.19	1.98 × 10^−8^
1q32.1	204518842	*MDM4*	rs4245739	A/C	0.26	1.13 (1.11–1.19)	5.53 × 10^−15^	0.28	1.09 (1.05–1.14)	6.83 × 10^−5^	7.71 × 10^−18^
2p24.1	19184284	*2p24.1*	rs12710696	C/T	0.36	1.10 (1.06–1.13)	1.70 × 10^−8^	0.39	1.01 (0.97–1.05)	0.56	1.90 × 10^−6^
†2q14.2	121088182	*INHBB*	rs11903787	G/A	0.25	0.90 (0.86–0.94)	8.57 × 10^−7^	0.26	0.91 (0.87–0.96)	2.0 × 10^−4^	7.24 × 10^−10^
†5p15.3	1280028	*TERT*	rs2242652	A/G	0.20	1.18 (1.13–1.23)	2.73 × 10^−14^	0.22	1.22 (1.16–1.28)	2.53 × 10^−15^	7.58 × 10^−28^
5p15.3	1282319	*TERT*	rs7726159	A/C	0.34	1.09 (1.05–1.13)	2.19 × 10^−6^	0.35	1.07 (1.02–1.11)	1.79 × 10^−3^	3.31 × 10^−8^
5p15.3	1297488	*TERT*	rs2736108	T/C	0.29	0.89 (0.86–0.93)	1.41 × 10^−8^	0.29	0.89 (0.86–0.93)	4.05 × 10^−7^	3.05 × 10^−14^
6q25.1	151918856	*ESR1*	rs12662670	T/G	0.08	1.20 (1.18–1.32)	8.90 × 10^−15^	0.09	1.19 (1.11–1.27)	9.67 × 10^−7^	1.32 × 10^−19^
†6q25.1	151946152	*ESR1*	rs11155804	A/T	0.34	1.16 (1.12–1.19)	8.18 × 10^−18^	0.36	1.15 (1.11–1.20)	0.02	3.75 × 10^−28^
10q21.2	64278682	*ZNF365*	rs10995190	G/A	0.16	0.89 (0.85–0.93)	3.75 × 10^−8^	0.16	0.99 (0.94–1.04)	0.66	8.23 × 10^−6^
†10q25.2	114782803	*TCF7L2*	rs6585202	T/C	0.46	1.06 (1.04–1.10)	3.35 × 10^−5^	0.47	1.10 (1.05–1.14)	6.08 × 10^−6^	1.32 × 10^−9^
†11p15.5	1902097	*LSP1*	rs4980383	C/T	0.44	1.08 (1.05–1.12)	3.02 × 10^−6^	0.45	1.07 (1.03–1.11)	7.73 × 10^−4^	9.41 × 10^−9^
†12p11.2	28174817	*PTHLH*	rs7297051	C/T	0.24	0.86 (0.83–0.89)	1.48 × 10^−14^	0.23	0.89 (0.85–0.93)	2.89 × 10^−7^	3.12 × 10^−20^
12p11.2	28155080	*PTHLH*	rs10771399	A/G	0.12	0.79 (0.78–0.87)	3.82 × 10^−13^	0.10	0.86 (0.80–0.91)	2.55 × 10^−6^	7.18 × 10^−18^
†16q12.1	52599188	*TO* × *3*	rs4784227	C/T	0.24	1.15 (1.11–1.19)	1.11 × 10^−14^	0.26	1.07 (1.02–1.12)	4.97 × 10^−3^	6.44 × 10^−15^
16q12.2	53813367	*FTO*	rs17817449	T/G	0.41	0.91 (0.89–0.95)	2.83 × 10^−7^	0.41	0.95 (0.92–0.99)	0.02	5.26 × 10^−8^
16q12.2	53855291	*FTO*	rs11075995	T/A	0.24	1.11 (1.07–1.15)	3.30 × 10^−8^	0.24	1.01 (0.97–1.06)	0.61	1.56 × 10^−6^
19p13.1	17389704	*MERIT40*	rs8170	G/A	0.19	1.15 (1.11–1.20)	1.35 × 10^−12^	0.19	1.17 (1.11–1.23)	7.29 × 10^−10^	6.64 × 10^−21^
†19p13.1	17393925	*ADHB8*	rs56069439	C/A	0.30	1.16 (1.13–1.20)	8.25 × 10^−19^	0.30	1.19 (1.14–1.24)	1.42 × 10^−15^	1.49 × 10^−32^

CI, confidence interval; EAF, effect allele frequency; ER, oestrogen receptor; GWAS, genome-wide association studies; HR, hazard ratio; OR, odds ratio; SNP, single-nucleotide polymorphism.

^*^P values from iCOGS/BCAC and meta-analysis for ER-negative breast cancer were estimated by *z*-test. *P* values for *BRCA1* carriers were estimated by a kinship-adjusted retrospective likelihood approach.

^†^SNPs with more significant associations with ER-negative disease than known index SNPs from these loci.

**Table 2:** Novel associations of common genetic variants with ER-negative breast cancer risk.

						**iCOGS/GWAS ER-negative**			**BRCA1 carriers**			**Meta-analysis**
**Location**	**Position**	**Nearest gene**	**SNP**	***r***^**2**^	**Allele**	**EAF**	**OR (95% CI)**	P*	**EAF**	**HR (95% CI)**	P*	P*
2p23.2	29119585	*WDR43*	rs67073037	0.98	A/T	0.24	0.92 (0.88–0.95)	3.20 × 10^−6^	0.20	0.92 (0.87–0.96)	4.58 × 10^−4^	4.76 × 10^−9^
2p23.2	29160421	*WDR43*	rs6734079	0.99	T/A	0.23	0.92 (0.88–0.95)	3.99 × 10^−6^	0.20	0.92 (0.87–0.96)	4.55 × 10^−4^	5.50 × 10^−9^
2p23.2	29120733	*WDR43*	rs4577244	1	C/T	0.23	0.92 (0.89–0.95)	6.36 × 10^−6^	0.20	0.92 (0.88–0.96)	5.48 × 10^−4^	1.05 × 10^−8^
												
2q33	201717014	*CLK1*	rs74943274	0.98	G/A	0.015	1.34 (1.18–1.52)	5.89 × 10^−6^	0.02	1.20 (1.03–1.41)	0.012	6.00 × 10^−7^
2q33	201733341	*CLK1/PPIL3*	rs188686860	0.98	C/T	0.016	1.36 (1.20–1.53)	1.16 × 10^−6^	0.02	1.22 (1.04–1.42)	0.012	8.34 × 10^−8^
2q33	201743594	*PPIL3*	rs115635831	1	G/A	0.015	1.36 (1.20–1.54)	1.07 × 10^−6^	0.02	1.21 (1.03–1.41)	0.018	1.26 × 10^−7^
2q33	201935871	*FAM126B/NDUFB3*	rs114962751	1	T/A	0.016	1.36 (1.20–1.53)	1.17 × 10^−6^	0.02	1.22 (1.05–1.42)	0.011	7.24 × 10^−8^
13q22	73957681	*KLF5/KLF12*	rs6562760	1	G/A	0.23	0.92 (0.89–0.96)	1.85 × 10^−5^	0.20	0.89 (0.85–0.94)	2.85 × 10^−6^	4.98 × 10^−10^
13q22	73960952	*KLF5/KLF12*	rs2181965	0.99	G/A	0.23	0.92 (0.89–0.96)	2.16 × 10^−5^	0.20	0.89 (0.85–0.94)	2.39 × 10^−6^	5.04 × 10^−10^
13q22	73964519	*KLF5/KLF12*	rs8002929	1	A/G	0.23	0.93 (0.89–0.96)	2.52 × 10^−5^	0.20	0.89 (0.85–0.94)	1.71 × 10^−6^	5.35 × 10^−10^
												
13q22	73806982	*KLF5/KLF12*	rs12870942	0.99	T/C	0.32	1.09 (1.05–1.13)	2.71 × 10^−7^	0.30	1.06 (1.01–1.10)	0.01	3.75 × 10^−8^
13q22	73811471	*KLF5/KLF12*	rs17181761	0.99	A/C	0.32	1.09 (1.05–1.12)	3.44 × 10^−7^	0.30	1.06 (1.01–1.10)	9.29 × 10^−3^	4.23 × 10^−8^
13q22	73813803	*KLF5/KLF12*	rs9573140	1	A/G	0.32	1.09 (1.05–1.12)	3.77 × 10^−7^	0.30	1.06 (1.01–1.10)	0.01	5.38 × 10^−8^

CI, confidence interval; EAF, Effect allele frequency; ER, oestrogen receptor; GWAS, genome-wide association studies; HR, hazard ratio; OR, odds ratio; *r*^2^, imputation accuracy; SNP, single-nucleotide polymorphism.

^*^P values from iCOGS/BCAC and meta-analysis for ER-negative breast cancer were estimated by *z*-test. *P* values for *BRCA1* carriers were estimated by a kinship-adjusted retrospective likelihood approach.
