# Supplementary material for: Identification of four novel susceptibility loci for oestrogen receptor negative breast cancer
Source: Nat Commun. 2016 Apr 27;7:11375. doi: 10.1038/ncomms11375 (PMC4853421; doi:10.1038/ncomms11375)
Supplement: Supplementary Information — Supplementary Figures 1-12, Supplementary Tables 1-2 and Supplementary Note. [file ncomms11375-s1.pdf]

Supplementary Figure 1

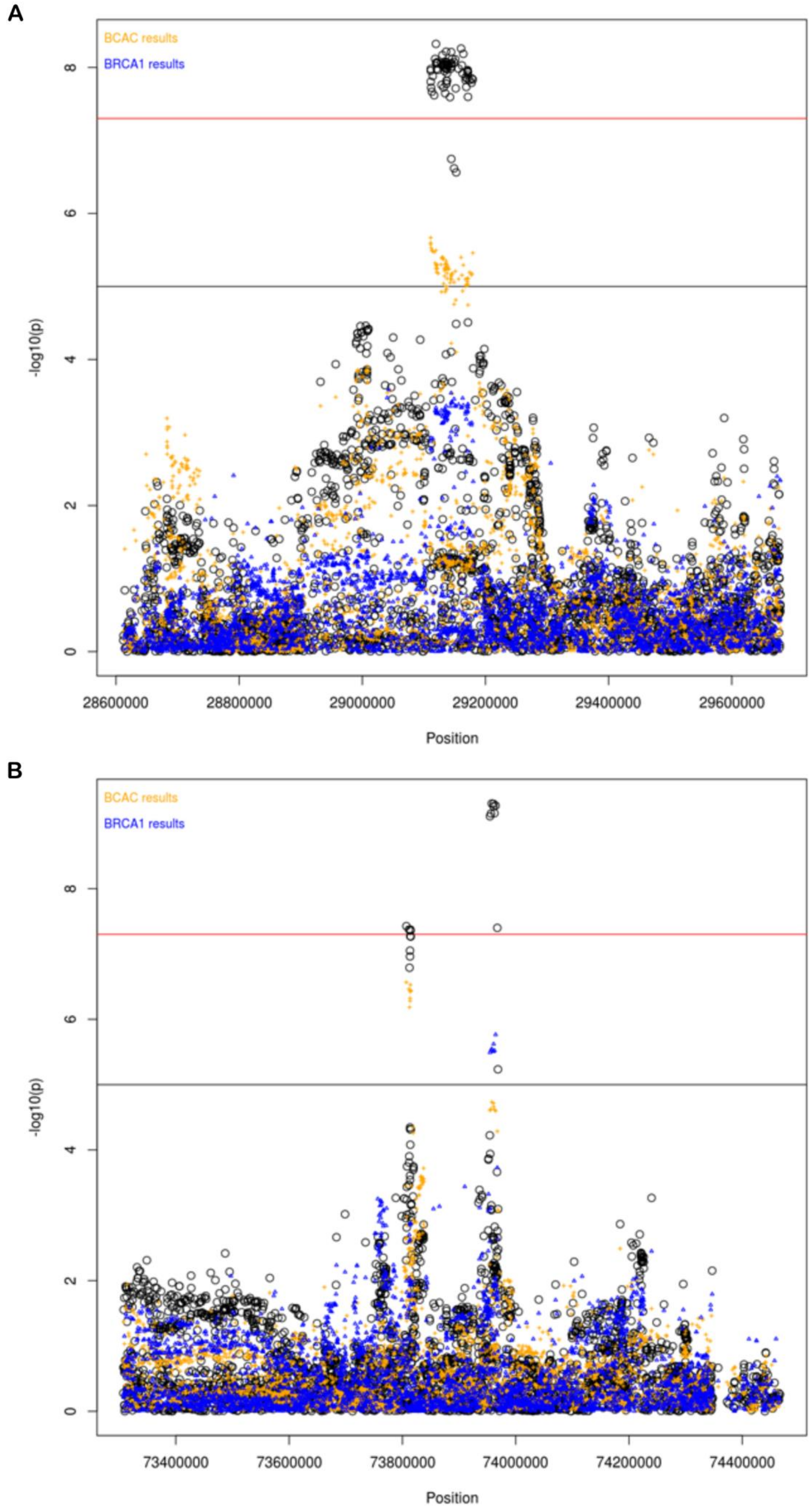

Supplementary Figure 1: The chromosome 2p23.2 and 13q22 regions.

Manhattan Plot for the 2p23.2 (**a**) and 13q22 (**b**) loci displaying the strength of genetic association (-$\log_{10}$ P) versus chromosomal position (Mb), where each dot presents a genotyped or imputed SNP in the meta-analysis (black circle), *BRCA1* breast cancer (blue triangle), or iCOGS/BCAC ER-negative breast cancer (brown). The black horizontal line represents the threshold for significance ($P=1x10^{-5}$) in the iCOGS study and the red horizontal line represents the threshold for significance ($P=5x10^{-8}$) in the meta-analysis.

Supplementary Figure 2

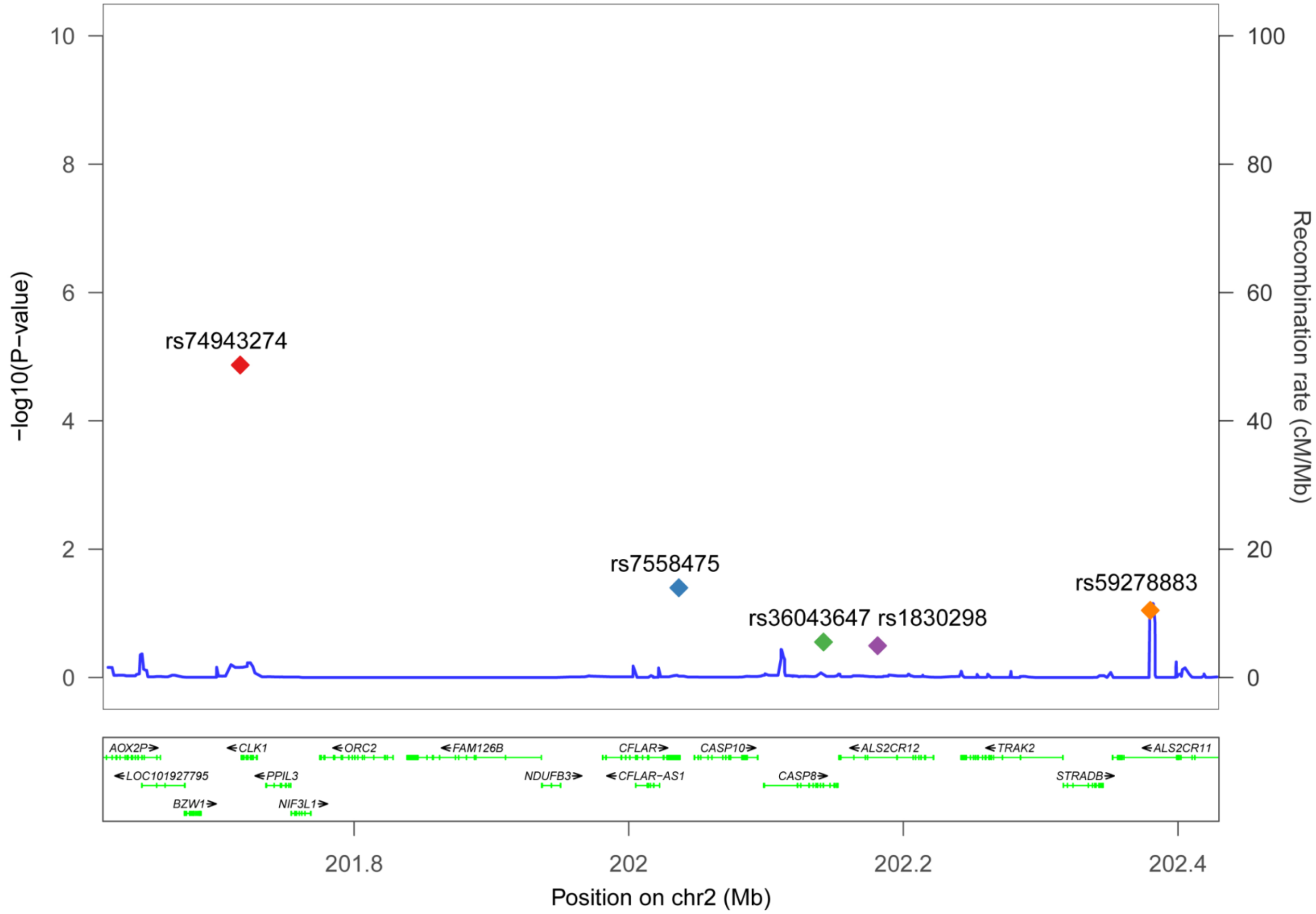

Supplementary Figure 2: Conditional analysis for index SNPs in the *CASP8* region.
The chromosomal position and strength of genetic association (-$\log_{10}$ P) is shown for each of five index SNPs after conditioning for the other index SNPs.

Supplementary Figure 3

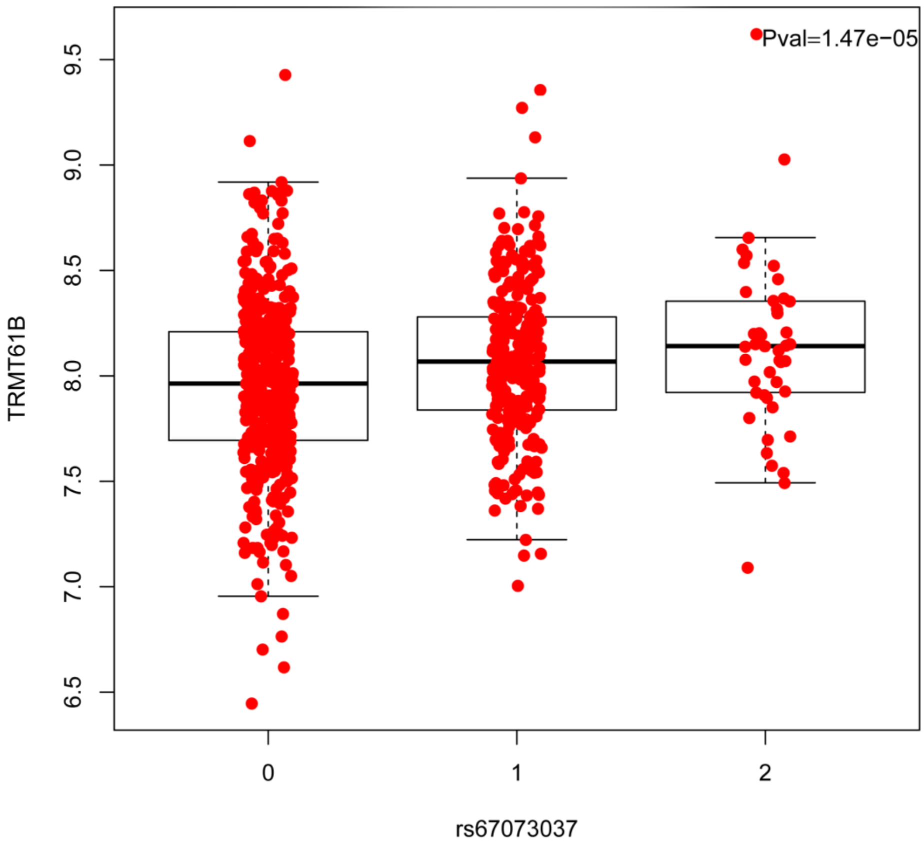

Supplementary Figure 3: Boxplot of eQTL for rs67073037 at 2p23 and *TRMT61B.*
Number of rs67073037 alleles (x-axis) in the iCOGS association study are plotted against TRMT61B expression in the BC765 cohort.

Supplementary Figure 4

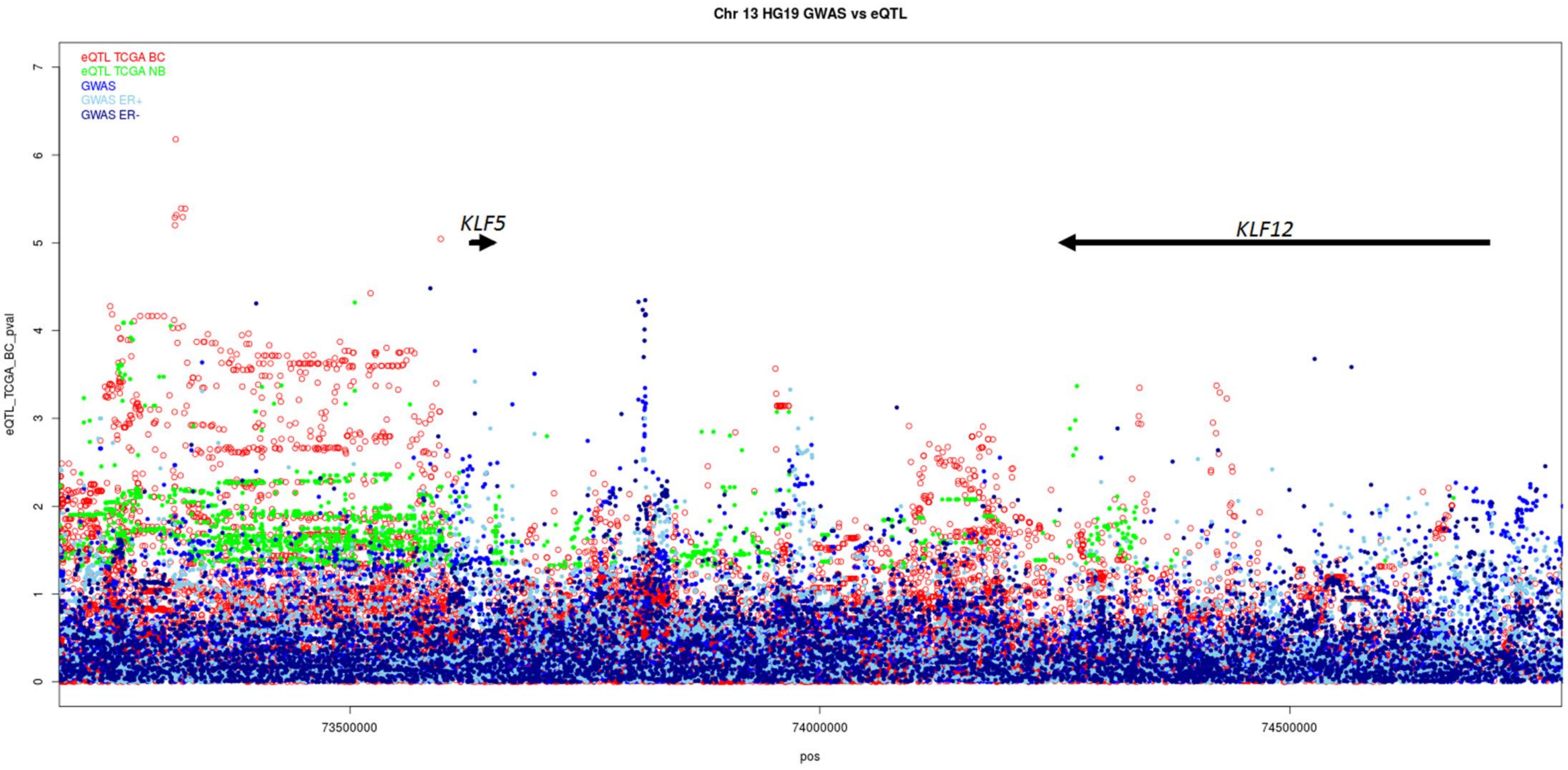

Supplementary Figure 4: cis eQTLs at 13q22.

The chromosomal position of SNPs at 13q22 (GrCh38) are plotted against cis eQTL associations (p-values) in BC765 (eQTL TCGA BC) and 93 normal breast tissues from TCGA, and against significance of associations with overall breast cancer risk in BCAC iCOGS analysis (GWAS), BCAC iCOGS ER-negative breast cancer (GWAS ER-) and BCAC iCOGS ER-positive breast cancer (GWAS ER+).

Supplementary Figure 5

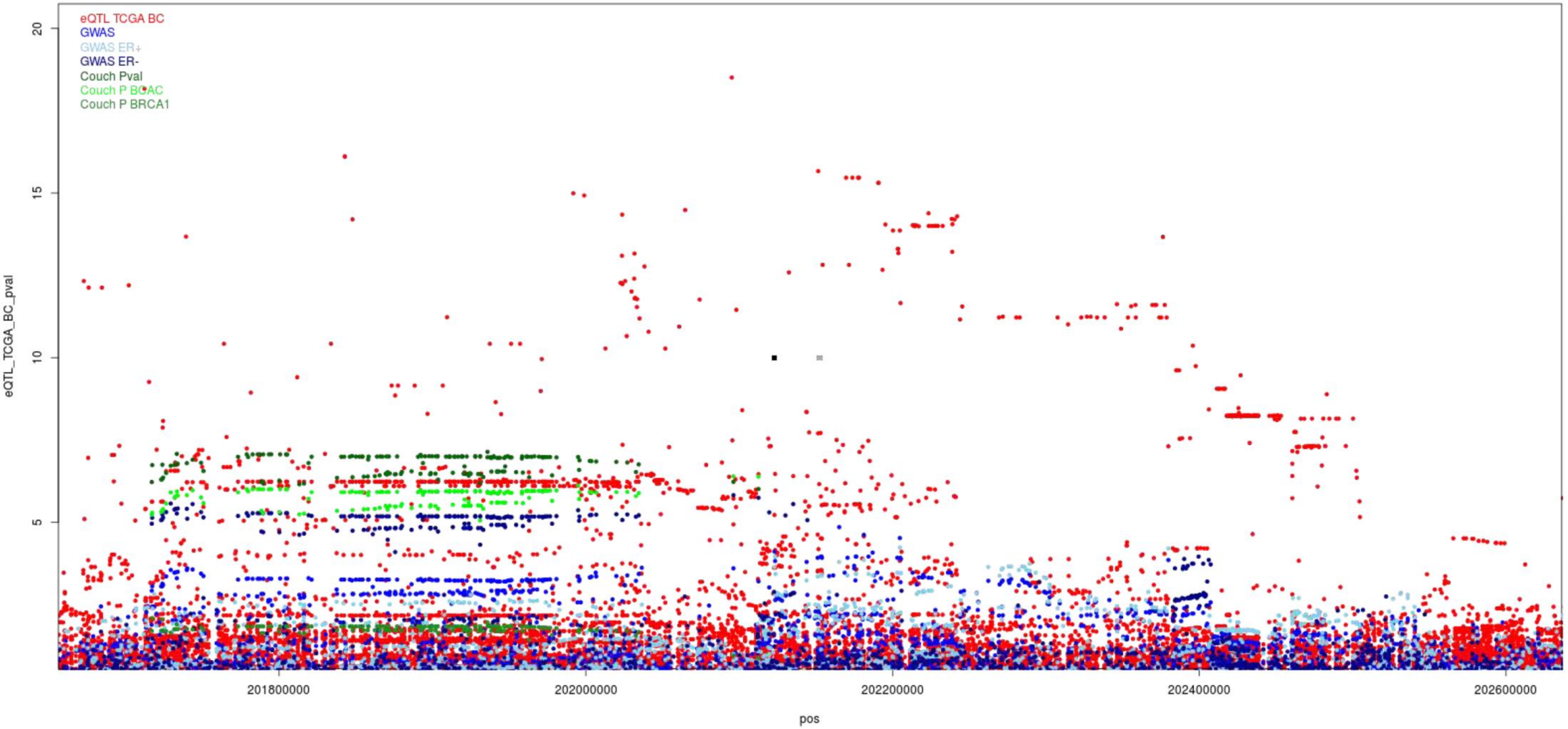

Supplementary Figure 5: cis eQTLs at 2q33.

The chromosomal position of SNPs at 2q33 (GrCh38) are plotted against cis eQTL associations (p-values) in BC765 (eQTL TCGA BC) (red) and against significance of associations with overall breast cancer risk in BCAC iCOGS analysis (GWAS), BCAC iCOGS ER-negative breast cancer (GWAS ER-), BCAC iCOGS ER-positive breast cancer (GWAS ER+), ER-negative breast cancer in the meta-analysis (Couch Pval), ER-negative breast cancer from BCAC in the meta-analysis (Couch P BCAC), and ER-negative breast cancer from BRCA1 in the meta-analysis (Couch P BRCA1).

Supplementary Figure 6

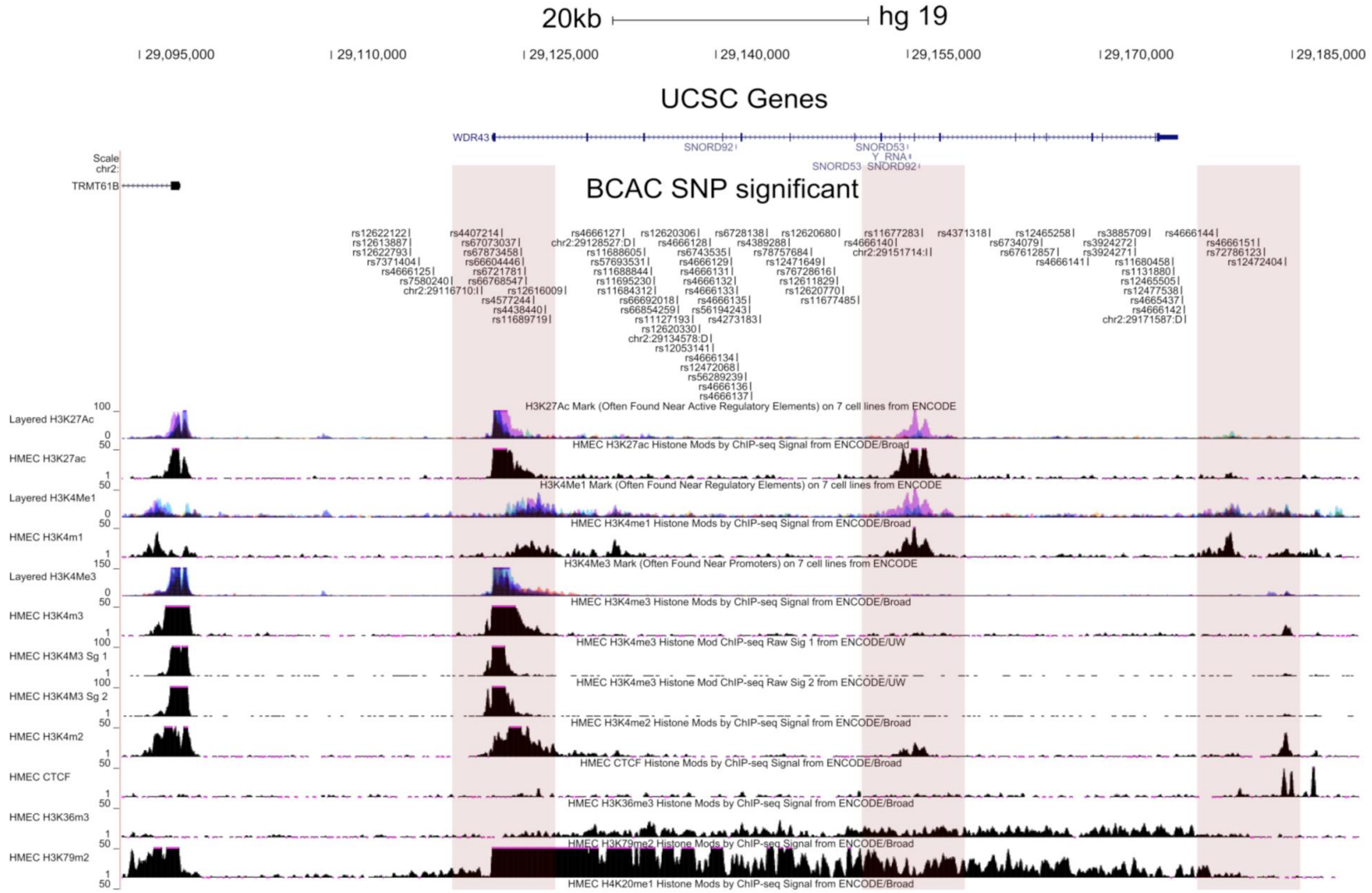

Supplementary Figure 6: Chromatin landscape of 2p23.2.
The locations of the lead SNPs are shown relative to chromosomal position in the region. ChIP-seq analysis of histone marks including acetylated and methylated histone 3 lysine 4 and histone 3 lysine 27 in HMECs are shown as a series of peaks. Three regulatory regions containing SNPs are shaded in pink.

Supplementary Figure 7

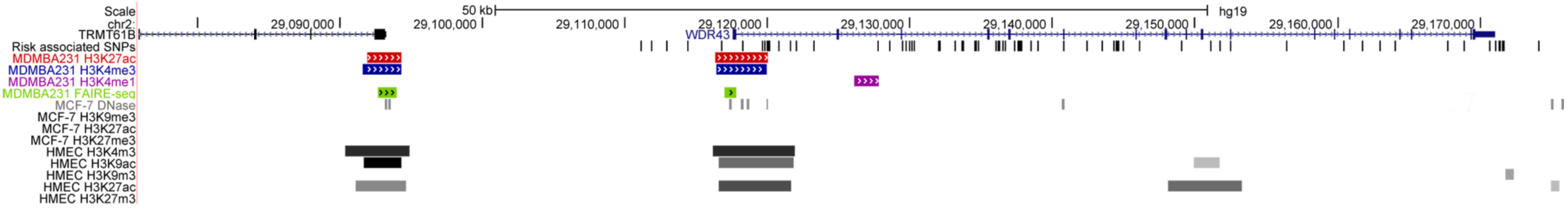

Supplementary Figure 7: Chromatin landscape of 2p23.2 in ER-negative breast cancer.
The locations of the lead SNPs are shown relative to chromosomal position in the region. ChIP-seq analysis of histone marks in MDA-MB-231 ER-negative cells, MCF-7 ER-positive cells, and HMEC are shown as a series of blocks.

Supplementary Figure 8

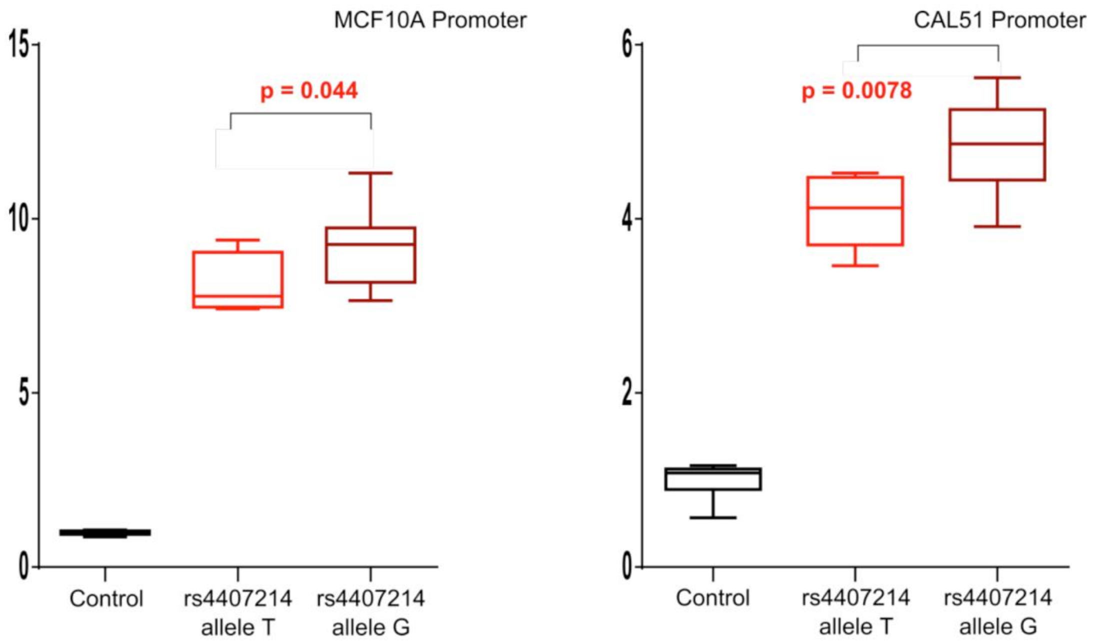

Supplementary Figure 8: Influence of rs4407214 on *WDR43* promoter activity.
Luciferase assays showing activity of *WDR43* promoter in the tile containing SNP rs4407214 T or G alleles in MCF10A and CAL51 cells. The T allele (red box plot) had significantly different activity from the control G allele (brown box) in both MCF10A (P=0.044) and CAL51 (P=0.0078) cells.

Supplementary Figure 9

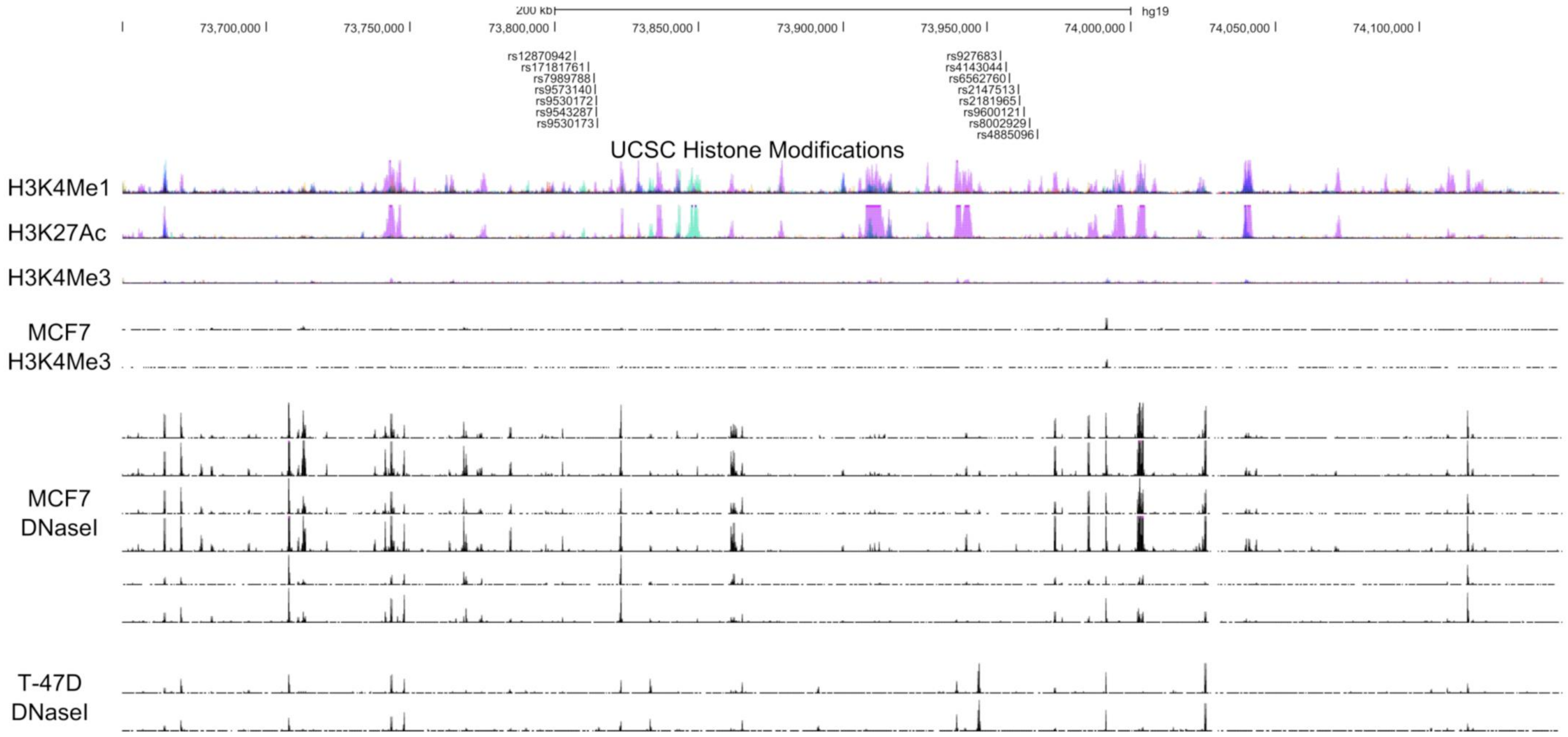

Supplementary Figure 9: Chromatin landscape of 13q22.

The locations of the lead SNPs are shown relative to chromosomal position in the non-genic 13q22 region. ENCODE DNaseI hypersensitive sites derived from MCF7 and T-47D breast cancer cell lines are shown as peaks. ChIP-seq analysis of histone 3 lysine 4 and 27 modifications are shown as a series of peaks.

Supplementary Figure 10

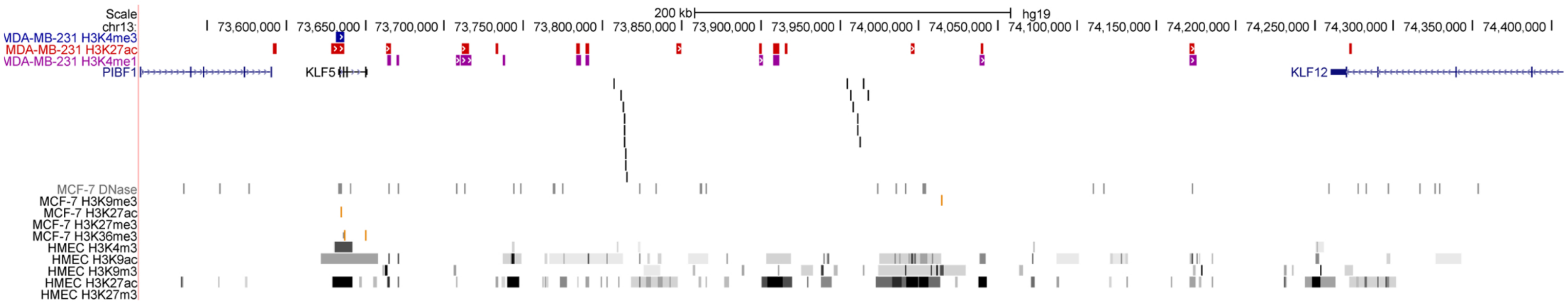

Supplementary Figure 10: Chromatin landscape of 13q22 in ER-negative breast cancer.
The locations of the lead SNPs are shown relative to chromosomal position and histone marks in MDA-MB-231 ER-negative cells, MCF-7 ER-positive cells, and HMECs.

Supplementary Figure 11

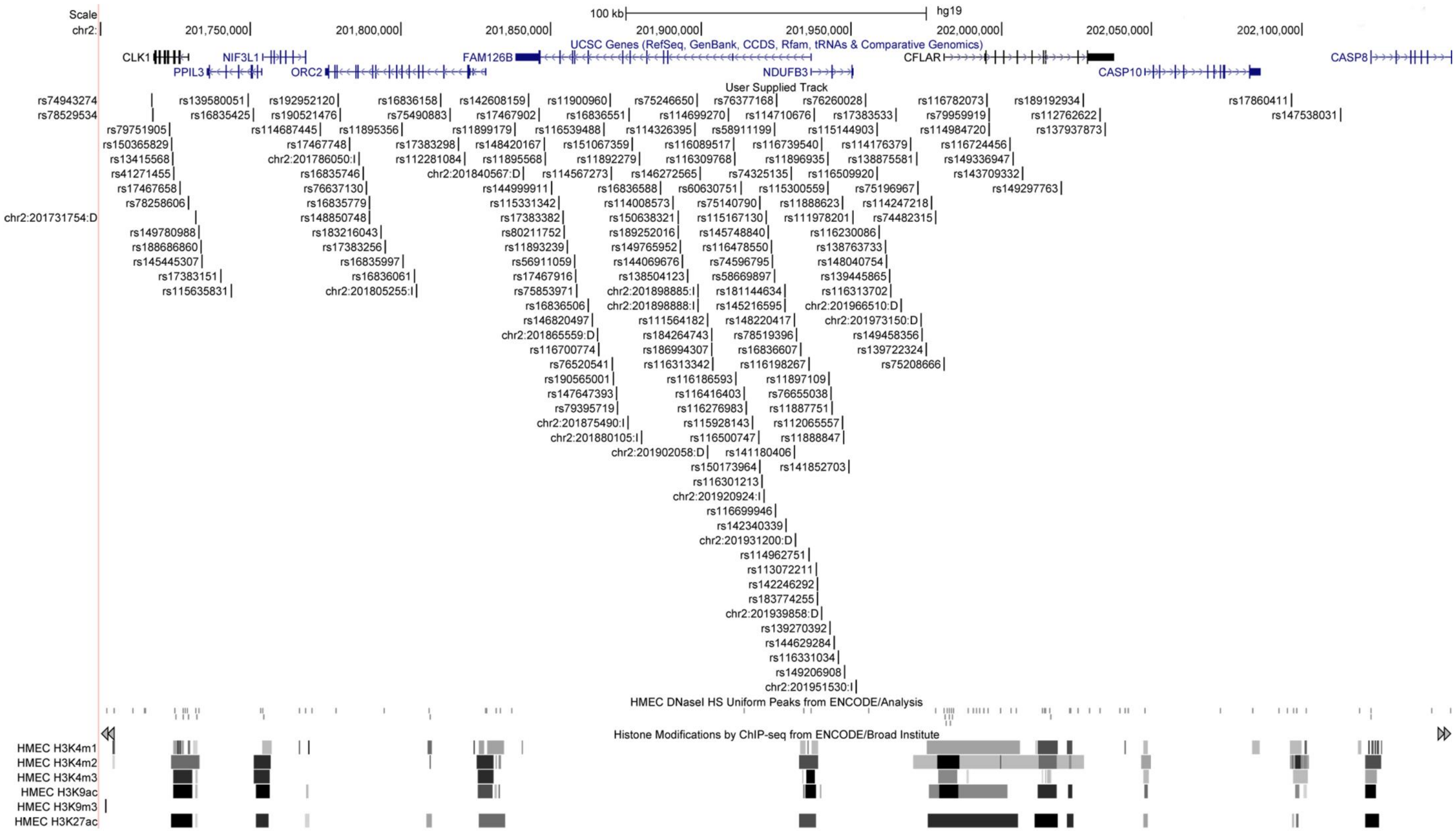

Supplementary Figure 11: Chromatin landscape of 2q33 locus.

The locations of the lead SNPs are shown relative to chromosomal position in the 2q33 region. ENCODE DNaseI hypersensitive sites and histone marks derived from HMEC cells are shown as blocks.

Supplementary Figure 12

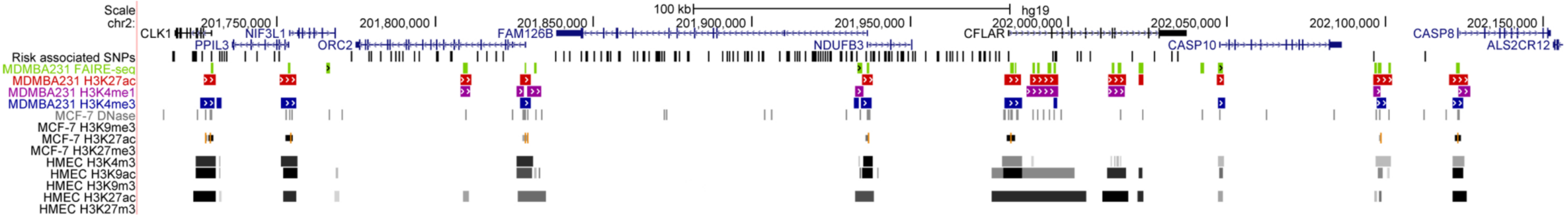

Supplementary Figure 12: Chromatin landscape of 2q33 in ER-negative breast cancer.
The locations of the lead SNPs are shown relative to chromosomal position and histone marks in MDA-MB-231 ER-negative cells, MCF-7 ER-positive cells, and HMECs.

SUPPLEMENTARY MATERIALS

Supplementary Table 1. Samples and genotypes included in the combined analysis

Study	Cases	Controls	Genotyping array	SNPs for MA	SNP inclusion
BRCA1 carriers	7,797	7,455	iCOGS	10,969,793	MAF>0.5%, r^2>0.3
BCAC-iCOGS	7,333	42,468	iCOGS	10,864,787	MAF>0.5%, r^2>0.3
BPC3 GWAS	1,998	2,305	Illumina arrays	9,465,867	MAF varied, r^2>0.3
TNBCC GWAS	1,479	3,180	Illumina arrays	4,835,405	MAF>5%, r^2>0.9
BCAC GWAS	562	6,410	Illumina arrays	10,795,955	MAF>1%, r^2>0.3
EBCG GWAS	900	2,475	Illumina arrays	8,137,183	MAF>1%, r^2>0.3
TOTAL	20,069	64,275		10,909,381	

MA: Meta analysis; MAF: Minor allele frequency

Supplementary Table 2. Genome-wide association studies contributing to BCAC studies

Study[1]	Country	Cases[1]	Controls[1]	ER+/ER- cases[1,2]
**BCAC GWAS**				
ABCFS/kConFab	Australia	282	285	88/72 (72)
BBCS[4]	U.K.	1609	1224	-
DFBBCS[5]	Netherlands	464	3255	-
GC-HBOC[6]	Germany	634	477	-
GWAS_UK2[4]	U.K.	3628	2663	361/160 (160)
HEBCS[7]	Finland	726	1012	522/229 (145)
MARIE[6]	Germany	652	470	567/132 (76)
SASBAC	Sweden	790	756	481/109 (109)
**BPC3 GWAS[3,4]**				
CPS-II	USA	293	295	0/293
EPIC	Europe	511	500	0/511
MEC	USA	86	101	0/86
NHS2	USA	76	374	0/76
PBCS	Poland	543	511	0/543
PLCO	USA	255	340	0/255
NHS	USA	234	184	0/234
CPS-II	USA	293	295	0/293
**EBCG GWAS[5]**				
BCFR (AU)	Australia	593	250	368/176
BCFR (NC)	USA	204	156	130/48
CFR (Ontario)	Canada	668	395	404/185
GESBC	Germany	553	1,071	288/179
LI	US	225	275	112/53
Seattle	US	297	328	219/72
USC	US	983	-	662/198
**TNBCC GWAS[6,7]**				
ABCTB	Australia	144	0	0/144
MCCS	Australia	39	0	0/39
QIMR	Australia	0	650	-
BBCC	Germany	218	0	0/218
GENICA	Germany	59	0	0/59
MARIE	Germany	198	0	0/198
KORA	Germany	0	215	-
DFCI	USA	246	0	0/246
FCCC	USA	120	0	0/120
MCBCS	USA	147	0	0/147
CGEMS	USA	0	947	-
POSH	UK	266	0	0/266
SBCS	UK	42	0	0/42
WTCCC	UK	0	1368	-

[1]For further details see Michailidou 2013[3]
[2]Numbers in brackets are numbers of ER-cases after elimination of duplicates with TNBCC.
[3]For further details see Garcia-Closas et al., 2013[17]
[4]For further details see Siddiq et al., 2012[16]
[5]For further details see Ahsan et al., 2014[35]
[6]For further details see Haiman et al., 2011[14]
[7]For further details see Purrington et al., 2014[19]

Supplementary Table 3: Studies contributing to the BCAC iCOGS analysis

Study[1]	Country	Cases	Controls	ER+/ER-
ABCFS	Australia	643	551	383/204
ABCS	Netherlands	2029	1815	768/282
BBCC	Germany	548	458	460/67
BBCS	U.K.	1507	1397	493/108
BIGGS	Ireland	836	719	495/154
BSUCH	Germany	848	954	548/157
CECILE	France	1019	999	797/144
CGPS	Denmark	2948	4534	1919/357
CNIO-BCS	Spain	902	876	242/88
CTS	U.S.A.	68	71	0/68
DEMOKRITOS	Greece	413	95	0/413
ESTHER	Germany	478	502	304/98
GENICA	Germany	449	427	327/104
HEBCS	Finland	1658	1233	1292/235
HMBCS	Belarus	690	130	37/8
KARBAC	Sweden	722	662	338/63
KBCP	Finland	441	250	300/97
kConFab/AOCS	Australia	575	897	152/55
LMBC	Belgium	2671	1388	2071/378
MARIE	Germany	1743	1788	1328/346
MBCSG	Italy	488	400	149/42
MCBCS	U.S.A.	1836	1931	1486/269
MCCS	Australia	604	511	351/110
MEC	U.S.A.	731	741	415/87
MTLGEBCS	Canada	489	436	421/64
NBCS	Norway	908	217	620/201
NBHS_TN	U.S.A.	125	118	0/125
OBCS	Finland	505	414	405/100
OFBCR	Canada	1175	511	629/269
ORIGO	Netherlands	354	327	208/70
OSU	U.S.A.	207	203	0/207
pKARMA	Sweden	5429	5568	3670/701
RBCS	Netherlands	599	699	323/124
RPCI	U.S.A	136	126	0/136
SASBAC	Sweden	397	661	198/43
SBCS	U.K.	832	848	376/98
SEARCH	U.K.	9293	8068	5146/1173
SKKDKFZS	Germany	135	168	0/135
SZBCS	Poland	365	315	165/60
UKBGS	U.K.	470	470	95/22

[1]For further details see [3]

Supplementary Table 4. CIMBA iCOGS studies with *BRCA1* carriers of European ancestry affected or unaffected with breast cancer

Study	Study location	Unaffected	Affected
BCFR	Canada/Australia	177	286
BFBOCC	Latvia/Lithuania	88	58
BIDMC	USA	18	27
BMSA	South Africa	23	38
BRICOH	USA	114	62
CBCS	Denmark	175	121
CNIO	Spain/Greece	127	182
COH	USA	86	139
CONSIT TEAM	Italy	436	487
DFCI	USA	79	77
DKFZ	Germany	79	77
DNA HEBON	Netherlands	692	564
EMBRACE	UK	729	725
FCCC	USA	68	47
GC-HBOC	Germany	334	645
GEMO	France/USA	576	791
GEORGETOWN	USA	6	10
G-FaST	Belgium	109	115
GOG	USA	172	172
HCSC	Spain	75	56
HEBCS	Finland	42	55
HUNBOCS	Hungary	60	83
HVH	Spain	25	33
ICO	Spain	114	100
IHCC	Poland	392	311
INHERIT	Canada	54	42
IOVHBOCS	Italy	59	74
IPOBCS	Portugal	60	35
KCONFAB	Australia	313	418
MAGIC	USA	30	17
MAGIC-UC	USA	21	16
MAYO	USA	138	138
MCGILL	Canada	30	24
MDAND	USA	44	71
MODSQUAD	Czech Republic/Belgium	79	141
MSKCC	USA	137	201
MUV	Austria	222	224
NAROD	Canada	100	46
NCI	USA	113	45
NNPIO	Russia	17	34
OCGN	Canada	139	75
OSU CCG	USA	33	54
OUH	Denmark	226	141
PBCS	Italy	35	45
SMC	Israel	156	107
SWE-BRCA	Sweden	266	220

UCSF	USA	59	44
UKGRFOCR	UK	42	18
UPENN	USA	174	242
VFCTG	Australia	15	44
WCRI	USA	158	55

Supplementary Table 5. Associations with ER-negative breast cancer for SNPs most significantly associated with overall breast cancer from known breast cancer risk loci

				iCOGS/GWAS ER-negative			*BRCA1* carriers			Meta
**Chr**	**Position**	**rs#**	**Alleles**	**MAF**	**OR**	**P-value[6]**	**MAF**	**HR**	**P-value[5]**	**P-value[3]**
1	10566215	rs616488	A/G	0.33	0.91 (0.87-0.96)	$1.51x10^{-7}$	0.32	0.96 (0.92-1.01)	0.092	$2.26x10^{-7}$
1	114448389	rs11552449	C/T	0.17	1.06 (1.02-1.11)	$6.01x10^{-3}$	0.16	1.03 (0.98-1.09)	0.23	$3.80x10^{-3}$
1	121280613	rs11249433	A/G	0.40	1.00 (0.97-1.03)	0.93	0.41	0.99 (0.96-1.04)	0.83	0.95
1	145644984	rs12405132	C/T	0.37	0.97 (0.94-1.00)	0.082	0.35	1.01 (0.97-1.06)	0.57	0.31
1	149927034	rs12048493	A/C	0.33	1.03 (0.99-1.07)	0.11	0.33	1.06 (1.01-1.11)	0.02	$6.10x10^{-3}$
1	202187176	rs6678914	G/A	0.41	0.91 (0.88-0.94)	$3.73x10^{-9}$	0.40	0.98 (0.94-1.02)	0.42	$3.11x10^{-7}$
1	204518842	rs4245739	A/C	0.26	1.13 (1.10-1.16)	$5.53x10^{-15}$	0.28	1.09 (1.04-1.13)	$6.83x10^{-5}$	$7.71x10^{-18}$
1	242034263	rs72755295	A/G	0.03	1.13 (1.04-1.21)	$3.91x10^{-3}$	0.03	1.09 (0.98-1.22)	0.11	$1.30x10^{-3}$
2	19184284	rs12710696	C/T	0.36	1.10 (1.06-1.13)	$1.66x10^{-8}$	0.39	1.01 (0.97-1.05)	0.56	$1.91x10^{-6}$
2	121245122	rs4849887	C/T	0.10	0.92 (0.87-0.97)	$1.87x10^{-3}$	0.11	1.02 (0.96-1.09)	0.58	0.043
2	172972971	rs2016394	G/A	0.48	1.02 (0.98-1.05)	0.34	0.47	1.01 (0.97-1.06)	0.48	0.24
2	174212894	rs1550623	A/G	0.16	0.96 (0.92-1.01)	0.093	0.15	1.01 (0.95-1.04)	0.72	0.27
2	202149589	rs1045485	G/C	0.13	0.94 (0.90-0.98)	$8.46x10^{-3}$	0.12	0.94 (0.89-1.00)	0.052	$1.09x10^{-3}$
2	217905832	rs13387042	A/G	0.49	0.95 (0.91-0.98)	$1.24x10^{-3}$	0.47	0.98 (0.95-1.02)	0.40	$2.26x10^{-3}$
2	218296508	rs16857609	C/T	0.26	1.08 (1.04-1.12)	$2.46x10^{-5}$	0.26	1.04 (1.00-1.09)	0.058	$6.95x10^{-6}$
3	4742276	rs6762644	A/G	0.40	1.01 (0.97-1.04)	0.69	0.36	1.03 (0.99-1.08)	0.11	0.19
3	27416013	rs4973768	C/T	0.47	1.04 (1.01-1.07)	0.013	0.49	1.01 (0.98-1.06)	0.48	0.017
3	30682939	rs12493607	G/C	0.35	1.01 (0.98-1.04)	0.65	0.35	0.99 (0.95-1.03)	0.72	0.90
3	46866866	rs6796502	G/A	0.10	0.95 (0.90-1.01)	0.08	0.10	1.01 (0.94-1.08)	0.80	0.23
3	63967900	rs1053338	A/G	0.13	1.04 (0.99-1.08)	0.094	0.14	0.99 (0.94-1.05)	0.77	0.27
4	106084778	rs9790517	C/T	0.22	1.04(1.01-1.08)	0.021	0.23	0.98 (0.94-1.03)	0.52	0.16
4	175846426	rs6828523	C/A	0.12	0.99 (0.95-1.04)	0.76	0.11	1.02 (0.96-1.09)	0.45	0.82
5	1279790	rs10069690	C/T	0.26	1.15 (1.11-1.20)	$5.84x10^{-13}$	0.28	1.20 (1.15-1.25)	$9.17x10^{-16}$	$8.70x10^{-27}$
5	1282319	rs7726159	C/A	0.34	1.09 (1.05-1.13)	$2.19x10^{-6}$	0.35	1.07 (1.02-1.11)	$1.8x10^{-3}$	$3.31x10^{-8}$
5	1297488	rs2736108	C/T	0.29	0.89 (0.86-0.93)	$1.41x10^{-8}$	0.29	0.89 (0.86-0.93)	$4.05x10^{-7}$	$3.05x10^{-14}$
5	16187528	rs13162653	G/T	0.45	0.95 (0.92-0.99)	$7.46x10^{-3}$	0.44	0.97 (0.93-1.02)	0.21	$4.22x10^{-3}$
5	32567732	rs2012709	C/T	0.47	1.02 (0.99-1.06)	0.19	0.49	0.99 (0.96-1.03)	0.78	0.40
5	44706498	rs10941679	A/G	0.25	1.04 1.00-1.07)	0.059	0.25	0.99 (0.95-1.04)	0.66	0.25
5	56031884	rs889312	A/C	0.28	1.05 (1.02-1.09)	$1.88x10^{-3}$	0.29	1.01 (0.97-1.06)	0.52	$4.63x10^{-3}$
5	58184061	rs10472076	T/C	0.37	1.05 (1.02-1.08)	$3.06x10^{-3}$	0.37	1.01 (0.97-1.05)	0.78	0.013
5	58337481	rs1353747	T/G	0.10	0.92 (0.86-0.98)	$7.37x10^{-3}$	0.09	0.98 (0.91-1.05)	0.56	0.014
5	81538046	rs7707921	A/T	0.24	0.97 (0.93-1.01)	0.097	0.25	1.03 (0.98-1.08)	0.21	0.60

5	158244083	rs1432679	T/C	0.44	1.07 (1.04-1.10)	$1.16x10^{-5}$	0.44	1.04 (1.00-1.08)	0.035	$2.14x10^{-6}$
6	1318878	rs11242675	T/C	0.38	0.96 (0.91-0.98)	$1.09x10^{-3}$	0.35	0.96 (0.91-1.00)	0.044	$1.34x10^{-4}$
6	13722523	rs204247	A/G	0.44	1.03 (0.99-1.06)	0.099	0.44	1.00 (0.96-1.04)	0.98	0.20
6	28926220	rs150750171	G/C	0.38	1.06 (1.02-1.10)	$1.51x10^{-3}$	0.41	1.03 (0.99-1.08)	0.11	$5.94x10^{-4}$
6	82128386	rs17529111	T/C	0.22	1.07 (1.03-1.11)	$2.47x10^{-4}$	0.22	1.02 (0.97-1.07)	0.39	$7.83x10^{-4}$
6	151918856	rs12662670	T/G	0.08	1.20 (1.15-1.24)	$8.90x10^{-15}$	0.09	1.19 (1.11-1.27)	$9.67x10^{-7}$	$1.32x10^{-19}$
6	151948366	rs2046210	G/A	0.34	1.15 (1.12-1.19)	$1.36x10^{-17}$	0.37	1.15 (1.11-1.20)	$6.32x10^{-12}$	$5.90x10^{-28}$
7	91630620	rs6964587	G/T	0.39	1.03 (0.99-1.06)	0.099	0.41	1.00 (0.96-1.04)	0.96	0.21
7	130667121	rs4593472	C/T	0.35	0.98 (0.95-1.02)	0.36	0.34	0.97 (0.93-1.01)	0.17	0.11
7	144074929	rs720475	G/A	0.25	1.00 (0.97-1.04)	0.89	0.26	0.98 (0.94-1.02)	0.36	0.64
8	29509616	rs9693444	C/A	0.32	1.06 (1.03-1.10)	$2.19x10^{-4}$	0.33	1.01 (0.96-1.05)	0.75	$1.97x10^{-3}$
8	36858483	rs13365225	A/G	0.17	0.90 (0.85-0.95)	$1.02x10^{-5}$	0.19	0.96 (0.91-1.01)	0.085	$7.47x10^{-6}$
8	76230301	rs6472903	T/G	0.18	0.94 (0.90-0.99)	$9.79x10^{-3}$	0.16	1.01 (0.96-1.06)	0.71	0.072
8	76417937	rs2943559	A/G	0.07	1.09 (1.04-1.14)	$1.44x10^{-3}$	0.08	1.06 (0.99-1.14)	0.11	$4.91x10^{-4}$
8	117209548	rs13267382	G/A	0.35	1.05 (1.02-1.09)	$4.83x10^{-3}$	0.36	0.99 (0.94-1.03)	0.52	0.082
8	128355618	rs13281615	A/G	0.40	1.03 (1.00-1.06)	0.038	0.43	1.01 (0.97-1.06)	0.51	0.042
8	129194641	rs11780156	C/T	0.16	1.05 (1.01-1.09)	0.026	0.19	0.95 (0.91-1.00)	0.063	0.60
9	22062134	rs1011970	G/T	0.17	1.11 (1.06-1.15)	$1.55x10^{-6}$	0.17	1.02 (0.97-1.07)	0.51	$2.71x10^{-5}$
9	110306115	rs10759243	C/A	0.29	1.02 (0.99-1.06)	0.22	0.31	0.98 (0.94-1.03)	0.42	0.69
9	110888478	rs865686	T/G	0.38	0.98 (0.95-1.01)	0.24	0.36	0.99 (0.95-1.03)	0.75	0.26
10	5886734	rs2380205	C/T	0.44	1.00 (0.97-1.03)	0.97	0.43	1.02 (0.98-1.06)	0.42	0.59
10	22032942	rs7072776	G/A	0.28	0.98 (0.95-1.02)	0.29	0.31	0.99 (0.94-1.03)	0.55	0.23
10	22315843	rs11814448	A/C	0.02	1.18 (1.08-1.27)	$1.03x10^{-3}$	0.02	1.13 (0.99-1.30)	0.07	$2.43x10^{-4}$
10	64278682	rs10995190	G/A	0.16	0.87 (0.83-0.91)	$3.75x10^{-8}$	0.16	0.99 (0.94-1.04)	0.70	$8.23x10^{-6}$
10	80841148	rs704010	C/T	0.38	1.04 (1.01-1.07)	0.016	0.37	1.01 (0.97-1.05)	0.48	0.02
10	114773927	rs7904519	A/G	0.46	1.06 (1.03-1.09)	$8.00x10^{-5}$	0.47	1.09 (1.05-1.14)	$1.54x10^{-5}$	$7.58x10^{-9}$
10	123093901	rs11199914	C/T	0.32	1.00 (0.97-1.04)	0.83	0.33	1.02 (0.98-1.07)	0.27	0.39
10	123337335	rs2981579	G/A	0.41	1.03 (0.99-1.06)	0.12	0.42	0.99 (0.95-1.03)	0.80	0.29
11	1909006	rs3817198	T/C	0.31	1.05 (1.02-1.09)	$1.34x10^{-3}$	0.33	1.07 (1.03-1.12)	$1.09x10^{-3}$	$5.45x10^{-6}$
11	65583066	rs3903072	G/T	0.47	0.96 (0.93-0.99)	$8.59x10^{-3}$	0.48	0.99 (0.95-1.03)	0.67	0.020
11	69331418	rs78540526	C/T	0.08	1.02 (0.96-1.09)	0.44	0.08	1.03 (0.96-1.11)	0.42	0.27
11	69331642	rs554219	C/G	0.12	1.03 (0.98-1.07)	0.29	0.12	1.03 (0.97-1.09)	0.38	0.17
11	69379161	rs75915166	C/A	0.06	1.04 (0.97-1.12)	0.28	0.06	1.03 (0.95-1.12)	0.49	0.20
11	129461171	rs11820646	C/T	0.41	0.95 (0.92-0.98)	$3.09x10^{-3}$	0.38	0.93 (0.88-0.97)	$5.8x10^{-4}$	$8.75x10^{-6}$
12	14413931	rs12422552	G/C	0.26	1.06 (1.02-1.10)	$1.61x10^{-3}$	0.27	1.01 (0.97-1.06)	0.66	$6.25x10^{-3}$
12	28155080	rs10771399	A/G	0.12	0.79 (0.72-0.85)	$3.82x10^{-13}$	0.10	0.86 (0.80-0.91)	$2.55x10^{-6}$	$7.18x10^{-18}$

12	96027759	rs17356907	A/G	0.30	0.91 (0.88-0.59)	$2.94x10^{-6}$	0.29	0.95 (0.91-1.00)	0.03	$5.42x10^{-7}$
12	115836522	rs1292011	A/G	0.42	0.99 (0.96-1.02)	0.45	0.41	1.01 (0.97-1.05)	0.79	0.67
13	32972626	rs11571833	A/T	0.01	1.32 (1.20-1.43)	$5.84x10^{-6}$	0.01	1.04 (0.84-1.28)	0.73	$1.84x10^{-4}$
14	37132769	rs2236007	G/A	0.21	0.97 (0.93-1.01)	0.13	0.21	0.97 (0.92-1.02)	0.21	0.048
14	68660428	rs2588809	C/T	0.16	0.99 (0.95-1.03)	0.67	0.19	0.96 (0.90-1.01)	0.10	0.17
14	69034682	rs999737	C/T	0.23	0.94 (0.9-0.97)	$5.11x10^{-4}$	0.21	0.96 (0.92-1.01)	0.10	$1.71x10^{-4}$
14	91841069	rs941764	A/G	0.34	1.02 (0.99-1.05)	0.24	0.34	1.02 (0.98-1.07)	0.25	0.10
14	93104072	rs11627032	T/C	0.27	0.91 (0.87-0.95)	$1.24x10^{-5}$	0.28	0.97 (0.92-1.01)	0.15	$1.64x10^{-5}$
16	52586341	rs3803662	G/A	0.27	1.13 (1.10-1.17)	$6.01x10^{-13}$	0.29	1.05 (1.01-1.09)	0.018	$1.01x10^{-12}$
16	53813367	rs17817449	T/G	0.41	0.91 (0.88-0.95)	$2.83x10^{-7}$	0.41	0.95 (0.92-0.99)	0.022	$5.26x10^{-8}$
16	53855291	rs11075995	T/A	0.24	1.10 (1.06-1.14)	$3.30x10^{-8}$	0.24	1.01 (0.97-1.06)	0.61	$1.56x10^{-6}$
16	80650805	rs13329835	A/G	0.22	1.02 (0.98-1.06)	0.26	0.23	1.04 (0.99-1.09)	0.089	0.053
17	29230520	chr17:29230520:D	GGT/G	0.21	0.94 (0.90-0.98)	$5.39x10^{-3}$	0.22	1.00 (0.95-1.05)	0.96	0.033
17	53056471	rs6504950	G/A	0.28	0.97 (0.93-1.00)	0.05	0.27	0.98 (0.94-1.03)	0.48	0.05
17	77781725	rs745570	A/G	0.50	0.93 (0.90-0.96)	$4.15x10^{-5}$	0.49	0.96 (0.92-1.00)	0.036	$6.26x10^{-6}$
18	24337424	rs527616	G/C	0.38	0.97 (0.94-1.00)	0.075	0.37	0.99 (0.95-1.03)	0.62	0.094
18	24570667	rs1436904	T/G	0.40	1.00 (0.97-1.03)	0.88	0.39	0.99 (0.95-1.03)	0.63	0.86
18	42399590	rs6507583	A/G	0.07	0.97 (0.90-1.03)	0.32	0.07	0.98 (0.91-1.07)	0.70	0.31
19	17389704	rs8170	G/A	0.19	1.15 (1.11-1.20)	$1.35x10^{-12}$	0.19	1.17 (1.11-1.23)	$7.29x10^{-10}$	$6.64x10^{-21}$
19	17394124	rs2363956	G/T	0.49	1.13 (1.09-1.16)	$1.33x10^{-13}$	0.50	1.15 (1.11-1.18)	$3.79x10^{-15}$	$1.29x10^{-26}$
19	18571141	rs4808801	A/G	0.35	0.93 (0.90-0.97)	$9.44x10^{-5}$	0.32	0.98 (0.94-1.02)	0.40	$3.32x10^{-4}$
19	44286513	rs3760982	G/A	0.46	1.03 (1.00-1.06)	0.082	0.46	1.03 (0.99-1.07)	0.12	0.019
21	16520832	rs2823093	G/A	0.27	0.99 (0.95-1.02)	0.45	0.27	0.95 (0.91-1.00)	0.039	0.061
22	29121087	rs17879961	A/G	0.03	1.06 (0.61-1.37)	0.75	0.01	0.83 (0.61-1.15)	0.26	0.75
22	29621477	rs132390	T/C	0.04	1.08 (0.99-1.16)	0.084	0.03	1.02 (0.91-1.14)	0.74	0.27
22	40876234	rs6001930	T/C	0.11	1.10 (1.06-1.15)	$1.03x10^{-5}$	0.11	1.07 (1.01-1.14)	0.034	$1.65x10^{-6}$

[1] Number of women unaffected by cancer in the analysis of breast cancer associations
[2] Number of women affected with breast cancer in the analysis of breast cancer associations
[3] P-values (chi-square) from the meta-analysis of the associations between SNP and breast cancer in *BRCA1* and *BRCA2* carriers and ER-negative breast cancer in case-control studies
[4] Effect allele frequency
[5] P-values for BRCA1 carriers were estimated by a kinship-adjusted retrospective likelihood approach.
[6] P-values for iCOGS/GWAS were estimated by Chi-square

Supplementary Table 6. Association studies for Asian breast cancer risk loci and ER-negative breast cancer

				Caucasian							Asian						African American		
				iCOGS/GWAS			BRCA1 Carriers			Meta-analysis	iCOGS Overall			iCOGS ER-negative			iCOGS Overall Risk		
**Chr**	**Position**	**SNP**	**Alleles**	**EAF**	**OR**	**P**	**EAF**	**HR**	**P**	**P**	**EAF**	**OR**	**P**	**EAF**	**OR**	**P**	**EAF**	**OR**	**P**
1	203766331	rs4951011	A/G	0.15	0.97	0.13	0.13	0.97	0.15	0.06	0.31	1.03	0.36	0.31	1.04	0.40	0.05	1.14	0.36
5	90732225	rs10474352	C/T	0.15	0.99	0.71	0.15	0.97	0.36	0.40	0.44	0.97	0.36	0.44	0.98	0.68	0.36	1.04	0.58
15	91512067	rs2290203	G/A	0.20	0.96	0.03	0.21	0.98	0.36	0.02	0.49	0.92	$1.1x10^{-3}$	0.49	0.96	0.31	0.41	0.98	0.76

P-values for BRCA1 carriers were estimated by a kinship-adjusted retrospective likelihood approach.
P-values for iCOGS/GWAS and for meta-analysis were estimated by Chi-square

Supplementary Table 7. ER-negative breast cancer association tests in BCAC, *BRCA1* carriers, and in a meta-analysis for 2p23.2 SNPs

					iCOGS/BCAC			BRCA1			Meta-analysis
**Chr**	**Position**	**SNP**	**A1**	**A2**	**EAF**	**OR**	**P**	**EAF**	**HR**	**P**	**P**
2	29119585	rs67073037	A	T	0.24	0.91	$3.20x10^{-6}$	0.22	0.92	$4.58x10^{-4}$	$4.76x10^{-9}$
2	29160421	rs6734079	T	A	0.23	0.92	$3.99x10^{-6}$	0.21	0.92	$4.55x10^{-4}$	$5.50x10^{-9}$
2	29136136	rs4666129	A	T	0.23	0.91	$4.46x10^{-6}$	0.21	0.92	$4.35x10^{-4}$	$6.08x10^{-9}$
2	29144234	rs76728616	G	A	0.23	0.92	$7.35x10^{-6}$	0.21	0.91	$2.87x10^{-4}$	$6.14x10^{-9}$
2	29164022	rs4666141	G	T	0.23	0.92	$5.09x10^{-6}$	0.22	0.92	$4.23x10^{-4}$	$6.51x10^{-9}$
2	29130140	rs11695230	G	C	0.23	0.92	$3.94x10^{-6}$	0.22	0.92	$6.02x10^{-4}$	$6.75x10^{-9}$
2	29146094	rs11677485	T	C	0.23	0.91	$6.59x10^{-6}$	0.21	0.92	$3.85x10^{-4}$	$7.59x10^{-9}$
2	29118258	rs4407214	T	G	0.23	0.91	$4.79x10^{-6}$	0.21	0.92	$5.04x10^{-4}$	$7.63x10^{-9}$
2	29151035	rs11677283	C	T	0.23	0.92	$6.18x10^{-6}$	0.21	0.92	$4.26x10^{-4}$	$7.74x10^{-9}$
2	29134743	rs12053141	A	G	0.23	0.91	$5.63x10^{-6}$	0.21	0.92	$4.88x10^{-4}$	$8.29x10^{-9}$
2	29149051	rs4666140	C	T	0.22	0.92	$8.76x10^{-6}$	0.21	0.91	$3.48x10^{-4}$	$8.44x10^{-9}$
2	29121611	rs4438440	G	C	0.23	0.92	$5.04x10^{-6}$	0.21	0.92	$5.46x10^{-4}$	$8.45x10^{-9}$
2	29136272	rs4666131	T	C	0.23	0.91	$5.63x10^{-6}$	0.21	0.92	$5.15x10^{-4}$	$8.48x10^{-9}$
2	29137567	rs4666135	C	T	0.23	0.92	$5.54x10^{-6}$	0.21	0.92	$5.35x10^{-4}$	$8.62x10^{-9}$
2	29120116	rs66604446	A	G	0.23	0.91	$5.13x10^{-6}$	0.21	0.92	$5.44x10^{-4}$	$8.63x10^{-9}$
2	29119930	rs66768547	C	T	0.23	0.92	$5.16x10^{-6}$	0.21	0.92	$5.44x10^{-4}$	$8.63x10^{-9}$
2	29144611	rs12620680	C	T	0.23	0.92	$6.87x10^{-6}$	0.21	0.92	$3.91x10^{-4}$	$8.71x10^{-9}$
2	29120030	rs67873458	G	A	0.23	0.92	$5.65x10^{-6}$	0.21	0.92	$5.17x10^{-4}$	$8.74x10^{-9}$
2	29119773	rs6721781	T	C	0.23	0.91	$5.62x10^{-6}$	0.21	0.92	$5.17x10^{-4}$	$8.74x10^{-9}$
2	29122001	rs11689719	G	T	0.23	0.92	$5.67x10^{-6}$	0.21	0.92	$5.19x10^{-4}$	$8.77x10^{-9}$
2	29127745	rs4666127	C	T	0.23	0.92	$5.01x10^{-6}$	0.21	0.92	$5.68x10^{-4}$	$8.78x10^{-9}$
2	29144456	rs4666132	T	C	0.23	0.91	$6.98x10^{-6}$	0.21	0.92	$3.91x10^{-4}$	$8.94x10^{-9}$
2	29136489	rs12611829	G	T	0.23	0.92	$5.62x10^{-6}$	0.21	0.92	$5.44x10^{-4}$	$8.94x10^{-9}$
2	29137309	rs56289239	C	T	0.23	0.92	$5.65x10^{-6}$	0.21	0.92	$5.38x10^{-4}$	$9.07x10^{-9}$
2	29138978	rs11127193	C	T	0.23	0.92	$5.68x10^{-6}$	0.21	0.92	$5.38x10^{-4}$	$9.08x10^{-9}$
2	29133121	rs6728138	G	T	0.23	0.92	$5.68x10^{-6}$	0.21	0.92	$5.39x10^{-4}$	$9.08x10^{-9}$
2	29140711	rs4666128	G	A	0.23	0.92	$5.78x10^{-6}$	0.21	0.92	$5.35x10^{-4}$	$9.25x10^{-9}$
2	29134513	rs4389288	T	C	0.23	0.91	$4.99x10^{-6}$	0.21	0.92	$6.15x10^{-4}$	$9.25x10^{-9}$
2	29129418	rs11688605	A	G	0.23	0.91	$4.07x10^{-6}$	0.21	0.92	$7.42x10^{-4}$	$9.34x10^{-9}$
2	29131987	rs66692018	A	G	0.23	0.91	$4.20x10^{-6}$	0.21	0.92	$7.24x10^{-4}$	$9.34x10^{-9}$

2	29129682	rs57693531	T	G	0.23	0.91	$4.06x10^{-6}$	0.21	0.92	$7.43x10^{-4}$	$9.35x10^{-9}$
2	29129923	rs11688844	A	T	0.23	0.91	$4.09x10^{-6}$	0.21	0.92	$7.44x10^{-4}$	$9.35x10^{-9}$
2	29130224	rs11684312	C	T	0.23	0.92	$4.09x10^{-6}$	0.21	0.92	$7.44x10^{-4}$	$9.36x10^{-9}$
2	29161707	rs67612857	G	C	0.23	0.92	$6.99x10^{-6}$	0.21	0.92	$4.56x10^{-4}$	$9.36x10^{-9}$
2	29138436	rs4273183	G	A	0.23	0.92	$5.32x10^{-6}$	0.21	0.92	$5.69x10^{-4}$	$9.48x10^{-9}$
2	29144892	rs12620770	C	T	0.23	0.92	$7.99x10^{-6}$	0.21	0.92	$3.83x10^{-4}$	$9.68x10^{-9}$
2	29133688	rs12620330	C	T	0.23	0.92	$7.18x10^{-6}$	0.21	0.92	$4.77x10^{-4}$	$1.02x10^{-8}$
2	29135979	rs6743535	G	C	0.23	0.92	$4.81x10^{-6}$	0.21	0.92	$6.89x10^{-4}$	$1.03x10^{-8}$
2	29143425	rs12471649	T	C	0.23	0.91	$7.91x10^{-6}$	0.21	0.92	$4.13x10^{-4}$	$1.04x10^{-8}$
2	29156360	rs4371318	G	A	0.23	0.92	$7.85x10^{-6}$	0.21	0.92	$4.35x10^{-4}$	$1.04x10^{-8}$
2	29111099	rs12613887	C	T	0.23	0.92	$6.36x10^{-6}$	0.21	0.92	$5.48x10^{-4}$	$1.05x10^{-8}$
2	29120733	rs4577244	T	C	0.23	0.91	$2.52x10^{-6}$	0.23	0.92	$1.18x10^{-3}$	$1.05x10^{-8}$
2	29137773	rs4666137	G	A	0.23	0.92	$5.03x10^{-6}$	0.21	0.92	$6.57x10^{-4}$	$1.06x10^{-8}$
2	29123191	rs12616009	C	G	0.23	0.91	$6.61x10^{-6}$	0.21	0.92	$5.51x10^{-4}$	$1.08x10^{-8}$
2	29171085	rs12465505	A	T	0.23	0.91	$7.69x10^{-6}$	0.21	0.92	$4.49x10^{-4}$	$1.09x10^{-8}$
2	29111060	rs12622122	C	A	0.23	0.91	$2.14x10^{-6}$	0.23	0.92	$1.32x10^{-3}$	$1.11x10^{-8}$
2	29170623	rs11680458	G	T	0.24	0.92	$7.79x10^{-6}$	0.21	0.92	$4.48x10^{-4}$	$1.11x10^{-8}$
2	29133600	rs12620306	C	T	0.23	0.92	$6.08x10^{-6}$	0.22	0.92	$6.12x10^{-4}$	$1.12x10^{-8}$
2	29171544	rs4666142	A	G	0.23	0.91	$9.38x10^{-6}$	0.21	0.92	$4.11x10^{-4}$	$1.15x10^{-8}$
2	29171381	rs4665437	C	T	0.23	0.92	$9.41x10^{-6}$	0.21	0.92	$4.11x10^{-4}$	$1.17x10^{-8}$
2	29137632	rs56194243	T	C	0.24	0.91	$6.57x10^{-6}$	0.21	0.92	$5.83x10^{-4}$	$1.20x10^{-8}$
2	29162904	rs12465258	G	T	0.23	0.92	$1.15x10^{-5}$	0.22	0.91	$3.38x10^{-4}$	$1.21x10^{-8}$
2	29171288	rs12477538	C	T	0.23	0.92	$7.91x10^{-6}$	0.21	0.92	$5.04x10^{-4}$	$1.27x10^{-8}$
2	29114372	rs7580240	C	T	0.23	0.92	$3.24x10^{-6}$	0.22	0.92	$1.21x10^{-3}$	$1.31x10^{-8}$
2	29167692	rs3924271	G	A	0.23	0.92	$7.97x10^{-6}$	0.21	0.92	$5.57x10^{-4}$	$1.37x10^{-8}$
2	29177823	rs72786123	A	G	0.23	0.91	$6.65x10^{-6}$	0.22	0.92	$7.35x10^{-4}$	$1.41x10^{-8}$
2	29170676	rs1131880	G	C	0.23	0.92	$8.58x10^{-6}$	0.21	0.92	$5.28x10^{-4}$	$1.43x10^{-8}$
2	29179452	rs12472404	G	C	0.23	0.92	$3.47x10^{-6}$	0.22	0.92	$1.31x10^{-3}$	$1.46x10^{-8}$
2	29167678	rs3924272	C	T	0.24	0.92	$6.38x10^{-6}$	0.22	0.92	$7.54x10^{-4}$	$1.49x10^{-8}$
2	29174105	rs4666144	C	T	0.23	0.92	$9.82x10^{-6}$	0.22	0.92	$5.17x10^{-4}$	$1.49x10^{-8}$
2	29136653	rs4666134	T	C	0.23	0.91	$1.00x10^{-5}$	0.21	0.92	$5.38x10^{-4}$	$1.51x10^{-8}$
2	29137687	rs4666136	C	T	0.23	0.92	$7.77x10^{-6}$	0.21	0.92	$6.41x10^{-4}$	$1.53x10^{-8}$
2	29111106	rs12622793	C	T	0.24	0.92	$2.66x10^{-6}$	0.22	0.93	$1.67x10^{-3}$	$1.57x10^{-8}$
2	29177344	rs4666151	C	A	0.23	0.92	$6.94x10^{-6}$	0.22	0.92	$7.88x10^{-4}$	$1.63x10^{-8}$

2	29136623	rs4666133	C	T	0.23	0.92	1.09×10^{-5}	0.21	0.92	5.12×10^{-4}	1.67×10^{-8}
2	29136685	rs12472068	T	C	0.23	0.91	1.09×10^{-5}	0.21	0.92	5.12×10^{-4}	1.67×10^{-8}
2	29128527	chr2:29128527:D	TTC	T	0.23	0.92	1.18×10^{-5}	0.22	0.92	4.96×10^{-4}	1.73×10^{-8}
2	29168848	rs3885709	A	G	0.24	0.91	9.44×10^{-6}	0.21	0.92	6.57×10^{-4}	1.85×10^{-8}
2	29132034	rs66854259	C	T	0.23	0.92	9.15×10^{-6}	0.21	0.92	7.21×10^{-4}	1.88×10^{-8}
2	29111818	rs7371404	A	C	0.23	0.91	2.97×10^{-6}	0.22	0.93	1.67×10^{-3}	1.89×10^{-8}
2	29151714	chr2:29151714:I	A	AT	0.23	0.91	1.53×10^{-5}	0.21	0.92	4.39×10^{-4}	1.93×10^{-8}
2	29112923	rs4666125	T	C	0.23	0.91	3.22×10^{-6}	0.22	0.93	1.78×10^{-3}	2.15×10^{-8}
2	29134578	chr2:29134578:D	GTC	G	0.23	0.92	1.18×10^{-5}	0.22	0.92	6.33×10^{-4}	2.26×10^{-8}
2	29116710	chr2:29116710:I	C	CCAGAG	0.23	0.91	3.38×10^{-6}	0.20	0.92	2.30×10^{-3}	2.40×10^{-8}
2	29171587	chr2:29171587:D	TTG	T	0.24	0.92	1.79×10^{-5}	0.21	0.92	4.48×10^{-4}	2.53×10^{-8}
2	29142411	rs78757684	A	C	0.23	0.90	8.88×10^{-6}	0.20	0.92	1.15×10^{-3}	2.55×10^{-8}
2	29144512	rs13008915	C	T	0.24	0.93	6.00×10^{-5}	0.28	0.92	9.61×10^{-4}	1.79×10^{-7}
2	29149048	chr2:29149048:D	GA	G	0.23	0.91	1.75×10^{-5}	0.23	0.93	5.65×10^{-3}	2.41×10^{-7}
2	29152456	rs7421144	A	G	0.21	0.94	7.93×10^{-5}	0.28	0.92	1.07×10^{-3}	2.74×10^{-7}

P-values for BRCA1 carriers were estimated by a kinship-adjusted retrospective likelihood approach.
P-values for iCOGS/BCAC and for meta-analysis were estimated by Chi-square

Supplementary Table 8. ER-negative breast cancer association tests in BCAC, *BRCA1* carriers, and combined analysis for 13q22 SNPs after local reimputation

					iCOGS/BCAC			BRCA1			Meta-analysis
**Chr**	**Position**	**SNP**	**A2**	**A1**	**EAF**	**OR**	**P**	**EAF**	**HR**	**P**	**P**
13	73957681	rs6562760	G	A	0.23	0.92	$1.85x10^{-5}$	0.24	0.88	$2.85x10^{-6}$	$4.98x10^{-10}$
13	73960952	rs2181965	G	A	0.23	0.92	$2.16x10^{-5}$	0.24	0.88	$2.39x10^{-6}$	$5.04x10^{-10}$
13	73964519	rs8002929	G	A	0.23	0.93	$2.52x10^{-5}$	0.24	0.88	$1.71x10^{-6}$	$5.35x10^{-10}$
13	73960825	rs2147513	G	C	0.23	0.92	$1.93x10^{-5}$	0.24	0.88	$3.12x10^{-6}$	$5.50x10^{-10}$
13	73962520	rs9600121	G	A	0.23	0.92	$2.41x10^{-5}$	0.23	0.88	$3.01x10^{-6}$	$7.00x10^{-10}$
13	73956279	rs4143044	G	T	0.23	0.92	$2.36x10^{-5}$	0.23	0.88	$2.97x10^{-6}$	$7.09x10^{-10}$
13	73954561	rs927683	C	T	0.23	0.92	$2.48x10^{-5}$	0.23	0.88	$3.22x10^{-6}$	$7.80x10^{-10}$
13	73806982	rs12870942	T	C	0.32	1.08	$2.71x10^{-7}$	0.31	1.06	$1.01x10^{-2}$	$3.75x10^{-8}$
13	73967507	rs4885096	C	T	0.30	0.93	$5.18x10^{-5}$	0.32	0.88	$1.86x10^{-4}$	$3.99x10^{-8}$
13	73811471	rs17181761	A	C	0.32	1.08	$3.44x10^{-7}$	0.31	1.06	$9.29x10^{-3}$	$4.23x10^{-8}$
13	73814192	rs9530172	A	G	0.32	1.09	$2.97x10^{-7}$	0.31	1.06	$1.01x10^{-2}$	$4.23x10^{-8}$
13	73814441	rs9543287	C	G	0.32	1.08	$3.60x10^{-7}$	0.31	1.06	$9.85x10^{-3}$	$4.41x10^{-8}$
13	73813803	rs9573140	A	G	0.32	1.08	$3.77x10^{-7}$	0.31	1.06	$1.11x10^{-2}$	$5.35x10^{-8}$
13	73814697	rs9530173	A	G	0.32	1.08	$3.68x10^{-7}$	0.31	1.06	$1.14x10^{-2}$	$5.41x10^{-8}$
13	73813413	rs7989788	T	C	0.37	1.08	$5.22x10^{-7}$	0.35	1.06	$1.03x10^{-2}$	$8.89x10^{-8}$
13	73813403	rs7989653	T	C	0.37	1.08	$4.77x10^{-7}$	0.35	1.05	$1.17x10^{-2}$	$1.09x10^{-7}$
13	73812537	rs9543285	A	G	0.33	1.08	$6.54x10^{-7}$	0.32	1.05	$1.75x10^{-2}$	$1.62x10^{-7}$

P-values for BRCA1 carriers were estimated by a kinship-adjusted retrospective likelihood approach.
P-values for iCOGS/BCAC and for meta-analysis were estimated by Chi-square

Supplementary Table 9. Breast cancer associations adjusted for most significant SNPs in individual regions.

rs#	Freq	P
**13q22 adjusted for rs17181761**		
rs8002929	0.23	4.52×10^{-8}
rs2181965	0.23	5.49×10^{-8}
rs4143044	0.23	7.02×10^{-8}
rs6562760	0.23	7.12×10^{-8}
rs9600121	0.23	7.28×10^{-8}
rs927683	0.23	7.47×10^{-8}
rs2147513	0.23	7.51×10^{-8}
rs4885096	0.31	9.28×10^{-7}
rs9600122	0.11	2.15×10^{-4}
rs9543357	0.10	2.48×10^{-4}
rs9543285	0.32	7.12×10^{-3}
rs7989799	0.28	0.018
rs7988505	0.28	0.019
rs9592895	0.33	0.019
rs11841589	0.27	0.021
**13q adjusted for rs6562760**		
rs17181761	0.32	5.97×10^{-6}
rs12870942	0.31	5.97×10^{-6}
rs9543287	0.32	6.35×10^{-6}
rs9530172	0.32	6.65×10^{-6}
rs9573140	0.32	7.38×10^{-6}
rs9530173	0.32	7.44×10^{-6}
rs9543285	0.32	2.58×10^{-5}
rs7989788	0.36	3.08×10^{-5}
rs7989653	0.36	3.28×10^{-5}
rs9592895	0.33	3.54×10^{-4}
rs7989799	0.28	1.07×10^{-3}
rs7988505	0.28	1.15×10^{-3}
rs11841589	0.27	1.41×10^{-3}
**2p23.2 adjusted for rs67073037**		
chr2:28996536:D	0.29	0.056
rs7580078	0.29	0.068
rs6728459	0.29	0.088

P-values were estimated by Chi-square.

Supplementary Table 10. Association of novel loci with overall breast cancer and ER-positive breast cancer in the general population (iCOGS/BCAC) and breast cancer in *BRCA2* mutation carriers

					**iCOGS/BCAC breast cancer**		**iCOGS/BCAC ER-positive**			***BRCA2* carriers**	
**locus**	**rs#**	**position**	**ref**	**eff**	**OR (95%CI)**	**p**	**OR (95%CI)**	**p**	**p^{het}**	**HR (95%CI)**	**P**
2p23.2	rs67073037	29119585	A	T	0.98 (0.96-1.00)	0.069	1.01 (0.99-1.03)	0.65	$4.45x10^{-6}$	1.01 (0.94-1.09)	0.70
2q33	rs115635831	201451839	A	G	1.14 (1.10-1.18)	$4x10^{-4}$	1.06 (1.01-1.10)	0.23	$2.9x10^{-4}$	1.05 (0.85-0.32)	0.65
13q22	rs6562760	73957681	G	A	0.97 (0.95-0.99)	$6.5x10^{-3}$	0.98 (0.96-1.00)	0.073	0.028	0.98 (0.92-1.05)	0.51
13q22	rs17181761	73811471	A	C	1.04 (1.02-1.06)	$6.4x10^{-4}$	1.03 (1.01-1.05)	0.030	$5.82x10^{-3}$	1.02 (0.96-1.09)	0.46

P-values for BRCA2 carriers were estimated by a kinship-adjusted retrospective likelihood approach.
P-values for iCOGS/BCAC were estimated by Chi-square.
P-values for heterogeneity (p^{het} –) between ER-negative and ER-positive breast cancer in the general population (iCOGS/BCAC) were evaluated by Q test.

Supplementary Table 11. Association of novel loci with ER-negative breast cancer in the general population (iCOGS/BCAC and GWAS) when excluding studies oversampled for bilateral cases.

SNP	beta_ER-negative	SE_ER-negative	p-value	OR	LCI	UCI
**iCOGS ER-negative**						
rs67073037	-0.105	0.023	$5.96x10^{-6}$	0.90	0.86	0.94
rs188686860	0.301	0.069	$1.45x10^{-5}$	1.35	1.18	1.55
rs6562760	-0.058	0.023	$1.18x10^{-2}$	0.94	0.90	0.99
rs12870942	0.077	0.021	$1.93x10^{-4}$	1.08	1.04	1.12
**iCOGS BBCS ER-negative**						
rs67073037	-0.176	0.183	0.33	0.84	0.59	1.20
rs188686860	0.792	0.425	0.06	2.21	0.96	5.08
rs6562760	-0.378	0.187	0.04	0.69	0.48	0.99
rs12870942	0.105	0.150	0.48	1.11	0.83	1.49
**iCOGS ER-negative excluding BBCS**						
rs67073037	-0.104	0.023	$9.37x10^{-6}$	0.90	0.86	0.94
rs188686860	0.290	0.070	$3.83x10^{-5}$	1.34	1.16	1.53
rs6562760	-0.054	0.023	$1.86x10^{-2}$	0.95	0.91	0.99
rs12870942	0.076	0.021	$2.37x10^{-4}$	1.08	1.04	1.12
**ER-negative meta-analysis GWAS+iCOGS**						
rs67073037	-0.088	0.019	$2.63x10^{-6}$	0.92	0.88	0.92
rs188686860	0.280	0.062	$6.58x10^{-6}$	1.32	1.17	1.32
rs6562760	-0.078	0.019	$2.34x10^{-5}$	0.93	0.89	0.93
rs12870942	0.083	0.017	$7.41x10^{-7}$	1.09	1.05	1.09
**ER-negative meta-analysis GWAS+iCOGS excluding BBCS**						
rs67073037	-0.087	0.019	$3.94x10^{-6}$	0.92	0.88	0.92
rs188686860	0.271	0.063	$1.65x10^{-5}$	1.31	1.16	1.31
rs6562760	-0.076	0.019	$4.09x10^{-5}$	0.93	0.89	0.93
rs12870942	0.083	0.017	$9.08x10^{-7}$	1.09	1.05	1.09

OR: odds ratio; LCI: lower confidence interval; UCI: upper confidence interval; SE: standard error; P-values were estimated by Chi-square.

Supplementary Table 12. SNPs most significantly associated with ER-negative breast cancer among *BRCA1* carriers in the three ER-negative loci

			ER-positive			ER-negative			
**Chr**	**Location**	**rs**	**HR**	**95%CI**	**P**	**HR**	**95%CI**	**P**	**#P-het**
2p23.2	29120733	*rs4577244	0.92	0.83-1.03	0.16	0.91	0.86-0.97	$1.87x10^{-3}$	0.82
2q33	201451839	*rs115635831	0.90	0.58-1.39	0.63	1.32	1.10-1.58	$2.82x10^{-3}$	0.12
13q22	73814891	rs11841589	0.95	0.85-1.06	0.33	0.92	0.87-0.97	$2.45x10^{-3}$	0.62
13q22	73813803	*rs9573140	1.03	0.93-1.13	0.61	1.07	1.02-1.13	0.01	0.49
13q22	73957681	*rs6562760	0.88	0.79-0.98	0.02	0.89	0.84-0.95	$1.73x10^{-4}$	0.78
13q22	73964519	*rs8002929	0.88	0.79-0.98	0.03	0.89	0.84-0.94	$9.77x10^{-5}$	0.85

# p-value for the heterogeneity of associations with tumor subtypes were estimated by a kinship-adjusted retrospective likelihood approach.
* Genotyped SNPs

Supplementary Table 13. *BRCA1* competing risks association test results and HR estimates for ovarian and breast cancer for the most significantly associated genotyped SNP from each locus.

				BRCA1 ovarian		BRCA1 breast	
**Chr**	**Location**	**rs#**	**r^2**	**HR (95%CI)**	**P**	**HR (95%)**	**P**
2p23.2	29120733	rs4577244	1.0	0.98 (0.89-1.08)	0.73	0.91 (0.87-0.96)	$5.24x10^{-4}$
2q33	201451839	rs115635831	1.0	1.04 (0.74-1.45)	0.83	1.23 (1.04-1.46)	0.016
13q22	73957681	rs6562760	1.0	1.02 (0.93-1.12)	0.67	0.89 (0.85-0.94)	$1.85x10^{-5}$
13q22	73813803	rs9573140	1.0	0.99 (0.92-1.09)	0.96	1.06 (1.01-1.11)	0.014

P-values were estimated by a kinship-adjusted retrospective likelihood approach.

Supplementary Table 14. Assessment of interaction between index SNPs from novel loci and known risk SNPs in BCAC/iCOGS.

	Chr 2p23			Chr2q33		
**SNP**	**Adj_new rs67073037 pvalue**	**adj_old rs67073037 pvalue**	**rs67073037* pvalue**	**Adj new rs114962751 pvalue**	**Adj old rs114962751 pvalue**	**rs114962751* pvalue**
rs616488	9.10E-04	2.87E-05	7.60E-01	4.60E-04	4.98E-07	9.01E-01
rs12022378	2.61E-03	3.87E-02	1.82E-01	1.75E-05	9.78E-02	6.91E-01
rs11249433	1.34E-02	7.35E-01	6.02E-01	1.20E-05	7.64E-01	1.00E-01
rs12048493	7.17E-04	5.94E-01	4.71E-01	2.18E-04	1.70E-01	4.53E-01
rs12405132	1.59E-03	3.20E-01	8.03E-01	2.38E-04	3.38E-01	5.65E-01
rs4245739	3.99E-01	4.74E-07	3.43E-01	1.08E-01	1.42E-12	7.81E-01
rs6678914	5.77E-03	6.54E-04	9.28E-01	1.13E-05	4.70E-05	9.14E-02
rs72755295	1.26E-05	2.89E-02	6.13E-01	2.52E-06	1.32E-03	7.76E-01
rs12710696	2.69E-02	8.92E-05	8.42E-01	6.36E-02	1.35E-06	6.42E-01
rs4849887	2.23E-01	3.18E-02	8.19E-01	2.54E-01	3.54E-03	9.97E-01
rs1550623	4.84E-02	3.54E-01	4.68E-01	3.95E-01	9.55E-02	5.60E-01
rs2016394	5.17E-03	8.27E-01	8.49E-01	1.06E-02	8.77E-01	6.30E-01
rs1045485	1.62E-05	9.68E-02	3.41E-01	2.87E-06	2.94E-01	4.43E-01
rs13387042	4.16E-03	4.27E-02	7.31E-01	2.96E-02	1.66E-02	4.31E-01
rs16857609	4.33E-04	5.62E-03	7.80E-01	1.10E-04	1.63E-04	7.72E-01
rs6762644	2.74E-04	9.19E-01	3.18E-01	7.25E-02	5.67E-01	5.95E-02
rs4973768	9.77E-04	1.53E-01	4.12E-01	1.64E-04	8.45E-03	2.49E-01
rs12493607	4.63E-04	9.06E-01	5.56E-01	1.23E-03	5.45E-01	8.37E-01
rs6796502	1.89E-05	6.47E-02	4.86E-01	1.53E-06	1.15E-01	2.73E-01
rs1053338	4.24E-04	5.08E-02	5.04E-01	3.26E-05	5.88E-02	9.53E-01
rs9790517	1.89E-05	9.93E-01	1.99E-01	6.66E-05	2.62E-01	8.30E-01
rs6828523	3.83E-05	8.13E-01	6.38E-01	7.84E-04	7.19E-01	6.74E-02
rs10069690	2.64E-02	6.66E-12	8.10E-02	3.17E-02	9.01E-12	1.15E-02
rs2736108	4.21E-05	4.71E-07	2.14E-01	1.32E-08	1.56E-06	1.42E-03
rs7726159	5.00E-03	1.79E-04	7.13E-01	7.18E-03	2.03E-05	3.41E-01
rs13162653	2.44E-02	1.06E-01	7.13E-01	1.19E-01	1.01E-02	1.37E-01
rs2012709	1.92E-03	6.68E-01	5.12E-01	1.27E-02	3.09E-01	7.26E-01
rs10941679	3.72E-06	9.16E-01	5.13E-02	1.34E-03	1.28E-01	4.42E-01
rs10472076	3.84E-03	2.34E-02	9.34E-01	4.12E-05	2.65E-03	2.40E-01
rs1353747	2.49E-04	7.67E-02	4.20E-01	4.44E-06	6.32E-03	5.67E-01
rs889312	1.36E-01	6.75E-02	7.63E-01	9.30E-02	9.05E-03	8.53E-01
rs7707921	1.38E-01	3.13E-01	9.15E-01	5.15E-01	4.51E-01	9.33E-03
rs1432679	1.15E-01	7.57E-03	3.84E-01	8.83E-03	6.11E-05	9.56E-01
rs11242675	4.25E-01	3.83E-03	1.27E-01	2.95E-01	2.81E-02	1.49E-01
rs204247	1.67E-02	5.86E-01	9.58E-01	1.07E-04	7.09E-01	1.15E-01

rs150750171	7.11E-03	2.62E-02	7.77E-01	6.73E-01	4.65E-02	3.87E-04
rs17529111	3.55E-04	1.28E-01	9.32E-01	1.02E-04	4.60E-02	9.34E-01
rs12662670	7.45E-05	2.86E-07	7.49E-01	3.81E-05	1.55E-09	3.78E-01
rs2046210	1.24E-04	1.02E-07	2.72E-01	1.65E-02	2.54E-13	1.62E-01
rs6964587	5.79E-03	1.33E-01	8.56E-01	5.13E-03	1.01E-01	5.76E-01
rs4593472	4.39E-05	2.06E-01	1.45E-01	8.09E-07	9.77E-01	2.48E-02
rs720475	2.85E-02	4.42E-01	6.02E-02	1.27E-08	9.84E-01	1.79E-03
rs9693444	8.51E-02	2.86E-03	8.04E-01	3.47E-03	1.44E-04	3.75E-01
rs13365225	3.09E-05	2.19E-03	4.04E-01	5.84E-06	2.85E-03	3.73E-01
rs2943559	4.34E-05	1.04E-01	9.97E-01	1.35E-05	4.62E-02	7.93E-01
rs6472903	4.54E-02	7.45E-02	5.05E-01	6.84E-05	1.23E-03	8.87E-03
rs13267382	7.95E-03	7.89E-03	4.79E-01	2.42E-02	5.49E-03	9.54E-01
rs11780156	7.82E-04	1.94E-02	4.64E-01	1.04E-07	5.48E-03	1.84E-02
rs13281615	3.46E-03	2.78E-01	9.43E-01	5.60E-04	1.40E-01	6.05E-01
rs1011970	3.25E-04	2.51E-04	8.41E-01	2.16E-04	1.61E-05	5.57E-01
rs10759243	3.49E-04	9.67E-01	6.45E-01	2.38E-03	7.55E-01	4.24E-01
rs865686	7.41E-02	3.78E-01	6.92E-01	2.20E-04	2.76E-01	1.17E-01
rs2380205	9.45E-02	3.84E-01	1.65E-01	1.62E-01	7.61E-01	4.10E-02
rs11814448	2.27E-05	1.73E-02	7.51E-01	2.98E-06	6.98E-03	8.96E-01
rs7072776	4.90E-01	3.91E-03	2.26E-01	8.15E-02	8.13E-03	8.32E-01
rs10995190	9.52E-04	5.46E-04	3.45E-01	9.62E-04	1.03E-07	1.17E-01
rs704010	2.89E-01	5.89E-01	2.04E-01	7.35E-05	1.90E-01	6.56E-02
rs7904519	4.40E-03	3.43E-02	8.17E-01	2.48E-02	7.31E-03	4.25E-01
rs11199914	2.74E-03	4.83E-01	8.16E-01	2.25E-02	6.85E-01	9.67E-02
rs2981579	2.45E-03	8.16E-02	3.50E-01	7.62E-03	1.61E-01	7.66E-01
rs3817198	7.86E-04	3.55E-02	8.14E-01	1.21E-04	3.47E-03	5.56E-01
c11_pos69088342	1.15E-05	3.49E-01	4.61E-01	2.16E-04	1.95E-01	3.27E-02
rs3903072	5.54E-03	1.33E-01	8.76E-01	1.96E-05	1.40E-01	8.30E-02
rs554219	2.94E-05	8.41E-01	5.21E-01	1.89E-04	5.74E-01	3.57E-01
rs78540526	1.80E-05	6.95E-01	5.46E-01	3.12E-04	5.65E-01	6.00E-02
rs11820646	6.84E-01	2.55E-03	3.33E-02	1.12E-01	4.87E-02	3.72E-01
rs12422552	5.91E-05	4.22E-01	3.18E-01	3.19E-04	6.95E-02	9.45E-01
rs10771399	5.71E-04	6.59E-05	3.31E-01	3.02E-06	1.59E-08	3.73E-01
rs17356907	8.79E-04	8.56E-03	8.97E-01	4.70E-04	1.23E-03	9.44E-01
rs1292011	1.66E-02	7.72E-01	5.77E-01	2.83E-04	4.92E-01	4.89E-01
rs11571833	2.25E-05	1.61E-04	5.10E-01	1.98E-06	2.30E-05	8.00E-01
rs2236007	8.55E-03	6.74E-01	1.06E-01	3.32E-05	9.52E-02	6.19E-01
rs2588809	7.86E-01	5.20E-01	2.84E-01	4.02E-02	9.39E-01	4.86E-01
rs999737	3.83E-04	3.74E-02	8.98E-01	4.77E-04	9.58E-03	6.09E-01
rs11627032	4.03E-05	1.62E-03	1.91E-01	1.34E-02	1.44E-03	1.15E-01
rs941764	4.00E-02	2.43E-02	1.52E-01	3.68E-04	7.84E-02	8.28E-01

rs11075995	2.59E-02	6.31E-05	5.08E-01	1.28E-03	2.06E-05	1.29E-01
rs17817449	4.33E-04	7.24E-06	4.06E-01	1.84E-03	6.63E-07	9.47E-01
rs3803662	1.94E-02	4.91E-08	5.14E-01	9.98E-03	5.26E-10	4.49E-01
rs13329835	2.43E-03	2.06E-01	4.27E-01	3.57E-04	3.71E-01	6.91E-01
chr17.29230520.D	1.08E-04	2.93E-02	4.95E-01	1.48E-05	4.19E-02	3.82E-01
rs6504950	2.72E-03	4.06E-01	6.38E-01	3.77E-03	1.12E-01	2.72E-01
rs745570	1.45E-04	9.31E-04	1.08E-01	3.52E-05	8.72E-03	9.02E-02
rs1436904	5.72E-03	9.91E-01	8.65E-01	7.34E-02	6.83E-01	7.51E-02
rs527616	2.24E-01	1.09E-01	2.92E-01	8.25E-06	1.04E-01	1.61E-02
rs6507583	9.44E-05	6.99E-01	6.00E-01	3.96E-06	4.48E-01	6.73E-01
rs2363956	1.23E-03	2.47E-08	3.62E-01	2.17E-04	1.28E-09	2.27E-01
rs4808801	2.79E-02	7.48E-03	2.21E-01	9.18E-04	1.57E-05	9.83E-01
rs8170	1.32E-04	4.52E-05	6.95E-01	7.28E-04	1.33E-07	3.81E-01
rs3760982	5.25E-02	2.28E-01	5.60E-01	2.00E-02	4.24E-02	7.26E-01
rs2823093	1.69E-03	4.30E-01	7.64E-01	1.14E-04	2.47E-01	7.17E-01
rs132390	6.73E-02	4.08E-02	2.10E-01	4.40E-01	1.16E-01	9.35E-01
rs17879961	8.66E-06	4.64E-01	4.31E-01	2.11E-06	7.46E-01	8.55E-01
rs6001930	3.75E-04	6.06E-04	3.86E-01	1.91E-03	1.48E-03	5.31E-03

	Chr 13q22			Chr13q22		
**snp**	**Adj new rs6562760 pvalue**	**Adj old rs6562760 pvalue**	**rs6562760* pvalue**	**Adj new rs12870942 pvalue**	**Adj old rs12870942 pvalue**	**rs12870942* pvalue**
rs616488	6.16E-03	2.83E-01	4.09E-01	6.52E-03	7.66E-05	6.57E-01
rs12022378	2.30E-02	9.94E-01	5.46E-01	2.08E-04	2.14E-01	8.83E-01
rs11249433	2.22E-01	3.47E-01	3.15E-01	7.00E-02	3.64E-01	1.92E-01
rs12048493	2.31E-02	4.17E-01	6.97E-01	1.87E-02	5.92E-01	5.85E-01
rs12405132	1.35E-02	9.11E-01	5.92E-01	9.28E-03	3.01E-01	6.90E-01
rs4245739	2.76E-01	7.97E-03	9.27E-01	2.96E-01	2.31E-08	5.03E-01
rs6678914	4.57E-02	1.15E-01	9.80E-01	6.84E-03	1.30E-03	9.46E-01
rs72755295	4.46E-03	4.45E-01	6.39E-01	5.49E-05	3.95E-02	7.71E-01
rs12710696	3.20E-01	2.60E-02	6.18E-01	1.89E-01	5.85E-05	4.35E-01
rs4849887	3.76E-01	6.55E-01	1.06E-01	2.28E-01	2.36E-02	8.05E-01
rs1550623	8.91E-03	2.00E-02	7.22E-02	9.54E-02	1.14E-01	6.67E-01
rs2016394	1.63E-03	1.33E-01	9.81E-02	4.08E-02	5.89E-01	5.33E-01
rs1045485	7.48E-04	3.94E-01	1.47E-01	5.40E-04	1.45E-01	5.10E-01
rs13387042	2.99E-03	6.09E-01	1.48E-01	1.16E-02	1.03E-01	9.78E-01
rs16857609	1.54E-03	9.97E-03	1.89E-01	5.21E-05	4.19E-04	2.26E-01
rs6762644	2.50E-02	5.34E-01	7.65E-01	1.52E-02	7.56E-01	6.06E-01
rs4973768	1.06E-01	5.19E-01	7.86E-01	4.34E-03	4.43E-02	7.20E-01
rs12493607	3.37E-01	1.85E-01	9.42E-02	3.33E-03	6.92E-01	9.35E-01

rs6796502	7.50E-03	3.95E-01	8.33E-01	2.89E-05	4.34E-01	4.72E-01
rs1053338	1.40E-02	7.65E-01	6.64E-01	3.67E-04	2.61E-01	6.84E-01
rs9790517	1.89E-03	1.54E-01	2.68E-01	2.58E-04	2.98E-01	6.84E-01
rs6828523	3.15E-02	3.71E-01	2.25E-01	7.29E-04	8.80E-01	3.72E-01
rs10069690	1.43E-02	4.16E-03	8.19E-01	3.23E-05	4.52E-10	1.61E-01
rs2736108	1.29E-02	1.06E-01	7.08E-01	3.30E-05	4.18E-03	1.41E-01
rs7726159	4.78E-02	1.57E-01	8.38E-01	1.53E-03	7.42E-04	8.10E-01
rs13162653	6.01E-01	2.20E-02	1.23E-01	2.75E-04	4.91E-01	1.56E-01
rs2012709	3.02E-01	6.24E-01	3.48E-01	1.87E-03	2.01E-01	4.59E-01
rs10941679	1.61E-02	4.94E-01	9.45E-01	6.87E-03	5.90E-01	3.56E-01
rs10472076	1.95E-01	9.60E-01	3.02E-01	1.87E-03	2.57E-02	7.82E-01
rs1353747	6.33E-03	2.67E-01	9.41E-01	1.21E-03	1.70E-03	1.18E-01
rs889312	8.78E-01	4.15E-02	2.34E-01	1.77E-02	1.69E-01	4.78E-01
rs7707921	9.60E-01	5.44E-01	2.62E-01	6.76E-01	9.72E-01	2.29E-01
rs1432679	5.96E-02	2.37E-01	7.52E-01	2.53E-02	3.04E-03	9.64E-01
rs11242675	4.48E-01	8.88E-01	4.13E-01	4.67E-02	8.88E-02	9.36E-01
rs204247	1.02E-01	8.17E-01	9.95E-01	3.68E-02	5.37E-01	8.17E-01
rs150750171	8.67E-03	9.03E-02	4.31E-01	3.34E-05	1.91E-03	8.00E-02
rs17529111	3.69E-04	1.36E-02	6.28E-02	2.08E-04	7.61E-02	6.25E-01
rs12662670	2.74E-03	6.58E-03	6.24E-01	6.96E-05	6.37E-06	9.29E-01
rs2046210	1.79E-01	7.46E-02	2.95E-01	1.09E-02	4.19E-07	5.35E-01
rs6964587	6.29E-03	1.29E-01	3.48E-01	5.11E-02	6.50E-01	2.35E-01
rs4593472	7.04E-03	5.43E-01	4.13E-01	1.57E-03	9.25E-01	7.77E-01
rs720475	3.40E-02	5.73E-01	7.02E-01	4.81E-03	3.35E-01	4.31E-01
rs9693444	1.51E-01	1.69E-01	9.32E-01	7.44E-03	2.41E-02	3.83E-01
rs13365225	2.97E-03	6.59E-01	4.39E-01	5.07E-03	8.38E-04	1.32E-01
rs2943559	1.08E-02	9.65E-01	4.39E-01	1.29E-04	2.35E-01	6.75E-01
rs6472903	5.40E-02	4.85E-02	2.99E-01	5.21E-02	1.45E-02	5.21E-01
rs13267382	1.43E-02	9.50E-01	2.42E-01	2.25E-01	8.75E-03	3.89E-01
rs11780156	2.75E-04	6.72E-03	4.75E-02	9.22E-04	2.02E-01	5.34E-01
rs13281615	5.51E-01	2.29E-01	6.53E-02	1.09E-02	4.28E-01	7.37E-01
rs1011970	3.06E-02	3.57E-01	4.65E-01	1.07E-03	5.58E-03	5.25E-01
rs10759243	6.72E-02	6.56E-01	5.16E-01	5.50E-04	5.42E-01	6.84E-01
rs865686	4.89E-02	4.16E-01	5.76E-01	7.49E-03	2.94E-01	4.98E-01
rs2380205	6.18E-05	1.18E-02	7.19E-03	6.99E-03	9.66E-01	9.84E-01
rs11814448	9.88E-04	1.36E-02	1.14E-01	1.24E-04	4.79E-01	6.64E-02
rs7072776	1.80E-01	2.77E-01	9.18E-01	2.71E-01	1.41E-01	4.77E-01
rs10995190	1.35E-03	4.39E-01	2.47E-01	9.03E-04	3.94E-05	5.18E-01
rs704010	4.06E-01	1.84E-01	4.41E-01	1.17E-02	3.73E-01	6.28E-01
rs7904519	4.35E-02	2.15E-01	8.34E-01	3.05E-04	3.12E-03	2.11E-01
rs11199914	4.65E-02	9.98E-01	7.68E-01	7.84E-05	1.62E-01	2.13E-01

rs2981579	6.81E-01	8.16E-03	2.33E-02	4.32E-03	6.34E-01	4.20E-01
rs3817198	1.76E-03	2.22E-02	1.79E-01	1.21E-05	8.25E-04	6.46E-02
c11_pos69088342	8.60E-03	9.58E-01	4.62E-01	1.05E-04	3.53E-01	6.17E-01
rs3903072	1.27E-01	3.18E-01	7.07E-01	7.51E-03	2.22E-01	9.11E-01
rs554219	2.37E-02	5.46E-01	3.47E-01	1.88E-04	6.35E-01	9.19E-01
rs78540526	1.05E-02	7.58E-01	4.81E-01	3.17E-04	9.63E-01	3.42E-01
rs11820646	4.59E-01	9.31E-01	3.49E-01	1.66E-02	7.93E-02	8.11E-01
rs12422552	1.52E-02	4.32E-01	8.87E-01	4.54E-05	2.96E-02	2.14E-01
rs10771399	9.98E-04	3.04E-01	2.42E-01	2.70E-05	2.76E-04	4.14E-01
rs17356907	1.49E-01	2.22E-02	2.34E-01	3.80E-03	8.29E-03	6.77E-01
rs1292011	1.62E-01	3.31E-01	4.65E-01	2.83E-01	3.78E-02	2.80E-02
rs11571833	2.74E-03	1.68E-01	9.38E-01	2.77E-05	3.73E-03	8.17E-01
rs2236007	3.73E-02	2.02E-01	4.97E-01	1.68E-04	3.61E-01	6.16E-01
rs2588809	1.60E-01	6.28E-01	6.01E-01	1.86E-01	9.71E-01	9.55E-01
rs999737	1.77E-01	1.10E-02	8.08E-02	7.01E-04	6.64E-02	9.94E-01
rs11627032	1.57E-02	4.18E-01	7.77E-01	2.72E-03	2.35E-02	8.46E-01
rs941764	3.77E-02	5.87E-01	9.17E-01	4.31E-03	2.72E-01	8.47E-01
rs11075995	4.96E-01	3.89E-02	6.49E-01	4.55E-02	2.73E-03	6.68E-01
rs17817449	1.14E-02	2.26E-01	5.06E-01	6.03E-04	2.50E-03	4.03E-01
rs3803662	9.13E-01	2.51E-04	1.54E-01	5.85E-01	4.80E-08	2.17E-01
rs13329835	1.74E-03	1.50E-01	2.49E-01	9.47E-05	2.05E-01	4.19E-01
chr17.29230520.D	4.38E-02	1.62E-01	5.31E-01	2.64E-03	4.21E-02	5.45E-01
rs6504950	3.45E-03	7.09E-01	3.42E-01	7.26E-04	3.92E-01	8.12E-01
rs745570	7.33E-02	3.06E-01	9.57E-01	1.60E-02	2.76E-02	9.51E-01
rs1436904	7.21E-02	7.68E-01	7.84E-01	3.83E-02	4.53E-01	3.24E-01
rs527616	2.06E-03	3.16E-02	6.64E-02	4.33E-05	1.29E-02	2.14E-02
rs6507583	9.64E-03	3.59E-01	5.11E-01	3.01E-05	7.96E-01	6.12E-01
rs2363956	5.92E-03	2.44E-01	2.18E-01	9.78E-04	1.45E-04	2.89E-01
rs4808801	6.38E-03	4.25E-01	4.01E-01	1.04E-03	4.25E-03	6.92E-01
rs8170	2.75E-02	1.40E-01	5.77E-01	3.81E-02	2.99E-02	1.16E-02
rs3760982	1.08E-01	4.14E-01	9.22E-01	5.62E-04	5.82E-01	1.94E-01
rs2823093	3.77E-02	4.20E-01	6.98E-01	2.07E-04	7.05E-01	4.83E-01
rs132390	6.97E-01	5.33E-01	9.77E-01	9.00E-01	9.03E-02	4.74E-01
rs17879961	2.65E-03	6.74E-01	7.31E-01	3.47E-05	3.09E-01	2.30E-01
rs6001930	1.32E-02	4.42E-01	5.36E-01	7.12E-04	5.41E-02	3.11E-01

Adj: adjusted

Supplementary Table 15. Associations with ER-negative breast cancer for SNPs ($p<1x10^{-6}$) in the 2q33 region.

					iCOGS/GWAS ER-negative			*BRCA1* carriers			Meta
**Chr**	**Position**	**rs#**	**Allele1**	**Allele2**	**MAF**	**OR**	**P-value**	**MAF**	**HR**	**P-value**	**P-value[3]**
2	201717014	rs74943274	G	A	0.015	1.34	$5.89x10^{-6}$	0.017	1.20	0.020	$6.00x10^{-7}$
2	201717290	rs78529534	A	G	0.016	0.75	$5.13x10^{-6}$	0.017	1.23	0.008	$1.83x10^{-7}$
2	201723140	rs79751905	G	A	0.015	1.34	$4.83x10^{-6}$	0.016	1.20	0.020	$5.02x10^{-7}$
2	201723695	rs150365829	C	T	0.015	1.34	$4.61x10^{-6}$	0.016	1.20	0.020	$4.92x10^{-7}$
2	201723902	rs13415568	C	G	0.015	0.75	$4.26x10^{-6}$	0.017	1.23	0.009	$1.78x10^{-7}$
2	201724391	rs41271455	T	C	0.015	0.74	$4.01x10^{-6}$	0.016	1.20	0.020	$4.39x10^{-7}$
2	201726390	rs17467658	T	C	0.015	0.74	$2.68x10^{-6}$	0.017	1.20	0.020	$3.18x10^{-7}$
2	201729335	rs78258606	A	G	0.015	0.74	$1.23x10^{-6}$	0.017	1.20	0.023	$1.91x10^{-7}$
2	201731754	chr2:201731754:D	CCAAA	C	0.015	0.74	$1.76x10^{-6}$	0.017	1.21	0.018	$1.82x10^{-7}$
2	201732571	rs149780988	T	C	0.015	0.74	$1.61x10^{-6}$	0.017	1.21	0.018	$1.70x10^{-7}$
2	201733341	rs188686860	C	T	0.016	1.36	$1.16x10^{-6}$	0.017	1.22	0.013	$8.34x10^{-8}$
2	201733955	rs145445307	T	C	0.015	0.74	$1.54x10^{-6}$	0.017	1.21	0.018	$1.67x10^{-7}$
2	201739884	rs17383151	T	C	0.015	0.74	$1.50x10^{-6}$	0.017	1.21	0.017	$1.60x10^{-7}$
2	201743594	rs115635831	A	G	0.015	0.73	$1.07x10^{-6}$	0.017	1.21	0.018	$1.26x10^{-7}$
2	201749127	rs139580051	A	C	0.015	0.74	$1.76x10^{-6}$	0.017	1.21	0.015	$1.56x10^{-7}$
2	201750683	rs16835425	C	T	0.015	1.35	$2.61x10^{-6}$	0.017	1.20	0.019	$2.77x10^{-7}$
2	201773257	rs114687445	A	G	0.015	0.74	$1.22x10^{-6}$	0.017	1.21	0.015	$1.03x10^{-7}$
2	201778911	rs192952120	C	T	0.015	1.36	$9.94x10^{-7}$	0.017	1.21	0.015	$8.66x10^{-8}$
2	201779820	rs190521476	C	A	0.016	1.36	$1.15x10^{-6}$	0.017	1.21	0.015	$1.04x10^{-7}$
2	201782763	rs17467748	A	G	0.015	0.74	$9.93x10^{-7}$	0.017	1.21	0.015	$8.66x10^{-8}$
2	201786050	chr2:201786050:I	G	GT	0.015	0.74	$9.93x10^{-7}$	0.017	1.21	0.015	$8.66x10^{-8}$
2	201787453	rs16835746	C	T	0.015	1.36	$9.93x10^{-7}$	0.017	1.21	0.015	$8.66x10^{-8}$
2	201788479	rs76637130	A	C	0.016	0.75	$4.47x10^{-6}$	0.018	1.19	0.023	$5.70x10^{-7}$
2	201789600	rs16835779	C	G	0.016	0.75	$5.34x10^{-6}$	0.018	1.19	0.023	$6.60x10^{-7}$
2	201789638	rs148850748	C	T	0.015	1.36	$9.93x10^{-7}$	0.017	1.21	0.015	$8.66x10^{-8}$
2	201793388	rs183216043	T	C	0.015	0.74	$9.93x10^{-7}$	0.017	1.21	0.015	$8.66x10^{-8}$
2	201794885	rs17383256	G	C	0.015	1.36	$9.89x10^{-7}$	0.017	1.21	0.015	$8.60x10^{-8}$
2	201800167	rs11895356	G	A	0.016	1.33	$4.85x10^{-6}$	0.018	1.20	0.021	$5.41x10^{-7}$
2	201800686	rs16835997	A	T	0.015	0.74	$9.92x10^{-7}$	0.017	1.21	0.015	$8.66x10^{-8}$
2	201804562	rs16836061	A	C	0.015	0.74	$9.92x10^{-7}$	0.017	1.21	0.015	$8.66x10^{-8}$
2	201805255	chr2:201805255:I	T	TG	0.015	0.74	$9.92x10^{-7}$	0.017	1.21	0.015	$8.76x10^{-8}$
2	201813224	rs16836158	A	G	0.016	0.75	$5.73x10^{-6}$	0.018	1.19	0.023	$7.01x10^{-7}$

2	201816239	rs75490883	T	C	0.016	0.75	$5.44x10^{-6}$	0.018	1.19	0.023	$6.67x10^{-7}$
2	201819228	rs17383298	A	G	0.015	0.74	$1.93x10^{-6}$	0.016	1.22	0.015	$1.58x10^{-7}$
2	201821221	rs112281084	A	G	0.015	0.74	$1.21x10^{-6}$	0.017	1.21	0.015	$1.02x10^{-7}$
2	201837772	rs11899179	G	A	0.016	1.33	$4.49x10^{-6}$	0.017	1.20	0.019	$4.48x10^{-7}$
2	201840567	chr2:201840567:D	TGG	T	0.016	1.36	$1.21x10^{-6}$	0.017	1.21	0.015	$1.02x10^{-7}$
2	201842450	rs142608159	T	C	0.015	0.74	$1.21x10^{-6}$	0.017	1.21	0.015	$1.02x10^{-7}$
2	201846083	rs17467902	G	A	0.016	1.36	$1.21x10^{-6}$	0.017	1.21	0.015	$1.02x10^{-7}$
2	201847573	rs148420167	C	T	0.016	1.36	$1.15x10^{-6}$	0.017	1.21	0.014	$9.70x10^{-8}$
2	201847877	rs11895568	A	G	0.016	0.75	$4.24x10^{-6}$	0.017	1.20	0.019	$4.28x10^{-7}$
2	201850107	rs144999911	G	A	0.016	1.36	$1.21x10^{-6}$	0.017	1.21	0.015	$1.01x10^{-7}$
2	201852394	rs115331342	C	A	0.016	1.36	$1.20x10^{-6}$	0.017	1.21	0.015	$1.01x10^{-7}$
2	201854025	rs17383382	T	C	0.015	0.74	$1.20x10^{-6}$	0.017	1.21	0.015	$1.01x10^{-7}$
2	201854462	rs80211752	G	A	0.016	1.36	$1.20x10^{-6}$	0.017	1.21	0.015	$1.01x10^{-7}$
2	201855198	rs11893239	T	C	0.016	0.75	$4.05x10^{-6}$	0.017	1.20	0.019	$4.11x10^{-7}$
2	201857829	rs56911059	A	T	0.016	0.75	$3.99x10^{-6}$	0.017	1.20	0.019	$4.06x10^{-7}$
2	201858087	rs17467916	T	C	0.016	0.74	$1.48x10^{-6}$	0.017	1.21	0.015	$1.24x10^{-7}$
2	201858344	rs75853971	A	T	0.015	0.74	$1.20x10^{-6}$	0.017	1.21	0.015	$1.01x10^{-7}$
2	201862428	rs16836506	A	T	0.016	0.75	$3.89x10^{-6}$	0.017	1.20	0.019	$3.98x10^{-7}$
2	201863860	rs146820497	C	A	0.016	1.36	$1.20x10^{-6}$	0.017	1.21	0.015	$1.01x10^{-7}$
2	201865559	chr2:201865559:D	AG	A	0.016	1.33	$3.62x10^{-6}$	0.017	1.20	0.019	$3.75x10^{-7}$
2	201865895	rs116700774	G	A	0.016	1.36	$1.19x10^{-6}$	0.017	1.21	0.015	$1.00x10^{-7}$
2	201867467	rs116539488	C	T	0.016	1.36	$1.19x10^{-6}$	0.017	1.21	0.015	$1.01x10^{-7}$
2	201869491	rs11900960	G	A	0.016	1.34	$3.17x10^{-6}$	0.017	1.20	0.019	$3.33x10^{-7}$
2	201870021	rs114567273	G	A	0.016	1.36	$1.19x10^{-6}$	0.017	1.21	0.015	$1.00x10^{-7}$
2	201870282	rs76520541	A	G	0.016	0.75	$3.41x10^{-6}$	0.017	1.20	0.019	$3.55x10^{-7}$
2	201870798	rs190565001	G	A	0.019	1.32	$4.12x10^{-6}$	0.021	1.20	0.015	$3.02x10^{-7}$
2	201871575	rs147647393	C	A	0.016	1.36	$1.19x10^{-6}$	0.017	1.21	0.015	$1.00x10^{-7}$
2	201872180	rs79395719	T	G	0.016	0.75	$3.16x10^{-6}$	0.017	1.20	0.019	$3.29x10^{-7}$
2	201875490	chr2:201875490:I	A	AG	0.016	0.75	$3.32x10^{-6}$	0.017	1.20	0.019	$3.48x10^{-7}$
2	201876052	rs16836551	C	T	0.016	1.34	$3.31x10^{-6}$	0.017	1.20	0.019	$3.46x10^{-7}$
2	201876969	rs151067359	G	C	0.016	1.36	$1.18x10^{-6}$	0.017	1.21	0.015	$1.00x10^{-7}$
2	201879455	rs11892279	T	C	0.016	0.75	$3.22x10^{-6}$	0.017	1.20	0.019	$3.39x10^{-7}$
2	201880105	chr2:201880105:I	T	TTTC	0.016	0.75	$2.76x10^{-6}$	0.017	1.20	0.019	$2.94x10^{-7}$
2	201886358	rs16836588	C	A	0.017	1.33	$3.69x10^{-6}$	0.018	1.21	0.015	$2.83x10^{-7}$
2	201890604	rs114008573	C	T	0.016	1.36	$1.21x10^{-6}$	0.017	1.21	0.015	$1.02x10^{-7}$
2	201892188	rs150638321	G	A	0.016	1.34	$3.18x10^{-6}$	0.017	1.20	0.019	$3.35x10^{-7}$

2	201892272	rs189252016	G	A	0.016	1.36	1.04×10^{-6}	0.017	1.21	0.015	8.99×10^{-8}
2	201892855	rs149765952	C	T	0.016	1.36	1.17×10^{-6}	0.017	1.21	0.015	9.87×10^{-8}
2	201893802	rs144069676	G	C	0.016	1.35	1.45×10^{-6}	0.017	1.21	0.018	1.59×10^{-7}
2	201895262	rs138504123	A	G	0.015	0.74	1.17×10^{-6}	0.017	1.21	0.015	9.87×10^{-8}
2	201897787	rs114326395	C	A	0.016	1.36	1.16×10^{-6}	0.017	1.21	0.015	9.80×10^{-8}
2	201898427	rs75246650	C	T	0.016	1.34	3.31×10^{-6}	0.017	1.20	0.019	3.50×10^{-7}
2	201898885	chr2:201898885:I	A	ACAT	0.016	0.75	3.03×10^{-6}	0.017	1.20	0.019	3.20×10^{-7}
2	201898888	chr2:201898888:I	T	TC	0.016	0.75	3.18×10^{-6}	0.017	1.20	0.019	3.35×10^{-7}
2	201899731	rs146272565	G	A	0.016	1.36	1.17×10^{-6}	0.017	1.21	0.015	9.87×10^{-8}
2	201901974	rs111564182	A	C	0.016	0.75	3.18×10^{-6}	0.017	1.20	0.019	3.33×10^{-7}
2	201902058	chr2:201902058:D	CA	C	0.015	0.74	1.17×10^{-6}	0.017	1.21	0.015	9.87×10^{-8}
2	201903323	rs184264743	G	A	0.016	1.36	1.17×10^{-6}	0.017	1.21	0.015	9.87×10^{-8}
2	201903324	rs186994307	G	A	0.016	1.36	1.17×10^{-6}	0.017	1.21	0.015	9.87×10^{-8}
2	201903636	rs116313342	T	A	0.016	1.33	3.90×10^{-6}	0.018	1.19	0.023	5.04×10^{-7}
2	201908835	rs114699270	A	G	0.015	0.74	1.15×10^{-6}	0.017	1.21	0.015	1.01×10^{-7}
2	201910772	rs116089517	A	G	0.015	0.74	1.15×10^{-6}	0.017	1.21	0.015	1.01×10^{-7}
2	201911085	rs116309768	C	A	0.016	1.36	1.15×10^{-6}	0.017	1.21	0.015	1.01×10^{-7}
2	201911475	rs116186593	T	G	0.015	0.74	1.15×10^{-6}	0.017	1.21	0.015	1.01×10^{-7}
2	201913284	rs60630751	A	G	0.016	0.75	2.94×10^{-6}	0.017	1.20	0.019	3.13×10^{-7}
2	201914166	rs116416403	G	A	0.016	1.36	1.15×10^{-6}	0.017	1.21	0.015	1.01×10^{-7}
2	201914847	rs116276983	G	A	0.016	1.36	1.15×10^{-6}	0.017	1.21	0.015	1.01×10^{-7}
2	201916833	rs115928143	C	T	0.016	1.36	1.15×10^{-6}	0.017	1.21	0.015	1.01×10^{-7}
2	201919002	rs116500747	C	T	0.016	1.36	1.15×10^{-6}	0.017	1.21	0.015	1.00×10^{-7}
2	201919375	rs75140790	T	C	0.015	0.74	1.15×10^{-6}	0.017	1.21	0.015	1.00×10^{-7}
2	201919563	rs150173964	T	G	0.015	0.74	1.15×10^{-6}	0.017	1.21	0.015	1.00×10^{-7}
2	201920278	rs116301213	A	T	0.016	0.75	3.17×10^{-6}	0.017	1.20	0.019	3.35×10^{-7}
2	201920924	chr2:201920924:I	A	AAAATAAAT	0.016	0.74	2.04×10^{-6}	0.017	1.21	0.017	2.01×10^{-7}
2	201921441	rs115167130	T	G	0.016	0.75	3.16×10^{-6}	0.017	1.20	0.019	3.35×10^{-7}
2	201922345	rs145748840	G	A	0.016	1.36	1.15×10^{-6}	0.017	1.21	0.015	1.00×10^{-7}
2	201923157	rs116478550	A	G	0.016	0.75	3.15×10^{-6}	0.017	1.20	0.019	3.35×10^{-7}
2	201923635	rs74596795	C	T	0.016	1.34	2.39×10^{-6}	0.018	1.19	0.026	3.83×10^{-7}
2	201924358	rs58911199	G	T	0.016	1.34	3.14×10^{-6}	0.017	1.20	0.019	3.33×10^{-7}
2	201924449	rs58669897	G	C	0.016	1.34	3.14×10^{-6}	0.017	1.20	0.019	3.33×10^{-7}
2	201924488	rs116699946	A	G	0.016	0.75	3.14×10^{-6}	0.017	1.20	0.019	3.33×10^{-7}
2	201924933	rs76377168	G	A	0.016	1.34	3.14×10^{-6}	0.017	1.20	0.019	3.33×10^{-7}
2	201927809	rs181144634	G	A	0.016	1.36	1.14×10^{-6}	0.017	1.21	0.015	9.97×10^{-8}

2	201927927	rs145216595	C	T	0.016	1.36	$1.14x10^{-6}$	0.017	1.21	0.015	$9.97x10^{-8}$
2	201928037	rs142340339	C	T	0.016	1.36	$1.14x10^{-6}$	0.017	1.21	0.015	$9.97x10^{-8}$
2	201929766	rs74325135	G	T	0.016	1.34	$3.08x10^{-6}$	0.017	1.20	0.020	$3.42x10^{-7}$
2	201931008	rs148220417	G	A	0.016	1.36	$1.14x10^{-6}$	0.017	1.21	0.015	$9.97x10^{-8}$
2	201931057	rs141180406	G	A	0.019	1.30	$8.81x10^{-6}$	0.021	1.19	0.016	$6.55x10^{-7}$
2	201931200	chr2:201931200:D	TAATA	T	0.016	1.36	$1.14x10^{-6}$	0.017	1.21	0.015	$9.97x10^{-8}$
2	201931610	rs78519396	T	C	0.016	0.75	$3.13x10^{-6}$	0.017	1.20	0.020	$3.50x10^{-7}$
2	201933121	rs16836607	G	A	0.016	1.33	$3.90x10^{-6}$	0.017	1.20	0.020	$4.25x10^{-7}$
2	201935861	rs116198267	G	A	0.016	1.36	$1.27x10^{-6}$	0.017	1.21	0.015	$1.06x10^{-7}$
2	201935871	rs114962751	T	A	0.016	1.36	$1.17x10^{-6}$	0.017	1.22	0.011	$7.24x10^{-8}$
2	201937599	rs114710676	T	C	0.015	0.74	$1.27x10^{-6}$	0.017	1.21	0.015	$1.06x10^{-7}$
2	201938297	rs113072211	C	G	0.015	0.74	$1.33x10^{-6}$	0.017	1.21	0.015	$1.11x10^{-7}$
2	201938442	rs142246292	C	G	0.016	0.75	$2.52x10^{-6}$	0.017	1.20	0.020	$2.88x10^{-7}$
2	201938503	rs183774255	A	T	0.015	0.74	$1.27x10^{-6}$	0.017	1.21	0.015	$1.06x10^{-7}$
2	201939858	chr2:201939858:D	CAGG	C	0.015	0.74	$1.27x10^{-6}$	0.017	1.21	0.015	$1.06x10^{-7}$
2	201939958	rs116739540	T	C	0.015	0.74	$1.17x10^{-6}$	0.017	1.21	0.015	$9.87x10^{-8}$
2	201941977	rs11896935	G	T	0.016	1.34	$2.51x10^{-6}$	0.017	1.20	0.020	$2.88x10^{-7}$
2	201942229	rs115300559	G	A	0.016	1.35	$1.33x10^{-6}$	0.017	1.21	0.015	$1.11x10^{-7}$
2	201942367	rs11897109	G	A	0.016	1.34	$2.51x10^{-6}$	0.017	1.20	0.020	$2.86x10^{-7}$
2	201942774	rs139270392	C	T	0.016	1.35	$1.33x10^{-6}$	0.017	1.21	0.015	$1.11x10^{-7}$
2	201943154	rs76655038	C	T	0.016	1.35	$1.33x10^{-6}$	0.017	1.21	0.015	$1.11x10^{-7}$
2	201943431	rs11887751	A	G	0.016	0.75	$2.51x10^{-6}$	0.017	1.20	0.020	$2.86x10^{-7}$
2	201943996	rs144629284	C	T	0.016	1.36	$1.16x10^{-6}$	0.017	1.21	0.016	$1.10x10^{-7}$
2	201945722	rs116331034	A	G	0.016	0.75	$2.51x10^{-6}$	0.017	1.20	0.020	$2.86x10^{-7}$
2	201946829	rs112065557	G	A	0.016	1.35	$1.33x10^{-6}$	0.017	1.21	0.015	$1.11x10^{-7}$
2	201946957	rs11888623	T	G	0.016	0.75	$2.51x10^{-6}$	0.017	1.20	0.020	$2.86x10^{-7}$
2	201947403	rs11888847	T	C	0.016	0.75	$2.52x10^{-6}$	0.018	1.21	0.015	$2.01x10^{-7}$
2	201947732	rs149206908	G	A	0.016	1.36	$1.22x10^{-6}$	0.017	1.21	0.015	$1.02x10^{-7}$
2	201949194	rs141852703	C	G	0.015	0.74	$1.16x10^{-6}$	0.017	1.21	0.015	$9.83x10^{-8}$
2	201950540	rs111978201	T	C	0.015	0.74	$1.27x10^{-6}$	0.017	1.21	0.015	$1.07x10^{-7}$
2	201951530	chr2:201951530:I	C	CT	0.016	1.36	$1.27x10^{-6}$	0.017	1.21	0.015	$1.07x10^{-7}$
2	201954611	rs76260028	G	A	0.016	1.34	$2.58x10^{-6}$	0.017	1.20	0.020	$3.00x10^{-7}$
2	201958283	rs115144903	A	G	0.015	0.74	$1.34x10^{-6}$	0.017	1.21	0.015	$1.15x10^{-7}$
2	201958437	rs116509920	T	C	0.015	0.74	$1.16x10^{-6}$	0.017	1.21	0.015	$1.00x10^{-7}$
2	201958962	rs116230086	C	G	0.016	0.75	$2.63x10^{-6}$	0.017	1.20	0.021	$3.10x10^{-7}$
2	201961225	rs138763733	C	T	0.016	1.36	$1.11x10^{-6}$	0.017	1.21	0.015	$1.01x10^{-7}$

2	201961493	rs148040754	C	T	0.016	1.36	1.12×10^{-6}	0.017	1.21	0.015	1.01×10^{-7}
2	201962452	rs139445865	T	C	0.015	0.74	1.12×10^{-6}	0.017	1.21	0.015	1.02×10^{-7}
2	201963070	rs116313702	A	G	0.015	0.73	1.81×10^{-6}	0.015	1.20	0.037	4.30×10^{-7}
2	201964642	rs17383533	A	T	0.015	0.74	1.12×10^{-6}	0.017	1.21	0.015	1.02×10^{-7}
2	201966510	chr2:201966510:D	GCT	G	0.016	1.36	1.12×10^{-6}	0.017	1.21	0.015	9.90×10^{-8}
2	201969453	rs114176379	A	C	0.015	0.74	1.12×10^{-6}	0.017	1.21	0.015	1.01×10^{-7}
2	201971529	rs138875581	G	C	0.016	1.36	1.12×10^{-6}	0.017	1.21	0.016	1.04×10^{-7}
2	201971887	rs75196967	G	A	0.016	1.36	1.17×10^{-6}	0.017	1.21	0.016	1.08×10^{-7}
2	201973150	chr2:201973150:D	ATC	A	0.016	1.36	1.11×10^{-6}	0.017	1.21	0.016	1.04×10^{-7}
2	201973547	rs149458356	G	A	0.016	1.36	1.11×10^{-6}	0.017	1.21	0.016	1.05×10^{-7}
2	201974896	rs139722324	G	A	0.016	1.36	1.10×10^{-6}	0.017	1.21	0.016	1.05×10^{-7}
2	201976712	rs114247218	C	T	0.016	1.36	1.09×10^{-6}	0.017	1.21	0.016	1.05×10^{-7}
2	201978027	rs74482315	G	T	0.016	1.34	2.23×10^{-6}	0.018	1.19	0.027	3.77×10^{-7}
2	201980843	rs75208666	G	A	0.016	1.36	1.08×10^{-6}	0.017	1.21	0.017	1.07×10^{-7}
2	201994992	rs116782073	T	C	0.015	0.73	7.73×10^{-7}	0.017	1.20	0.020	1.04×10^{-7}
2	201995639	rs79959919	T	G	0.016	0.74	1.97×10^{-6}	0.017	1.20	0.022	2.57×10^{-7}
2	201995860	rs114984720	C	T	0.016	1.36	1.21×10^{-6}	0.017	1.21	0.018	1.31×10^{-7}
2	202002617	rs116724456	C	G	0.015	0.74	1.21×10^{-6}	0.017	1.21	0.018	1.37×10^{-7}
2	202003665	rs149336947	G	A	0.016	1.36	1.21×10^{-6}	0.017	1.20	0.019	1.39×10^{-7}
2	202006755	rs143709332	T	C	0.015	0.74	1.24×10^{-6}	0.017	1.20	0.019	1.42×10^{-7}
2	202019882	rs149297763	G	A	0.016	1.36	1.20×10^{-6}	0.017	1.20	0.019	1.46×10^{-7}
2	202027036	rs189192934	A	C	0.015	0.74	1.19×10^{-6}	0.017	1.20	0.020	1.52×10^{-7}
2	202032688	rs112762622	C	T	0.016	1.35	1.59×10^{-6}	0.018	1.19	0.027	2.97×10^{-7}
2	202034497	rs137937873	C	T	0.016	1.35	1.30×10^{-6}	0.017	1.20	0.021	1.74×10^{-7}
2	202096254	rs17860411	C	T	0.015	1.40	3.99×10^{-7}	0.015	1.15	0.112	5.93×10^{-7}
2	202112571	rs147538031	T	G	0.015	0.72	4.10×10^{-7}	0.017	1.13	0.135	9.72×10^{-7}

P-values for BRCA1 carriers were estimated by a kinship-adjusted retrospective likelihood approach.
P-values for iCOGS/BCAC and for meta-analysis were estimated by Chi-square

Supplementary Table 16. Conditional analysis for index SNPs from five independent signals in the 2q33.1 *PPIL3/CASP8* region

				**Overall breast cancer**				**ER-negative breast cancer**			
				Adjusted		Unadjusted		Adjusted		Unadjusted	
Location	Position	*Gene*	rs#	OR	P	OR	P	OR	P	OR	P
2q33.1	201717014	*CLK1*	rs74943274	1.14	$1.44x10^{-3}$	1.13	$1.2x10^{-3}$	1.38	$1.35x10^{-5}$	1.35	$1.10x10^{-5}$
2q33.1	202036478	*CFLAR*	rs7558475	1.06	$4.24x10^{-3}$	1.07	$2.4x10^{-4}$	1.08	0.04	1.09	0.013
2q33.1	202141838	*CASP8*	rs36043647	0.98	0.26	0.97	0.17	0.96	0.28	0.95	0.19
2q33.1	202181247	*ALS2CR12*	rs1830298	0.97	$3.71x10^{-3}$	0.96	$2.40x10^{-5}$	0.98	0.32	0.96	0.083
2q33.1	202379828	*ALS2CR11*	rs59278883	0.94	$3.04x10^{-3}$	0.94	$1.4x10^{-4}$	0.94	0.09	0.94	0.068

iCOGS/BCAC data only. Associations for each index SNP were adjusted for the four other index SNPs.
Adjusted: Associations for each index SNP adjusted for the four other index SNPs.
Unadjusted: Associations for each index SNP without adjustment
P-values for iCOGS/BCAC and for meta-analysis were estimated by Chi-square

Supplemental Table 17. 2p23 eQTL SNPs from BC765 and BC241 tumor cohorts

**eQTL BC765**						
**Gene**	**ePos**	**SNP**	**mPos**	**N**	**p-value**	**ER-neg p-value**
TRMT61B	29082931	rs6419696	29010339	762	1.2E-17	0.0026
TRMT61B	29082931	rs13016112	29012574	757	1.5E-17	0.0027
TRMT61B	29082931	chr2:29062249:D	29062249	753	1.8E-17	0.0022
TRMT61B	29082931	rs3190	29025479	754	2.0E-17	0.0045
TRMT61B	29082931	chr2:29019056:D	29019056	757	2.6E-17	0.0024
TRMT61B	29082931	rs4132617	29031312	763	4.4E-17	0.0029
TRMT61B	29082931	chr2:29021511:D	29021511	761	5.1E-17	0.0037
TRMT61B	29082931	rs6547873	28981983	756	6.5E-17	0.0065
TRMT61B	29082931	rs6547874	28982030	756	6.5E-17	0.0065
TRMT61B	29082931	rs7579321	28983166	756	6.5E-17	0.0063
TRMT61B	29082931	chr2:29021814:D	29021814	759	8.8E-17	0.0027
TRMT61B	29082931	rs4563180	29093695	754	9.7E-17	0.002
TRMT61B	29082931	rs4233729	29092679	756	1.1E-16	0.0023
TRMT61B	29082931	rs4611608	29064536	765	1.1E-16	0.0023
TRMT61B	29082931	rs4666122	29092850	755	1.1E-16	0.0023
TRMT61B	29082931	rs4638745	29093803	755	1.1E-16	0.0023
TRMT61B	29082931	rs7567861	29035849	759	1.2E-16	0.0027
TRMT61B	29082931	rs6709674	28989667	749	1.3E-16	0.0063
TRMT61B	29082931	rs4665436	29090227	757	1.3E-16	0.0023
TRMT61B	29082931	rs6547880	29018889	758	1.4E-16	0.0027
TRMT61B	29082931	rs11127189	28992020	754	1.7E-16	0.0057
TRMT61B	29082931	rs12475612	29030006	762	1.9E-16	0.0028
TRMT61B	29082931	rs6718662	29048758	757	2.0E-16	0.0025
TRMT61B	29082931	rs56178008	29098543	752	2.1E-16	0.0035
TRMT61B	29082931	SNP_A-8465813	28998077	765	2.1E-16	0.00075
TRMT61B	29082931	SNP_A-1900844	29061111	765	2.2E-16	0.0022
TRMT61B	29082931	rs6547878	29010066	758	2.3E-16	0.00077
TRMT61B	29082931	rs6747852	29009840	760	2.3E-16	0.00064
TRMT61B	29082931	rs6742291	29007349	761	2.3E-16	0.00064
TRMT61B	29082931	rs72784066	29081175	758	2.3E-16	0.0025
TRMT61B	29082931	rs1128416	29001691	760	2.4E-16	0.00069
TRMT61B	29082931	rs11684695	29088450	765	2.5E-16	0.0022
TRMT61B	29082931	rs6547882	29037001	750	2.7E-16	0.0021
TRMT61B	29082931	rs12714241	28969413	749	2.8E-16	0.01
TRMT61B	29082931	rs66904522	29080892	762	3.0E-16	0.0019
TRMT61B	29082931	rs10865508	29053704	762	3.2E-16	0.0025
TRMT61B	29082931	rs11685682	29056801	762	3.2E-16	0.0024
TRMT61B	29082931	rs6547877	29006591	724	4.3E-16	0.00023
TRMT61B	29082931	rs55785599	29087814	764	4.7E-16	0.002
TRMT61B	29082931	rs3768665	29005602	761	5.1E-16	0.00065
TRMT61B	29082931	chr2:28994282:I	28994282	758	5.6E-16	0.00088

TRMT61B	29082931	rs11127190	29043212	758	5.7E-16	0.0028
TRMT61B	29082931	rs67410623	29057440	761	6.0E-16	0.0023
TRMT61B	29082931	rs10174533	28968928	749	6.3E-16	0.012
TRMT61B	29082931	rs966250	28974139	749	6.3E-16	0.012
TRMT61B	29082931	chr2:29094722:I	29094722	742	6.6E-16	0.0019
TRMT61B	29082931	rs57286839	29084934	750	7.4E-16	0.0023
TRMT61B	29082931	rs4632298	29076282	756	8.4E-16	0.0022
TRMT61B	29082931	rs11686412	28992405	756	8.6E-16	0.00052
TRMT61B	29082931	rs12472549	28992004	755	8.7E-16	0.00054
TRMT61B	29082931	rs10198789	28968811	747	9.5E-16	0.012
TRMT61B	29082931	chr2:29007808:D	29007808	759	1.0E-15	0.00048
TRMT61B	29082931	chr2:29062248:D	29062248	713	1.3E-15	0.0038
TRMT61B	29082931	rs62129843	29008175	712	1.4E-15	0.00055
TRMT61B	29082931	rs4477866	29009089	749	1.9E-15	0.0042
TRMT61B	29082931	rs6710080	28989975	752	2.2E-15	0.00078
TRMT61B	29082931	rs6710082	28989977	752	2.2E-15	0.00087
TRMT61B	29082931	rs6706858	28992607	753	2.5E-15	0.00057
TRMT61B	29082931	chr2:28993652:D	28993652	749	6.0E-15	0.00053
TRMT61B	29082931	rs12714242	28982629	730	6.7E-15	0.0098
TRMT61B	29082931	rs4632297	29008425	747	2.8E-14	0.0011
TRMT61B	29082931	rs78719787	28983991	729	3.1E-14	0.5
TRMT61B	29082931	chr2:28994284:I	28994284	694	1.9E-13	7.00E-04
TRMT61B	29082931	chr2:29101401:D	29101401	713	8.9E-12	0.011
TRMT61B	29082931	rs4358081	29100642	764	2.8E-11	0.016
TRMT61B	29082931	rs4289133	29100646	764	2.9E-11	0.016
TRMT61B	29082931	SNP_A-8408952	29100845	765	5.8E-11	0.016
TRMT61B	29082931	rs11690571	29075031	738	1.1E-10	0.0038
TRMT61B	29082931	rs4666119	28975690	755	3.1E-10	0.034
TRMT61B	29082931	rs6707848	28988083	752	8.2E-10	0.025
TRMT61B	29082931	rs4666124	29102106	739	1.1E-09	0.0054
TRMT61B	29082931	rs7579277	28996295	751	1.1E-09	0.014
TRMT61B	29082931	rs6752464	28989887	747	1.2E-09	0.013
TRMT61B	29082931	chr2:29003787:D	29003787	744	1.5E-09	0.0096
TRMT61B	29082931	rs12714246	29017358	761	1.6E-09	0.0086
TRMT61B	29082931	rs10183665	28995226	759	1.7E-09	0.013
TRMT61B	29082931	chr2:29016262:D	29016262	759	2.0E-09	0.0099
TRMT61B	29082931	rs6730321	29004299	763	2.0E-09	0.012
TRMT61B	29082931	rs12714243	28992293	755	2.1E-09	0.013
TRMT61B	29082931	rs13018031	29015982	763	2.1E-09	0.011
TRMT61B	29082931	rs2276547	28975107	719	2.1E-09	0.05
TRMT61B	29082931	rs4665432	28952026	764	2.3E-09	0.036
TRMT61B	29082931	rs11688129	28952581	764	2.3E-09	0.041
TRMT61B	29082931	rs4549034	28972579	764	2.3E-09	0.035
TRMT61B	29082931	rs4372836	28973883	764	2.3E-09	0.035

TRMT61B	29082931	chr2:28986568:D	28986568	733	2.4E-09	0.015
TRMT61B	29082931	rs2045886	29010517	764	2.4E-09	0.011
TRMT61B	29082931	chr2:29011141:D	29011141	764	2.4E-09	0.012
TRMT61B	29082931	rs13011935	29014746	764	2.4E-09	0.012
TRMT61B	29082931	rs7475	29023749	764	2.4E-09	0.011
TRMT61B	29082931	rs6547872	28978074	747	2.6E-09	0.076
TRMT61B	29082931	SNP_A-1908574	29005870	765	2.8E-09	0.011
TRMT61B	29082931	chr2:28955848:I	28955848	758	3.2E-09	0.04
TRMT61B	29082931	rs12185717	29069551	756	3.3E-09	0.013
TRMT61B	29082931	chr2:28951498:D	28951498	761	3.3E-09	0.036
TRMT61B	29082931	rs4666123	29102004	760	3.5E-09	0.0064
TRMT61B	29082931	rs4254466	29009049	727	3.5E-09	0.0049
TRMT61B	29082931	SNP_A-2279820	28951698	765	3.8E-09	0.036
TRMT61B	29082931	rs12465314	29102888	758	3.8E-09	0.0069
TRMT61B	29082931	rs55721532	28938968	765	3.9E-09	0.027
TRMT61B	29082931	rs4408686	28940526	765	3.9E-09	0.027
TRMT61B	29082931	rs12151717	29004046	764	4.1E-09	0.01
TRMT61B	29082931	rs6707130	28973276	764	4.6E-09	0.043
TRMT61B	29082931	rs11127188	28972852	764	4.7E-09	0.039
TRMT61B	29082931	rs62131973	29044437	758	4.9E-09	0.012
TRMT61B	29082931	rs13024443	29046185	758	4.9E-09	0.012
TRMT61B	29082931	rs4665431	28938168	764	5.0E-09	0.024
TRMT61B	29082931	SNP_A-8472586	29150981	765	5.0E-09	0.013
TRMT61B	29082931	rs62131971	29044280	736	5.1E-09	0.014
TRMT61B	29082931	rs7598876	28974466	739	5.1E-09	0.034
TRMT61B	29082931	rs7601720	29032478	759	5.3E-09	0.012
TRMT61B	29082931	chr2:29011337:D	29011337	757	5.3E-09	0.0093
TRMT61B	29082931	rs11690423	28951537	763	5.4E-09	0.041
TRMT61B	29082931	rs6728459	28999336	743	5.5E-09	0.0026
TRMT61B	29082931	rs72782294	28953390	763	6.0E-09	0.046
TRMT61B	29082931	rs7580078	28997001	748	6.2E-09	0.0031
TRMT61B	29082931	rs111534567	28949773	761	6.2E-09	0.042
TRMT61B	29082931	chr2:28952183:D	28952183	745	6.2E-09	0.046
TRMT61B	29082931	rs4558548	28985674	720	6.3E-09	0.00047
TRMT61B	29082931	rs7601934	29032673	761	6.3E-09	0.014
TRMT61B	29082931	rs13019104	29094928	737	7.3E-09	0.0096
TRMT61B	29082931	rs745645	28931571	762	7.4E-09	0.03
TRMT61B	29082931	rs4580350	28965156	753	7.4E-09	0.04
TRMT61B	29082931	rs4640348	28965479	753	7.4E-09	0.042
TRMT61B	29082931	rs2169751	28966758	753	7.4E-09	0.039
TRMT61B	29082931	rs11683296	28925404	763	7.5E-09	0.029
TRMT61B	29082931	rs56240884	28940154	764	7.7E-09	0.022
TRMT61B	29082931	rs13019397	29016019	729	8.1E-09	0.0052
TRMT61B	29082931	rs13009357	29041877	760	8.2E-09	0.012

TRMT61B	29082931	rs12714249	29055916	762	8.5E-09	0.0099
TRMT61B	29082931	rs6728045	29083075	758	8.6E-09	0.01
TRMT61B	29082931	chr2:29011335:D	29011335	702	8.7E-09	0.012
TRMT61B	29082931	rs4233727	28977244	749	8.7E-09	0.031
TRMT61B	29082931	rs2169748	28957753	763	9.0E-09	0.037
TRMT61B	29082931	rs13030835	29017474	763	9.1E-09	0.01
TRMT61B	29082931	rs13025081	29083850	765	9.6E-09	0.011
TRMT61B	29082931	rs6760186	29087274	765	9.6E-09	0.011
TRMT61B	29082931	rs13030945	29087799	765	9.6E-09	0.011
TRMT61B	29082931	rs10177006	29088256	765	9.6E-09	0.011
TRMT61B	29082931	rs34409160	28943865	761	9.7E-09	0.034
TRMT61B	29082931	rs13417543	29068457	764	9.8E-09	0.0098
TRMT61B	29082931	rs13382966	29068882	764	9.8E-09	0.0099
TRMT61B	29082931	rs7597048	29066756	757	1.0E-08	0.0099
TRMT61B	29082931	rs7562170	28943516	761	1.0E-08	0.044
TRMT61B	29082931	rs2045884	28964355	747	1.1E-08	0.036
TRMT61B	29082931	rs55835850	28949296	741	1.1E-08	0.045
TRMT61B	29082931	SNP_A-2079054	28932502	765	1.2E-08	0.029
TRMT61B	29082931	rs7607844	28971738	749	1.2E-08	0.025
TRMT61B	29082931	SNP_A-2202990	29032746	765	1.3E-08	0.013
TRMT61B	29082931	rs55877710	28929308	760	1.3E-08	0.026
TRMT61B	29082931	rs55883854	28929541	760	1.3E-08	0.026
TRMT61B	29082931	rs34627117	28930476	761	1.4E-08	0.026
TRMT61B	29082931	SNP_A-1867530	29032238	765	1.4E-08	0.013
TRMT61B	29082931	rs6725177	29007855	762	1.5E-08	0.0029
TRMT61B	29082931	rs10188412	29000287	762	1.6E-08	0.0029
TRMT61B	29082931	chr2:28943346:D	28943346	757	1.6E-08	0.039
TRMT61B	29082931	rs6710959	28958603	759	1.8E-08	0.039
TRMT61B	29082931	rs2293553	28932284	764	2.0E-08	0.035
TRMT61B	29082931	chr2:28996536:D	28996536	740	2.3E-08	0.0028
TRMT61B	29082931	rs55836936	28922833	755	2.5E-08	0.036
TRMT61B	29082931	SNP_A-2087248	29070805	765	2.6E-08	0.0099
TRMT61B	29082931	rs10179580	29088338	743	2.7E-08	0.0083
TRMT61B	29082931	rs11677127	28945646	755	3.2E-08	0.039
TRMT61B	29082931	rs11675572	28931376	754	4.1E-08	0.026
TRMT61B	29082931	rs4531886	28917964	761	5.4E-08	0.034
TRMT61B	29082931	rs12463507	29068519	726	7.1E-08	0.36
TRMT61B	29082931	rs72782280	28911349	756	2.7E-07	0.049
TRMT61B	29082931	rs61275598	28911091	735	6.4E-07	0.04
TRMT61B	29082931	rs12613887	29111099	735	1.3E-06	8.60E-06
TRMT61B	29082931	rs7371404	29111818	742	1.7E-06	6.10E-06
TRMT61B	29082931	rs12622793	29111106	735	2.1E-06	6.00E-06
TRMT61B	29082931	rs12622122	29111060	728	2.6E-06	4.40E-06
TRMT61B	29082931	SNP_A-1823752	29100168	765	4.6E-06	0.039

TRMT61B	29082931	rs34921009	29099285	765	5.1E-06	0.037
TRMT61B	29082931	rs60617439	29099528	765	5.1E-06	0.037
TRMT61B	29082931	rs12472404	29179452	761	9.0E-06	1.80E-06
TRMT61B	29082931	rs7580240	29114372	755	9.9E-06	9.90E-06
TRMT61B	29082931	rs4666151	29177344	765	1.0E-05	5.60E-06
TRMT61B	29082931	rs72786123	29177823	765	1.0E-05	5.40E-06
TRMT61B	29082931	***rs67073037**	29119585	757	1.5E-05	3.90E-06
TRMT61B	29082931	rs11680458	29170623	765	1.8E-05	4.70E-06
TRMT61B	29082931	rs1131880	29170676	765	1.8E-05	4.00E-06
TRMT61B	29082931	rs12465505	29171085	765	1.8E-05	4.70E-06
TRMT61B	29082931	rs12477538	29171288	765	1.8E-05	4.00E-06
TRMT61B	29082931	chr2:29171587:D	29171587	765	1.8E-05	4.70E-06
TRMT61B	29082931	rs4666144	29174105	765	1.8E-05	8.30E-06
TRMT61B	29082931	rs3885709	29168848	764	1.8E-05	5.00E-06
TRMT61B	29082931	rs6734079	29160421	760	1.9E-05	2.50E-06
TRMT61B	29082931	rs12465258	29162904	759	2.6E-05	9.70E-06
TRMT61B	29082931	rs67612857	29161707	763	2.7E-05	5.40E-06
TRMT61B	29082931	rs3924271	29167692	763	2.7E-05	4.30E-06
TRMT61B	29082931	rs4666142	29171544	765	3.0E-05	6.80E-06
TRMT61B	29082931	rs4666125	29112923	751	3.1E-05	8.80E-06
TRMT61B	29082931	rs4666129	29136136	749	3.1E-05	7.70E-06
TRMT61B	29082931	rs4407214	29118258	765	3.2E-05	7.40E-06
TRMT61B	29082931	rs66604446	29119930	765	3.2E-05	7.80E-06
TRMT61B	29082931	rs66768547	29120116	765	3.2E-05	7.80E-06
TRMT61B	29082931	SNP_A-2170642	29171381	765	3.3E-05	6.80E-06
TRMT61B	29082931	SNP_A-2150329	29129418	765	3.5E-05	4.30E-06
TRMT61B	29082931	rs79226661	28970001	737	3.6E-05	0.58
TRMT61B	29082931	rs4233728	28977274	711	3.9E-05	0.2
TRMT61B	29082931	rs4438440	29121611	764	4.1E-05	7.70E-06
TRMT61B	29082931	rs150477535	29013391	727	4.2E-05	0.48
TRMT61B	29082931	rs4371318	29156360	763	4.7E-05	9.30E-06
TRMT61B	29082931	rs4666141	29164022	763	4.7E-05	6.90E-06
TRMT61B	29082931	rs3924272	29167678	763	4.7E-05	6.20E-06
TRMT61B	29082931	rs11695230	29130140	764	4.7E-05	5.90E-06
TRMT61B	29082931	rs67873458	29119773	765	5.4E-05	1.10E-05
TRMT61B	29082931	rs6721781	29120030	765	5.4E-05	1.10E-05
TRMT61B	29082931	rs11677485	29146094	764	5.4E-05	6.60E-06
TRMT61B	29082931	rs12620306	29133600	757	5.5E-05	1.10E-05
TRMT61B	29082931	rs147684450	29008220	744	5.5E-05	0.5
TRMT61B	29082931	SNP_A-2006240	29120733	765	5.6E-05	1.30E-05
TRMT61B	29082931	rs4666128	29134513	763	6.1E-05	7.70E-06
TRMT61B	29082931	rs11677283	29151035	765	6.3E-05	5.70E-06

eQTL BC241

Gene	ePos	SNP	mPos	N	P-value	P1df_ERneg
TRMT61B	29082931	SNP_A-8472586	29150981	765	5.04E-09	0.013
TRMT61B	29082931	rs12613887	29111099	735	1.32E-06	8.60E-06
TRMT61B	29082931	rs7371404	29111818	742	1.69E-06	6.10E-06
TRMT61B	29082931	rs12622793	29111106	735	2.12E-06	6.00E-06
TRMT61B	29082931	rs12622122	29111060	728	2.64E-06	4.40E-06
TRMT61B	29082931	rs12472404	29179452	761	8.99E-06	1.80E-06
TRMT61B	29082931	rs7580240	29114372	755	9.91E-06	9.90E-06
TRMT61B	29082931	rs4666151	29177344	765	9.97E-06	5.60E-06
TRMT61B	29082931	rs72786123	29177823	765	9.97E-06	5.40E-06
TRMT61B	29082931	rs67073037	29119585	757	1.47E-05	3.90E-06
TRMT61B	29082931	rs11680458	29170623	765	1.75E-05	4.70E-06
TRMT61B	29082931	rs1131880	29170676	765	1.75E-05	4.00E-06
TRMT61B	29082931	rs12465505	29171085	765	1.75E-05	4.70E-06
TRMT61B	29082931	rs12477538	29171288	765	1.75E-05	4.00E-06
TRMT61B	29082931	chr2:29171587:D	29171587	765	1.75E-05	4.70E-06
TRMT61B	29082931	rs4666144	29174105	765	1.75E-05	8.30E-06
TRMT61B	29082931	rs3885709	29168848	764	1.84E-05	5.00E-06
TRMT61B	29082931	rs6734079	29160421	760	1.85E-05	2.50E-06
TRMT61B	29082931	rs12465258	29162904	759	2.60E-05	9.70E-06
TRMT61B	29082931	rs67612857	29161707	763	2.74E-05	5.40E-06
TRMT61B	29082931	rs3924271	29167692	763	2.74E-05	4.30E-06
TRMT61B	29082931	rs4666142	29171544	765	3.00E-05	6.80E-06
TRMT61B	29082931	rs4666125	29112923	751	3.11E-05	8.80E-06
TRMT61B	29082931	rs4666129	29136136	749	3.11E-05	7.70E-06
TRMT61B	29082931	rs4407214	29118258	765	3.17E-05	7.40E-06
TRMT61B	29082931	rs66604446	29119930	765	3.17E-05	7.80E-06
TRMT61B	29082931	rs66768547	29120116	765	3.17E-05	7.80E-06
TRMT61B	29082931	SNP_A-2170642	29171381	765	3.29E-05	6.80E-06
TRMT61B	29082931	SNP_A-2150329	29129418	765	3.54E-05	4.30E-06
TRMT61B	29082931	rs4438440	29121611	764	4.13E-05	7.70E-06
TRMT61B	29082931	rs4371318	29156360	763	4.69E-05	9.30E-06
TRMT61B	29082931	rs4666141	29164022	763	4.69E-05	6.90E-06
TRMT61B	29082931	rs3924272	29167678	763	4.69E-05	6.20E-06
TRMT61B	29082931	rs11695230	29130140	764	4.73E-05	5.90E-06
TRMT61B	29082931	rs67873458	29119773	765	5.38E-05	1.10E-05
TRMT61B	29082931	rs6721781	29120030	765	5.38E-05	1.10E-05
TRMT61B	29082931	rs11677485	29146094	764	5.44E-05	6.60E-06
TRMT61B	29082931	rs12620306	29133600	757	5.46E-05	1.10E-05
TRMT61B	29082931	rs4577254	29120733	765	5.61E-05	1.30E-05
TRMT61B	29082931	rs4666128	29134513	763	6.10E-05	7.70E-06
TRMT61B	29082931	rs11677283	29151035	765	6.29E-05	5.70E-06
TRMT61B	29082931	rs6743535	29135979	754	6.31E-05	4.00E-06
TRMT61B	29082931	rs12471649	29143425	764	6.54E-05	6.70E-06

TRMT61B	29082931	rs4666127	29127745	765	6.73E-05	7.50E-06
TRMT61B	29082931	rs4666134	29136653	765	6.73E-05	1.30E-05
TRMT61B	29082931	rs4666137	29137773	765	6.73E-05	6.10E-06
TRMT61B	29082931	rs4273183	29138436	765	6.73E-05	7.10E-06
TRMT61B	29082931	rs11689719	29122001	764	6.99E-05	1.10E-05
TRMT61B	29082931	rs12616009	29123191	763	7.04E-05	1.30E-05
TRMT61B	29082931	rs57693531	29129682	764	7.05E-05	4.30E-06
TRMT61B	29082931	rs11688844	29129923	764	7.05E-05	4.30E-06
TRMT61B	29082931	rs11684312	29130224	764	7.05E-05	4.30E-06
TRMT61B	29082931	rs66692018	29131987	764	7.05E-05	4.20E-06
TRMT61B	29082931	chr2:29134578:D	29134578	760	7.17E-05	1.20E-05
TRMT61B	29082931	rs4666135	29137567	759	7.60E-05	7.30E-06
TRMT61B	29082931	rs55717610	29174225	747	8.76E-05	0.045
TRMT61B	29082931	chr2:29161322:I	29161322	762	8.82E-05	0.036
TRMT61B	29082931	chr2:29151714:I	29151714	759	9.18E-05	6.10E-06
TRMT61B	29082931	rs4533438	29112143	753	9.80E-05	0.026

eProbe: expression array probe for candidate gene; ePos: Location of candidate gene; mPos: Location of SNP.
Genome build GRCh37.13; N: number of samples assessed; P-value (chi-square): significance of association between SNP and gene expression.

Supplementary Table 18. Conditional analysis of top eQTL and ER-negative risk SNPs in novel loci

Chr	SNP type	SNP	Conditional beta	Conditional SE	Conditional p-value
Chr 2p23	eQTL	rs6419696	$8.01x10^{-3}$	$2.40x10^{-2}$	0.74
	ER-negative risk	rs4577244	$-9.44x10^{-2}$	$2.85x10^{-2}$	$9.07x10^{-4}$
Chr13q22	eQTL	rs67241368	$-1.48x10^{-2}$	$1.94x10^{-2}$	0.44
	ER-negative risk	rs6562760	$5.14x10^{-2}$	$2.30x10^{-2}$	0.025
	ER-negative risk	rs12870942	$7.21x10^{-2}$	$2.07x10^{-2}$	$4.94x10^{-4}$
Chr 2q33	eQTL	rs2136600	$-5.58x10^{-2}$	$2.36x10^{-2}$	0.018
	ER-negative risk	rs115635831	0.36	$7.15x10^{-2}$	$4.60x10^{-7}$

P-value (chi-squate): significance of association between SNPs

Supplementary Table 19. 13q22 eQTL SNPs from BC765, BC241, and NB116 cohorts

**eQTL BC765**						
**Probe**	**Gene**	**ePos**	**SNP**	**mPos**	**N**	**P-value**
RNASeq	PIBF1	73473411	rs4885057	73596617	733	9.04E-06
RNASeq	PIBF1	73473411	rs9543180	73521987	730	3.75E-05
RNASeq	PIBF1	73473411	rs9543186	73539019	741	0.000157
RNASeq	PIBF1	73473411	rs75864382	73560777	759	0.000169
RNASeq	PIBF1	73473411	rs9543189	73565273	759	0.000169
RNASeq	PIBF1	73473411	rs76313597	73503177	760	0.000178
RNASeq	PIBF1	73473411	rs9543177	73503775	760	0.000178
RNASeq	PIBF1	73473411	rs9530118	73526502	760	0.000178
RNASeq	PIBF1	73473411	rs9543182	73526528	760	0.000178
RNASeq	PIBF1	73473411	rs9530121	73528001	760	0.000178
RNASeq	PIBF1	73473411	chr13:73551760:I	73551760	760	0.000178
RNASeq	PIBF1	73473411	rs75497275	73552634	760	0.000178
RNASeq	PIBF1	73473411	rs74930540	73553774	760	0.000178
RNASeq	PIBF1	73473411	rs9543194	73568704	758	0.000181
RNASeq	PIBF1	73473411	rs2151318	73527197	761	0.000188
RNASeq	PIBF1	73473411	rs8002896	73560141	719	0.000218
RNASeq	PIBF1	73473411	rs2325492	73556824	760	0.000244
RNASeq	PIBF1	73473411	rs4885054	73544874	763	0.000249
RNASeq	PIBF1	73473411	rs9318130	73550225	763	0.000249
RNASeq	PIBF1	73473411	rs1000973	73555632	763	0.000249
RNASeq	PIBF1	73473411	rs4885051	73500247	764	0.000252
RNASeq	PIBF1	73473411	rs9573066	73502928	764	0.000252
RNASeq	PIBF1	73473411	rs9573069	73519628	764	0.000252
RNASeq	PIBF1	73473411	rs9573071	73526335	764	0.000252
RNASeq	PIBF1	73473411	rs9573072	73533197	764	0.000252
RNASeq	PIBF1	73473411	rs6562733	73534034	763	0.000252
RNASeq	PIBF1	73473411	rs9573073	73539853	764	0.000252
RNASeq	PIBF1	73473411	rs9592873	73541331	764	0.000252
RNASeq	PIBF1	73473411	rs6562734	73545222	762	0.000255
RNASeq	PIBF1	73473411	rs7317863	73551492	762	0.000255
RNASeq	PIBF1	73473411	rs9573067	73503007	764	0.000261
RNASeq	PIBF1	73473411	rs7322181	73523754	764	0.000261
RNASeq	MZT1	73292216.5	rs9573170	73952698	752	0.000271
RNASeq	PIBF1	73473411	rs12872054	73521703	763	0.000281
RNASeq	PIBF1	73473411	rs1475688	73520224	756	0.000321
RNASeq	PIBF1	73473411	SNP_A-8469710	73503847	765	0.000379
RNASeq	PIBF1	73473411	rs1547286	73591805	753	0.000398
RNASeq	KLF5	73642409	rs74775679	74422000	745	0.000423
RNASeq	PIBF1	73473411	rs9564930	73500450	764	0.000426
RNASeq	PIBF1	73473411	rs1591145	73527395	764	0.000426
RNASeq	PIBF1	73473411	rs9573074	73541594	764	0.000426
RNASeq	KLF12	74484107.5	rs9543449	74340075	760	0.000448
RNASeq	PIBF1	73473411	chr13:73551318:D	73551318	696	0.000471
RNASeq	KLF5	73642409	rs73537919	74425396	760	0.000506
RNASeq	MZT1	73292216.5	rs9573172	73953498	758	0.000523
RNASeq	KLF5	73642409	rs75623818	74432865	708	0.000592
RNASeq	MZT1	73292216.5	rs9592908	73954363	765	0.00072
RNASeq	MZT1	73292216.5	rs9600118	73954675	765	0.00072
RNASeq	MZT1	73292216.5	rs9600119	73954865	765	0.00072
RNASeq	MZT1	73292216.5	rs9600120	73956435	765	0.00072
RNASeq	MZT1	73292216.5	rs9573173	73956666	765	0.00072
RNASeq	MZT1	73292216.5	rs9573174	73956787	765	0.00072
RNASeq	MZT1	73292216.5	rs9573176	73957464	765	0.00072
RNASeq	MZT1	73292216.5	rs9573177	73957589	765	0.00072
RNASeq	MZT1	73292216.5	rs9573178	73957620	765	0.00072
RNASeq	MZT1	73292216.5	rs9573179	73957719	765	0.00072
RNASeq	MZT1	73292216.5	rs9573180	73958504	765	0.00072
RNASeq	MZT1	73292216.5	rs9564972	73958814	765	0.00072
RNASeq	MZT1	73292216.5	rs9564973	73958923	765	0.00072
RNASeq	MZT1	73292216.5	rs9564974	73959005	765	0.00072
RNASeq	MZT1	73292216.5	rs9573182	73960140	765	0.00072

RNASeq	MZT1	73292216.5	rs9573183	73961563	765	0.00072
RNASeq	MZT1	73292216.5	rs9592911	73963492	765	0.00072
RNASeq	MZT1	73292216.5	rs17090134	73966659	765	0.00072
RNASeq	MZT1	73292216.5	rs17090136	73966756	765	0.00072
RNASeq	PIBF1	73473411	rs9564936	73587814	733	0.000736
RNASeq	PIBF1	73473411	rs9592877	73595330	756	0.000837
RNASeq	PIBF1	73473411	rs9600060	73595560	756	0.000837
RNASeq	PIBF1	73473411	rs9530135	73586731	746	0.000898
RNASeq	KLF12	74484107.5	SNP_A-1918937	74339344	765	0.000941
RNASeq	PIBF1	73473411	rs9543193	73568576	745	0.000949

eQTL BC241

Probe	Gene	ePos	SNP	mPos	N	P-value
A_23_P48416	DIS3	73342941.5	rs9543113	73341750	236	1.78E-05
A_23_P48416	DIS3	73342941.5	rs7332388	73349359	236	1.78E-05
A_24_P219024	DIS3	73342941.5	rs8001639	73262337	237	7.95E-05
A_24_P219024	DIS3	73342941.5	rs9543080	73268372	233	8.63E-05
A_24_P219024	DIS3	73342941.5	rs9573012	73260154	238	9.79E-05
A_24_P219024	DIS3	73342941.5	rs1073649	73264563	238	9.79E-05
A_24_P219024	DIS3	73342941.5	rs1073650	73264752	238	9.79E-05
A_24_P219024	DIS3	73342941.5	rs7328685	73265858	238	9.79E-05
A_24_P219024	DIS3	73342941.5	rs9573014	73266266	238	9.79E-05
A_24_P219024	DIS3	73342941.5	rs1440868	73267564	234	0.000108
A_24_P219024	DIS3	73342941.5	rs9543078	73267297	236	0.000116
A_24_P219024	DIS3	73342941.5	rs9573010	73255214	234	0.000119
A_24_P219024	DIS3	73342941.5	rs11148920	73280658	230	0.000161
A_23_P48416	DIS3	73342941.5	rs1318737	73341211	236	0.000207
A_23_P48416	DIS3	73342941.5	rs7332140	73349225	236	0.000207
A_24_P219024	DIS3	73342941.5	rs9318115	73285521	231	0.000231
A_24_P219024	DIS3	73342941.5	rs3850048	73258232	237	0.000244
A_23_P48416	DIS3	73342941.5	rs7333100	73343714	232	0.00028
A_23_P48416	DIS3	73342941.5	rs2043976	73367642	241	0.000293
A_23_P48416	DIS3	73342941.5	rs1372000	73369642	241	0.000293
A_23_P48416	DIS3	73342941.5	chr13:73377404:D	73377404	241	0.000293
A_23_P48416	DIS3	73342941.5	rs723944	73379684	241	0.000293
A_23_P48416	DIS3	73342941.5	rs7331370	73387699	241	0.000293
A_23_P48416	DIS3	73342941.5	rs1886659	73392190	241	0.000293
A_23_P48416	DIS3	73342941.5	rs7989157	73394217	241	0.000293
A_23_P48416	DIS3	73342941.5	rs7321471	73402193	241	0.000293
A_23_P48416	DIS3	73342941.5	rs74573585	73404149	241	0.000293
A_23_P48416	DIS3	73342941.5	rs11840575	73405652	241	0.000293
A_23_P48416	DIS3	73342941.5	rs997186	73413500	241	0.000293
A_24_P219024	DIS3	73342941.5	rs9573008	73253625	238	0.000297
A_24_P219024	DIS3	73342941.5	rs9573009	73253700	240	0.000313
A_24_P219024	DIS3	73342941.5	rs9564911	73253728	240	0.000313
A_24_P219024	DIS3	73342941.5	rs9564912	73254802	240	0.000313
A_24_P219024	DIS3	73342941.5	rs9564913	73255104	240	0.000313
A_24_P219024	DIS3	73342941.5	rs9573011	73255354	240	0.000313
A_24_P219024	DIS3	73342941.5	rs9543063	73255745	240	0.000313
A_24_P219024	DIS3	73342941.5	rs9543067	73256676	240	0.000313
A_24_P219024	DIS3	73342941.5	rs4119478	73254212	241	0.000338
A_23_P48416	DIS3	73342941.5	rs7333860	73405729	240	0.000368
A_23_P48416	DIS3	73342941.5	rs7988026	73377983	238	0.000382
A_24_P219024	DIS3	73342941.5	rs9543065	73255886	238	0.000475
A_23_P48416	DIS3	73342941.5	rs1892382	73426947	234	0.000589
A_23_P48416	DIS3	73342941.5	rs7998287	73444518	233	0.000632
A_23_P48416	DIS3	73342941.5	rs17282491	73441845	236	0.000652
A_23_P48416	DIS3	73342941.5	rs61181891	73368088	238	0.000707
A_23_P48416	DIS3	73342941.5	rs17196441	73399452	238	0.000707
A_23_P48416	DIS3	73342941.5	rs11148923	73417074	238	0.000707
A_23_P48416	DIS3	73342941.5	rs12583408	73417437	238	0.000707
A_23_P48416	DIS3	73342941.5	rs117641126	73419315	238	0.000707
A_23_P48416	DIS3	73342941.5	rs17209721	73423663	238	0.000707
A_23_P48416	DIS3	73342941.5	rs61967068	73431009	238	0.000707

A_23_P48416	DIS3	73342941.5	rs61967071	73432351	238	0.000707
A_23_P48416	DIS3	73342941.5	rs10492655	73435207	238	0.000707
A_23_P48416	DIS3	73342941.5	chr13:73436758:D	73436758	238	0.000707
A_23_P48416	DIS3	73342941.5	rs12429077	73442034	238	0.000707
A_23_P48416	DIS3	73342941.5	rs11148924	73446411	238	0.000707
A_23_P48416	DIS3	73342941.5	rs12583255	73448228	238	0.000707
A_23_P48416	DIS3	73342941.5	rs12429272	73464429	238	0.000707
A_23_P48416	DIS3	73342941.5	rs11839523	73418726	237	0.000841
A_24_P219024	DIS3	73342941.5	rs9564914	73269449	229	0.000857

eQTL NB116

Probe	Gene	ePos	SNP	mPos	N	P-value
A_23_P354805	KLF12	74484107	rs112349378	74034883	111	0.00104
A_23_P354805	KLF12	74484107	rs73226438	74023847	112	0.00133
A_23_P48416	DIS3	73342941.5	rs9573103	73658273	116	0.00205
A_23_P48416	DIS3	73342941.5	rs9564942	73658544	116	0.00205
A_23_P354805	KLF12	74484107	rs17090217	74039235	115	0.00222
A_23_P354805	KLF12	74484107	rs10507815	74039881	115	0.00222
A_23_P354805	KLF12	74484107	rs10507816	74039991	115	0.00222
A_24_P219024	DIS3	73342941.5	rs9543285	73812537	114	0.00229
A_24_P219024	DIS3	73342941.5	rs12870942	73806982	116	0.00234
A_24_P219024	DIS3	73342941.5	rs17181761	73811471	116	0.00234
A_24_P219024	DIS3	73342941.5	rs9573140	73813803	116	0.00234
A_24_P219024	DIS3	73342941.5	rs9543287	73814441	116	0.00234
A_24_P219024	DIS3	73342941.5	rs9530173	73814697	116	0.00234
A_24_P219024	DIS3	73342941.5	rs9530172	73814192	114	0.00353
A_23_P354805	KLF12	74484107	rs9564995	74038967	116	0.00353
A_23_P354805	KLF12	74484107	rs9564996	74039264	116	0.00353
A_24_P395317	DIS3	73342941.5	rs9573194	74004376	114	0.00383
A_24_P395317	DIS3	73342941.5	rs9564987	74006439	114	0.00383
A_24_P395317	DIS3	73342941.5	rs2093943	74007186	114	0.00383
A_24_P395317	DIS3	73342941.5	rs9573198	74008908	114	0.00383
A_24_P395317	DIS3	73342941.5	rs7332303	73850989	116	0.00591
A_24_P395317	DIS3	73342941.5	rs1576081	73852564	116	0.00591
A_24_P395317	DIS3	73342941.5	rs7987880	73853301	116	0.00591
A_24_P395317	DIS3	73342941.5	rs9543306	73853762	112	0.00669
A_23_P354805	KLF12	74484107	rs12100281	74038925	113	0.00691
A_23_P53891	KLF5	73642408.5	rs9543374	74044117	115	0.00735
A_23_P53891	KLF5	73642408.5	rs1547147	74047149	114	0.0075
A_23_P354805	KLF12	74484107	rs67393769	74211277	115	0.00751
A_23_P53891	KLF5	73642408.5	rs7327558	74007487	114	0.00767
A_23_P354805	KLF12	74484107	rs9530216	74201598	116	0.00799
A_24_P210406	KLF5	73642408.5	rs7333339	73747284	116	0.00802
A_23_P53891	KLF5	73642408.5	rs9573221	74047191	115	0.00874
A_23_P53891	KLF5	73642408.5	rs9543375	74047412	115	0.00874
A_23_P53891	KLF5	73642408.5	rs9543376	74047729	115	0.00874
A_23_P53891	KLF5	73642408.5	rs719825	74041152	116	0.00885
A_23_P53891	KLF5	73642408.5	rs719826	74041419	116	0.00885
A_23_P354805	KLF12	74484107	chr13:73780388:D	73780388	112	0.00983
A_23_P354805	KLF12	74484107	rs11840000	73780764	112	0.00983
A_23_P354805	KLF12	74484107	rs9543270	73782110	112	0.00983
A_23_P48416	DIS3	73342941.5	rs3003858	73669891	113	0.00986

eProbe: expression array probe for candidate gene; ePos: Location of candidate gene; mPos: Location of SNP. Genome build GRCh37.13; N: number of samples assessed; P-value: Chi-square significance of association between SNP and gene expression.

Supplementary Table 20. 2q33 eQTL analysis

eQTL 2q33						
**BC765**						
**Probe**	**Gene**	**ePos**	**SNP**	**mPos**	**N**	P-value
RNASeq	PPIL3	201744764	rs2136600	201742345	762	1.61E-83
RNASeq	PPIL3	201744764	rs7559150	201754063	753	3.18E-83
RNASeq	PPIL3	201744764	rs11894842	201773545	763	9.75E-83
RNASeq	PPIL3	201744764	rs13412430	201774355	763	9.75E-83
RNASeq	PPIL3	201744764	rs7588993	201763866	761	9.98E-83
RNASeq	PPIL3	201744764	rs6435066	201766023	761	9.98E-83
RNASeq	PPIL3	201744764	rs7917	201768238	761	9.98E-83
RNASeq	PPIL3	201744764	rs10172647	201778950	760	1.53E-82
RNASeq	PPIL3	201744764	chr2:201787159:I	201787159	762	2.69E-82
RNASeq	PPIL3	201744764	rs13412214	201774182	761	4.00E-82
RNASeq	PPIL3	201744764	rs6716122	201786474	746	5.75E-81
RNASeq	PPIL3	201744764	rs3888610	201732117	750	9.95E-80
RNASeq	PPIL3	201744764	rs7606251	201736734	750	5.22E-79
RNASeq	PPIL3	201744764	rs13421776	201760902	765	2.20E-72
RNASeq	PPIL3	201744764	chr2:201762893:D	201762893	764	3.94E-72
RNASeq	PPIL3	201744764	rs4381763	201776988	764	3.94E-72
RNASeq	PPIL3	201744764	rs73059141	201788793	765	7.90E-72
RNASeq	PPIL3	201744764	SNP_A-8710249	201797676	765	7.90E-72
RNASeq	PPIL3	201744764	rs6754769	201749852	754	1.27E-70
RNASeq	PPIL3	201744764	chr2:201746389:I	201746389	764	1.89E-69
RNASeq	PPIL3	201744764	rs73059116	201730687	749	2.20E-67
RNASeq	PPIL3	201744764	rs13406545	201792123	717	9.69E-63
RNASeq	PPIL3	201744764	SNP_A-2246809	201760937	765	1.08E-62
RNASeq	PPIL3	201744764	SNP_A-2168433	201762147	765	2.10E-62
RNASeq	PPIL3	201744764	chr2:201793024:D	201793024	759	2.91E-62
RNASeq	PPIL3	201744764	rs2307358	201785657	765	3.37E-62
RNASeq	PPIL3	201744764	rs113388793	201771341	763	3.92E-62
RNASeq	PPIL3	201744764	rs6754564	201779932	763	4.20E-62
RNASeq	PPIL3	201744764	rs4622700	201793113	760	8.95E-62
RNASeq	PPIL3	201744764	rs9288314	201794173	760	8.95E-62
RNASeq	PPIL3	201744764	SNP_A-1966500	201785837	765	7.10E-61
RNASeq	PPIL3	201744764	rs4035022	201744352	764	3.89E-60
RNASeq	PPIL3	201744764	SNP_A-4275189	201748415	765	9.83E-60
RNASeq	PPIL3	201744764	rs3851973	201732878	749	6.99E-58
RNASeq	PPIL3	201744764	rs73059115	201730590	752	7.66E-58
RNASeq	PPIL3	201744764	rs11892119	201736112	752	1.57E-57
RNASeq	PPIL3	201744764	rs7562391	201736166	752	1.57E-57
RNASeq	PPIL3	201744764	rs111976464	201735042	750	4.44E-57
RNASeq	PPIL3	201744764	rs138539278	201734921	741	2.50E-53
RNASeq	PPIL3	201744764	chr2:201735706:I	201735706	739	3.28E-51
RNASeq	PPIL3	201744764	chr2:201731757:D	201731757	725	8.74E-51
RNASeq	PPIL3	201744764	rs13404596	201771801	715	1.26E-50
RNASeq	PPIL3	201744764	rs13416500	201771798	702	1.43E-50
RNASeq	PPIL3	201744764	rs111315781	201738724	733	2.17E-49
RNASeq	PPIL3	201744764	rs57210488	201706347	719	6.87E-46
RNASeq	PPIL3	201744764	rs77356501	201650853	743	5.84E-33
RNASeq	PPIL3	201744764	SNP_A-2068188	201632512	765	2.31E-28
RNASeq	PPIL3	201744764	SNP_A-1954909	201634161	765	3.17E-27
RNASeq	PPIL3	201744764	rs75609538	201712263	724	6.70E-19
RNASeq	PPIL3	201744764	rs11888596	201628314	761	1.19E-15
RNASeq	PPIL3	201744764	SNP_A-8576952	201616390	765	1.10E-14
RNASeq	PPIL3	201744764	SNP_A-1959884	201739327	765	2.10E-14
RNASeq	PPIL3	201744764	SNP_A-2074586	201635535	765	1.00E-13
RNASeq	PPIL3	201744764	rs11695319	201627921	757	1.17E-13
RNASeq	PPIL3	201744764	rs2881836	201643582	758	1.76E-13
RNASeq	PPIL3	201744764	SNP_A-2110387	201632988	765	2.19E-13
RNASeq	PPIL3	201744764	rs115110433	201672670	758	4.70E-13
RNASeq	PPIL3	201744764	rs148854467	201702007	721	6.32E-13
RNASeq	PPIL3	201744764	rs78244989	201675851	758	7.43E-13

RNASeq	PPIL3	201744764	rs150506039	201684478	758	7.43E-13
RNASeq	PPIL3	201744764	rs11893944	201645836	737	1.16E-12
RNASeq	PPIL3	201744764	rs77404113	201640489	761	3.03E-11
RNASeq	PPIL3	201744764	rs76368892	201764069	764	3.79E-11
RNASeq	PPIL3	201744764	SNP_A-8576950	201616145	765	4.41E-11
RNASeq	PPIL3	201744764	SNP_A-2195400	201613891	765	7.32E-11
RNASeq	PPIL3	201744764	rs10202088	201638151	757	8.65E-11
RNASeq	PPIL3	201744764	rs2348123	201615806	763	3.39E-10
RNASeq	PPIL3	201744764	rs13392387	201611761	746	3.53E-10
RNASeq	PPIL3	201744764	chr2:201715261:I	201715261	756	5.46E-10
RNASeq	PPIL3	201744764	rs139299713	201781569	756	1.15E-09
RNASeq	PPIL3	201744764	rs1001250	201624818	751	3.10E-09
RNASeq	PPIL3	201744764	SNP_A-8546074	201633894	765	5.95E-09
RNASeq	PPIL3	201744764	rs12622452	201638647	760	6.37E-09
RNASeq	PPIL3	201744764	rs11693950	201627550	754	6.64E-09
RNASeq	PPIL3	201744764	rs6720418	201641373	762	7.23E-09
RNASeq	PPIL3	201744764	rs11683632	201640575	762	7.52E-09
RNASeq	PPIL3	201744764	rs12465952	201724360	725	8.36E-09
RNASeq	PPIL3	201744764	rs11903236	201724114	747	1.33E-08
RNASeq	PPIL3	201744764	rs76392095	201765780	750	2.57E-08
RNASeq	PPIL3	201744764	rs4616462	201695859	757	4.76E-08
RNASeq	CFLAR	202009144	rs10172647	201778950	760	5.74E-08
RNASeq	PPIL3	201744764	rs112313977	201720172	745	6.25E-08
RNASeq	PPIL3	201744764	rs17467707	201747681	762	6.37E-08
RNASeq	PPIL3	201744764	rs145878905	201749975	762	6.37E-08
RNASeq	PPIL3	201744764	SNP_A-8576954	201617950	765	6.45E-08
RNASeq	PPIL3	201744764	rs2348117	201598521	744	7.12E-08
RNASeq	PPIL3	201744764	chr2:201746550:D	201746550	763	8.69E-08
RNASeq	PPIL3	201744764	rs72929010	201747351	763	8.69E-08
RNASeq	PPIL3	201744764	rs17467616	201690994	765	9.02E-08
RNASeq	PPIL3	201744764	SNP_A-8600928	201692449	765	9.02E-08
RNASeq	CFLAR	202009144	chr2:201787159:I	201787159	762	9.37E-08
RNASeq	PPIL3	201744764	rs72929005	201736668	764	9.88E-08
RNASeq	PPIL3	201744764	rs72929009	201745160	764	9.88E-08
RNASeq	PPIL3	201744764	chr2:201675531:I	201675531	749	1.10E-07
RNASeq	CFLAR	202009144	rs7559150	201754063	753	1.12E-07
RNASeq	PPIL3	201744764	SNP_A-2284507	201745667	765	1.25E-07
RNASeq	CFLAR	202009144	rs6716122	201786474	746	1.25E-07
RNASeq	CFLAR	202009144	rs11894842	201773545	763	1.41E-07
RNASeq	CFLAR	202009144	rs13412430	201774355	763	1.41E-07
RNASeq	PPIL3	201744764	chr2:201732298:D	201732298	764	1.53E-07
RNASeq	PPIL3	201744764	rs188686860	201733341	758	1.77E-07
RNASeq	CFLAR	202009144	rs13412214	201774182	761	1.78E-07
RNASeq	CFLAR	202009144	rs6754769	201749852	754	2.08E-07
RNASeq	CFLAR	202009144	rs7588993	201763866	761	2.11E-07
RNASeq	CFLAR	202009144	rs6435066	201766023	761	2.11E-07
RNASeq	CFLAR	202009144	rs7917	201768238	761	2.11E-07
RNASeq	PPIL3	201744764	rs78258606	201729335	762	2.71E-07
RNASeq	PPIL3	201744764	chr2:201731754:D	201731754	762	2.71E-07
RNASeq	PPIL3	201744764	rs149780988	201732571	762	2.71E-07
RNASeq	PPIL3	201744764	rs145445307	201733955	762	2.71E-07
RNASeq	PPIL3	201744764	rs17467658	201726390	761	2.81E-07
RNASeq	PPIL3	201744764	rs4142282	201580817	757	5.04E-07
RNASeq	AOX2P	201609696	rs3731720	201692219	762	5.74E-07
RNASeq	PPIL3	201744764	rs114687445	201773257	765	5.85E-07
RNASeq	PPIL3	201744764	rs192952120	201778911	765	5.85E-07
RNASeq	PPIL3	201744764	rs190521476	201779820	765	5.85E-07
RNASeq	PPIL3	201744764	rs17467748	201782763	765	5.85E-07
RNASeq	PPIL3	201744764	chr2:201786050:I	201786050	765	5.85E-07
RNASeq	PPIL3	201744764	rs16835746	201787453	765	5.85E-07
RNASeq	PPIL3	201744764	rs76637130	201788479	765	5.85E-07
RNASeq	PPIL3	201744764	rs16835779	201789600	765	5.85E-07
RNASeq	PPIL3	201744764	rs148850748	201789638	765	5.85E-07
RNASeq	PPIL3	201744764	rs183216043	201793388	765	5.85E-07

RNASeq	PPIL3	201744764	rs17383256	201794885	765	5.85E-07
RNASeq	PPIL3	201744764	rs17383151	201739884	764	6.08E-07
RNASeq	PPIL3	201744764	rs138860166	201740114	764	6.08E-07
RNASeq	PPIL3	201744764	rs115635831	201743594	764	6.08E-07
RNASeq	PPIL3	201744764	rs139580051	201749127	764	6.08E-07
RNASeq	PPIL3	201744764	rs16835425	201750683	764	6.08E-07
RNASeq	PPIL3	201744764	rs16835784	201790439	762	6.69E-07
RNASeq	PPIL3	201744764	rs9679113	201584617	761	7.24E-07
RNASeq	PPIL3	201744764	rs148324804	201752652	762	7.47E-07
RNASeq	PPIL3	201744764	rs16835942	201799870	765	7.91E-07
RNASeq	PPIL3	201744764	rs72927023	201644768	745	8.66E-07
RNASeq	PPIL3	201744764	rs72927026	201644772	745	8.66E-07
RNASeq	PPIL3	201744764	chr2:201776336:D	201776336	761	8.68E-07
RNASeq	PPIL3	201744764	rs144128797	201783334	761	8.68E-07
RNASeq	PPIL3	201744764	rs9679668	201584382	762	8.73E-07
RNASeq	PPIL3	201744764	rs55931932	201761136	762	8.85E-07
RNASeq	PPIL3	201744764	rs2348114	201582193	756	8.89E-07
RNASeq	PPIL3	201744764	rs10200697	201595517	762	8.91E-07
RNASeq	PPIL3	201744764	rs145079048	201752651	758	9.02E-07
RNASeq	CFLAR	202009144	rs2136600	201742345	762	9.44E-07
RNASeq	CFLAR	202009144	rs7606251	201736734	750	9.63E-07
RNASeq	PPIL3	201744764	rs17533364	201764967	764	1.01E-06
RNASeq	PPIL3	201744764	chr2:201765921:I	201765921	764	1.01E-06
RNASeq	PPIL3	201744764	rs10497855	201766236	764	1.01E-06
RNASeq	PPIL3	201744764	rs116798201	201771019	764	1.01E-06
RNASeq	PPIL3	201744764	SNP_A-2115883	201592651	765	1.27E-06
RNASeq	CFLAR	202009144	rs3888610	201732117	750	1.76E-06
RNASeq	PPIL3	201744764	rs72927008	201619599	740	1.90E-06
RNASeq	PPIL3	201744764	rs79579604	201613658	760	1.94E-06
RNASeq	PPIL3	201744764	rs13028496	201645349	736	2.21E-06
RNASeq	PPIL3	201744764	rs74943274	201717014	761	2.37E-06
RNASeq	PPIL3	201744764	rs78529534	201717290	761	2.37E-06
RNASeq	PPIL3	201744764	rs79751905	201723140	761	2.37E-06
RNASeq	PPIL3	201744764	rs150365829	201723695	761	2.37E-06
RNASeq	PPIL3	201744764	rs13415568	201723902	761	2.37E-06
RNASeq	PPIL3	201744764	rs41271455	201724391	761	2.37E-06
RNASeq	PPIL3	201744764	rs111321288	201697204	764	2.71E-06
RNASeq	PPIL3	201744764	chr2:201750652:D	201750652	764	3.41E-06

eQTL 2q33 BC765 risk SNPs

Probe	Gene	ePos	SNP	mPos	N	P-value
RNASeq	PPIL3	201744764	rs188686860	201733341	760	1.77E-07
RNASeq	PPIL3	201744764	rs78258606	201729335	764	2.71E-07
RNASeq	PPIL3	201744764	rs145445307	201733955	764	2.71E-07
RNASeq	PPIL3	201744764	rs149780988	201732571	764	2.71E-07
RNASeq	PPIL3	201744764	chr2:201731754:D	201731754	764	2.71E-07
RNASeq	PPIL3	201744764	rs17467658	201726390	763	2.81E-07
RNASeq	PPIL3	201744764	chr2:201920924:I	201920924	763	3.05E-07
RNASeq	PPIL3	201744764	rs147538031	202112571	763	3.45E-07
RNASeq	PPIL3	201744764	rs116782073	201994992	767	5.85E-07
RNASeq	PPIL3	201744764	chr2:201786050:I	201786050	767	5.85E-07
RNASeq	PPIL3	201744764	chr2:201805255:I	201805255	767	5.85E-07
RNASeq	PPIL3	201744764	rs148850748	201789638	767	5.85E-07
RNASeq	PPIL3	201744764	rs16835746	201787453	767	5.85E-07
RNASeq	PPIL3	201744764	rs16835997	201800686	767	5.85E-07
RNASeq	PPIL3	201744764	rs16836061	201804562	767	5.85E-07
RNASeq	PPIL3	201744764	rs17383256	201794885	767	5.85E-07
RNASeq	PPIL3	201744764	rs17467748	201782763	767	5.85E-07
RNASeq	PPIL3	201744764	rs183216043	201793388	767	5.85E-07
RNASeq	PPIL3	201744764	rs192952120	201778911	767	5.85E-07
RNASeq	PPIL3	201744764	rs149297763	202019882	767	5.85E-07
RNASeq	PPIL3	201744764	rs189192934	202027036	767	5.85E-07
RNASeq	PPIL3	201744764	rs143709332	202006755	767	5.85E-07
RNASeq	PPIL3	201744764	rs189252016	201892272	767	5.85E-07

RNASeq	PPIL3	201744764	rs149336947	202003665	767	5.85E-07
RNASeq	PPIL3	201744764	rs116724456	202002617	767	5.85E-07
RNASeq	PPIL3	201744764	rs137937873	202034497	767	5.85E-07
RNASeq	PPIL3	201744764	rs148420167	201847573	767	5.85E-07
RNASeq	PPIL3	201744764	rs190521476	201779820	767	5.85E-07
RNASeq	PPIL3	201744764	rs112281084	201821221	767	5.85E-07
RNASeq	PPIL3	201744764	rs114687445	201773257	767	5.85E-07
RNASeq	PPIL3	201744764	rs75208666	201980843	767	5.85E-07
RNASeq	PPIL3	201744764	chr2:201840567:D	201840567	767	5.85E-07
RNASeq	PPIL3	201744764	chr2:201902058:D	201902058	767	5.85E-07
RNASeq	PPIL3	201744764	chr2:201931200:D	201931200	767	5.85E-07
RNASeq	PPIL3	201744764	rs114008573	201890604	767	5.85E-07
RNASeq	PPIL3	201744764	rs114247218	201976712	767	5.85E-07
RNASeq	PPIL3	201744764	rs114326395	201897787	767	5.85E-07
RNASeq	PPIL3	201744764	rs114567273	201870021	767	5.85E-07
RNASeq	PPIL3	201744764	rs114699270	201908835	767	5.85E-07
RNASeq	PPIL3	201744764	rs114962751	201935871	767	5.85E-07
RNASeq	PPIL3	201744764	rs115331342	201852394	767	5.85E-07
RNASeq	PPIL3	201744764	rs115928143	201916833	767	5.85E-07
RNASeq	PPIL3	201744764	rs116089517	201910772	767	5.85E-07
RNASeq	PPIL3	201744764	rs116186593	201911475	767	5.85E-07
RNASeq	PPIL3	201744764	rs116276983	201914847	767	5.85E-07
RNASeq	PPIL3	201744764	rs116309768	201911085	767	5.85E-07
RNASeq	PPIL3	201744764	rs116416403	201914166	767	5.85E-07
RNASeq	PPIL3	201744764	rs116500747	201919002	767	5.85E-07
RNASeq	PPIL3	201744764	rs116539488	201867467	767	5.85E-07
RNASeq	PPIL3	201744764	rs116700774	201865895	767	5.85E-07
RNASeq	PPIL3	201744764	rs116739540	201939958	767	5.85E-07
RNASeq	PPIL3	201744764	rs138504123	201895262	767	5.85E-07
RNASeq	PPIL3	201744764	rs139722324	201974896	767	5.85E-07
RNASeq	PPIL3	201744764	rs141852703	201949194	767	5.85E-07
RNASeq	PPIL3	201744764	rs142340339	201928037	767	5.85E-07
RNASeq	PPIL3	201744764	rs142608159	201842450	767	5.85E-07
RNASeq	PPIL3	201744764	rs144999911	201850107	767	5.85E-07
RNASeq	PPIL3	201744764	rs145216595	201927927	767	5.85E-07
RNASeq	PPIL3	201744764	rs145748840	201922345	767	5.85E-07
RNASeq	PPIL3	201744764	rs146272565	201899731	767	5.85E-07
RNASeq	PPIL3	201744764	rs146820497	201863860	767	5.85E-07
RNASeq	PPIL3	201744764	rs147647393	201871575	767	5.85E-07
RNASeq	PPIL3	201744764	rs148220417	201931008	767	5.85E-07
RNASeq	PPIL3	201744764	rs149765952	201892855	767	5.85E-07
RNASeq	PPIL3	201744764	rs150173964	201919563	767	5.85E-07
RNASeq	PPIL3	201744764	rs151067359	201876969	767	5.85E-07
RNASeq	PPIL3	201744764	rs17383382	201854025	767	5.85E-07
RNASeq	PPIL3	201744764	rs17467902	201846083	767	5.85E-07
RNASeq	PPIL3	201744764	rs17467916	201858087	767	5.85E-07
RNASeq	PPIL3	201744764	rs181144634	201927809	767	5.85E-07
RNASeq	PPIL3	201744764	rs184264743	201903323	767	5.85E-07
RNASeq	PPIL3	201744764	rs186994307	201903324	767	5.85E-07
RNASeq	PPIL3	201744764	rs75140790	201919375	767	5.85E-07
RNASeq	PPIL3	201744764	rs75853971	201858344	767	5.85E-07
RNASeq	PPIL3	201744764	rs80211752	201854462	767	5.85E-07
RNASeq	PPIL3	201744764	chr2:201966510:D	201966510	767	5.85E-07
RNASeq	PPIL3	201744764	chr2:201973150:D	201973150	767	5.85E-07
RNASeq	PPIL3	201744764	rs114176379	201969453	767	5.85E-07
RNASeq	PPIL3	201744764	rs114984720	201995860	767	5.85E-07
RNASeq	PPIL3	201744764	rs116509920	201958437	767	5.85E-07
RNASeq	PPIL3	201744764	rs138763733	201961225	767	5.85E-07
RNASeq	PPIL3	201744764	rs139445865	201962452	767	5.85E-07
RNASeq	PPIL3	201744764	rs148040754	201961493	767	5.85E-07
RNASeq	PPIL3	201744764	rs149458356	201973547	767	5.85E-07
RNASeq	PPIL3	201744764	rs17383533	201964642	767	5.85E-07
RNASeq	PPIL3	201744764	rs112762622	202032688	767	5.85E-07
RNASeq	PPIL3	201744764	rs138875581	201971529	767	5.85E-07

RNASeq	PPIL3	201744764	rs149206908	201947732	767	5.85E-07
RNASeq	PPIL3	201744764	chr2:201939858:D	201939858	767	5.85E-07
RNASeq	PPIL3	201744764	chr2:201951530:I	201951530	767	5.85E-07
RNASeq	PPIL3	201744764	rs111978201	201950540	767	5.85E-07
RNASeq	PPIL3	201744764	rs114710676	201937599	767	5.85E-07
RNASeq	PPIL3	201744764	rs116198267	201935861	767	5.85E-07
RNASeq	PPIL3	201744764	rs183774255	201938503	767	5.85E-07
RNASeq	PPIL3	201744764	rs75196967	201971887	767	5.85E-07
RNASeq	PPIL3	201744764	rs112065557	201946829	767	5.85E-07
RNASeq	PPIL3	201744764	rs113072211	201938297	767	5.85E-07
RNASeq	PPIL3	201744764	rs115300559	201942229	767	5.85E-07
RNASeq	PPIL3	201744764	rs139270392	201942774	767	5.85E-07
RNASeq	PPIL3	201744764	rs76655038	201943154	767	5.85E-07
RNASeq	PPIL3	201744764	rs115144903	201958283	767	5.85E-07
RNASeq	PPIL3	201744764	rs144069676	201893802	767	5.85E-07
RNASeq	PPIL3	201744764	rs79959919	201995639	767	5.85E-07
RNASeq	PPIL3	201744764	rs74482315	201978027	767	5.85E-07
RNASeq	PPIL3	201744764	rs74596795	201923635	767	5.85E-07
RNASeq	PPIL3	201744764	rs116331034	201945722	767	5.85E-07
RNASeq	PPIL3	201744764	rs11887751	201943431	767	5.85E-07
RNASeq	PPIL3	201744764	rs11888623	201946957	767	5.85E-07
RNASeq	PPIL3	201744764	rs11896935	201941977	767	5.85E-07
RNASeq	PPIL3	201744764	rs11897109	201942367	767	5.85E-07
RNASeq	PPIL3	201744764	rs142246292	201938442	767	5.85E-07
RNASeq	PPIL3	201744764	rs76260028	201954611	767	5.85E-07
RNASeq	PPIL3	201744764	chr2:201880105:I	201880105	767	5.85E-07
RNASeq	PPIL3	201744764	rs116230086	201958962	767	5.85E-07
RNASeq	PPIL3	201744764	rs11888847	201947403	767	5.85E-07
RNASeq	PPIL3	201744764	rs150638321	201892188	767	5.85E-07
RNASeq	PPIL3	201744764	rs60630751	201913284	767	5.85E-07
RNASeq	PPIL3	201744764	chr2:201865559:D	201865559	767	5.85E-07
RNASeq	PPIL3	201744764	chr2:201875490:I	201875490	767	5.85E-07
RNASeq	PPIL3	201744764	chr2:201898885:I	201898885	767	5.85E-07
RNASeq	PPIL3	201744764	chr2:201898888:I	201898888	767	5.85E-07
RNASeq	PPIL3	201744764	rs111564182	201901974	767	5.85E-07
RNASeq	PPIL3	201744764	rs115167130	201921441	767	5.85E-07
RNASeq	PPIL3	201744764	rs116301213	201920278	767	5.85E-07
RNASeq	PPIL3	201744764	rs116478550	201923157	767	5.85E-07
RNASeq	PPIL3	201744764	rs116699946	201924488	767	5.85E-07
RNASeq	PPIL3	201744764	rs11892279	201879455	767	5.85E-07
RNASeq	PPIL3	201744764	rs11900960	201869491	767	5.85E-07
RNASeq	PPIL3	201744764	rs16836551	201876052	767	5.85E-07
RNASeq	PPIL3	201744764	rs58669897	201924449	767	5.85E-07
RNASeq	PPIL3	201744764	rs58911199	201924358	767	5.85E-07
RNASeq	PPIL3	201744764	rs74325135	201929766	767	5.85E-07
RNASeq	PPIL3	201744764	rs75246650	201898427	767	5.85E-07
RNASeq	PPIL3	201744764	rs76377168	201924933	767	5.85E-07
RNASeq	PPIL3	201744764	rs78519396	201931610	767	5.85E-07
RNASeq	PPIL3	201744764	rs79395719	201872180	767	5.85E-07
RNASeq	PPIL3	201744764	rs16836506	201862428	767	5.85E-07
RNASeq	PPIL3	201744764	rs76520541	201870282	767	5.85E-07
RNASeq	PPIL3	201744764	rs11893239	201855198	767	5.85E-07
RNASeq	PPIL3	201744764	rs56911059	201857829	767	5.85E-07
RNASeq	PPIL3	201744764	rs11895568	201847877	767	5.85E-07
RNASeq	PPIL3	201744764	rs11899179	201837772	767	5.85E-07
RNASeq	PPIL3	201744764	rs116313342	201903636	767	5.85E-07
RNASeq	PPIL3	201744764	rs76637130	201788479	767	5.85E-07
RNASeq	PPIL3	201744764	rs11895356	201800167	767	5.85E-07
RNASeq	PPIL3	201744764	rs16835779	201789600	767	5.85E-07
RNASeq	PPIL3	201744764	rs75490883	201816239	767	5.85E-07
RNASeq	PPIL3	201744764	rs16836158	201813224	767	5.85E-07
RNASeq	PPIL3	201744764	rs115635831	201743594	766	6.08E-07
RNASeq	PPIL3	201744764	rs17383151	201739884	766	6.08E-07
RNASeq	PPIL3	201744764	rs139580051	201749127	766	6.08E-07

RNASeq	PPIL3	201744764	rs16835425	201750683	766	6.08E-07
RNASeq	PPIL3	201744764	rs144629284	201943996	764	6.54E-07
RNASeq	PPIL3	201744764	rs141180406	201931057	761	6.90E-07
RNASeq	PPIL3	201744764	rs16836607	201933121	767	2.17E-06
RNASeq	PPIL3	201744764	rs17383298	201819228	766	2.34E-06
RNASeq	PPIL3	201744764	rs13415568	201723902	763	2.37E-06
RNASeq	PPIL3	201744764	rs41271455	201724391	763	2.37E-06
RNASeq	PPIL3	201744764	rs78529534	201717290	763	2.37E-06
RNASeq	PPIL3	201744764	rs150365829	201723695	763	2.37E-06
RNASeq	PPIL3	201744764	rs79751905	201723140	763	2.37E-06
RNASeq	PPIL3	201744764	rs74943274	201717014	763	2.37E-06
RNASeq	PPIL3	201744764	rs16836588	201886358	757	2.83E-06

eQTL 2q33 BC241

**Probes**	**Gene**	**ePos**	**SNP**	**mPos**	**N**	P-value
A_23_P28213	PPIL3	201744763.5	rs2136600	201742345	241	3.18E-20
A_23_P28213	PPIL3	201744763.5	rs7606251	201736734	241	1.07E-18
A_23_P28213	PPIL3	201744763.5	rs7559150	201754063	239	1.07E-18
A_23_P28213	PPIL3	201744763.5	rs3888610	201732117	240	1.28E-18
A_23_P28213	PPIL3	201744763.5	rs6716122	201786474	240	2.10E-18
A_23_P28213	PPIL3	201744763.5	rs7917	201768238	241	2.48E-18
A_23_P28213	PPIL3	201744763.5	rs10172647	201778950	240	2.78E-18
A_23_P28213	PPIL3	201744763.5	chr2:201787159:I	201787159	240	2.78E-18
A_23_P28213	PPIL3	201744763.5	rs7588993	201763866	241	3.68E-18
A_23_P28213	PPIL3	201744763.5	rs6435066	201766023	241	3.68E-18
A_23_P28213	PPIL3	201744763.5	rs11894842	201773545	241	3.68E-18
A_23_P28213	PPIL3	201744763.5	rs13412214	201774182	241	3.68E-18
A_23_P28213	PPIL3	201744763.5	rs13412430	201774355	241	3.68E-18
A_23_P28213	PPIL3	201744763.5	rs13384245	201760937	241	9.84E-18
A_23_P28213	PPIL3	201744763.5	rs10204787	201762147	241	9.84E-18
A_23_P28213	PPIL3	201744763.5	rs113388793	201771341	241	9.84E-18
A_23_P28213	PPIL3	201744763.5	rs6754564	201779932	241	9.84E-18
A_23_P28213	PPIL3	201744763.5	rs2307358	201785657	241	9.84E-18
A_23_P28213	PPIL3	201744763.5	rs2307362	201785837	241	9.84E-18
A_23_P28213	PPIL3	201744763.5	rs4035022	201744352	241	6.17E-17
A_23_P28213	PPIL3	201744763.5	rs6747253	201748415	241	6.17E-17
A_23_P28213	PPIL3	201744763.5	rs13421776	201760902	241	1.41E-16
A_23_P28213	PPIL3	201744763.5	chr2:201762893:D	201762893	241	1.41E-16
A_23_P28213	PPIL3	201744763.5	rs4381763	201776988	241	1.41E-16
A_23_P28213	PPIL3	201744763.5	rs73059141	201788793	241	1.41E-16
A_23_P28213	PPIL3	201744763.5	rs10183261	201797676	241	1.41E-16
A_23_P28213	PPIL3	201744763.5	rs73059115	201730590	241	4.88E-16
A_23_P28213	PPIL3	201744763.5	rs3851973	201732878	241	4.88E-16
A_23_P28213	PPIL3	201744763.5	rs11892119	201736112	241	4.88E-16
A_23_P28213	PPIL3	201744763.5	rs7562391	201736166	241	4.88E-16
A_23_P28213	PPIL3	201744763.5	rs111976464	201735042	241	1.09E-15
A_23_P28213	PPIL3	201744763.5	rs6754769	201749852	237	1.33E-15
A_23_P28213	PPIL3	201744763.5	chr2:201793024:D	201793024	236	1.69E-15
A_23_P28213	PPIL3	201744763.5	rs4622700	201793113	238	1.70E-15
A_23_P28213	PPIL3	201744763.5	rs9288314	201794173	238	1.70E-15
A_23_P28213	PPIL3	201744763.5	chr2:201746389:I	201746389	241	2.04E-15
A_23_P28213	PPIL3	201744763.5	chr2:201735706:I	201735706	239	1.04E-14
A_23_P28213	PPIL3	201744763.5	rs138539278	201734921	238	1.13E-14
A_23_P28213	PPIL3	201744763.5	rs111315781	201738724	233	1.22E-14
A_23_P28213	PPIL3	201744763.5	rs73059116	201730687	238	2.20E-14
A_23_P28213	PPIL3	201744763.5	rs57210488	201706347	232	3.44E-14
A_23_P28213	PPIL3	201744763.5	rs13416500	201771798	233	5.09E-14
A_23_P28213	PPIL3	201744763.5	rs13404596	201771801	233	5.09E-14
A_23_P28213	PPIL3	201744763.5	chr2:201731757:D	201731757	236	1.61E-13
A_23_P28213	PPIL3	201744763.5	rs13406545	201792123	229	5.74E-13
A_23_P28213	PPIL3	201744763.5	c2_pos201472314	201764069	241	2.38E-08
A_24_P925996	IPO9	201825854.5	rs114537620	201601266	234	7.48E-08
A_24_P925996	IPO9	201825854.5	rs149731773	201714473	240	2.79E-07
A_24_P925996	IPO9	201825854.5	rs139246970	201704622	239	2.95E-07

A_24_P925996	IPO9	201825854.5	rs140802871	201738892	239	4.48E-07
A_23_P28213	PPIL3	201744763.5	rs77356501	201650853	233	1.79E-06
A_24_P924389	BZW1	201682738	rs193292207	201694677	238	2.30E-06
A_24_P924389	BZW1	201682738	rs139613089	201700440	238	2.30E-06
A_23_P28213	PPIL3	201744763.5	rs75609538	201712263	232	2.79E-06
A_24_P925996	IPO9	201825854.5	rs116023898	201648818	239	2.83E-06
A_24_P924389	BZW1	201682738	rs6725741	201626901	241	4.14E-06
A_24_P924389	BZW1	201682738	rs115642501	201631318	241	4.14E-06

eQTL 2q33 NB116

Probe	Gene	ePos	SNP	mPos	N	P-value
A_24_P3045	CASP10	202067001.5	rs149692156	201370268	115	1.43E-06
A_23_P349343	ALS2CR12	202187623.5	rs17448436	201462331	112	2.89E-06
A_23_P349343	ALS2CR12	202187623.5	rs113661102	201532836	111	3.01E-06
A_24_P713668	BZW1	201682738	rs183997345	201689544	116	3.39E-06
A_24_P713668	BZW1	201682738	rs77512390	201693141	116	3.39E-06
A_23_P415033	ARL8A	202108201	rs866208	201153257	116	9.45E-06
A_23_P28213	PPIL3	201744763.5	rs2136600	201742345	116	1.52E-05
A_23_P28213	PPIL3	201744763.5	rs7559150	201754063	115	2.42E-05
A_23_P149668	KIF14	200555243	rs17420160	201038501	111	5.15E-05
A_23_P28213	PPIL3	201744763.5	rs3888610	201732117	116	5.39E-05
A_23_P28213	PPIL3	201744763.5	rs7606251	201736734	116	5.39E-05
A_24_P257022	TNNT2	201337481.5	rs146502897	201145116	113	9.69E-05
A_23_P62959	PHLDA3	201436460	rs56118392	201782911	113	9.96E-05
A_23_P62959	PHLDA3	201436460	rs55835025	201783209	113	9.96E-05
A_23_P62959	PHLDA3	201436460	rs145470488	201783995	113	9.96E-05
A_23_P62959	PHLDA3	201436460	rs41302537	201793222	113	9.96E-05
A_32_P58913	BZW1	201682738	rs111766669	201579239	112	1.08E-04
A_23_P23296	PKP1	201277350	rs149429937	201142729	114	1.13E-04
A_23_P415033	ARL8A	202108201	rs1096598	201126552	114	1.16E-04
A_23_P28213	PPIL3	201744763.5	rs6716122	201786474	115	1.17E-04
A_24_P68649	RNPEP	201963520	rs3753969	201125551	116	1.29E-04
A_24_P68649	RNPEP	201963520	rs10920134	201117812	116	1.30E-04
A_24_P68649	RNPEP	201963520	rs58626409	201119643	116	1.30E-04
A_23_P28213	PPIL3	201744763.5	rs7588993	201763866	116	1.39E-04
A_23_P28213	PPIL3	201744763.5	rs6435066	201766023	116	1.39E-04
A_23_P28213	PPIL3	201744763.5	rs7917	201768238	116	1.39E-04
A_23_P28213	PPIL3	201744763.5	rs11894842	201773545	116	1.39E-04
A_23_P28213	PPIL3	201744763.5	rs13412214	201774182	116	1.39E-04
A_23_P28213	PPIL3	201744763.5	rs13412430	201774355	116	1.39E-04
A_23_P28213	PPIL3	201744763.5	rs10172647	201778950	116	1.39E-04
A_23_P28213	PPIL3	201744763.5	chr2:201787159:I	201787159	116	1.39E-04
A_23_P138271	ARL8A	202108201	rs77509836	201142646	114	1.78E-04
A_23_P349343	ALS2CR12	202187623.5	rs76358398	201601512	115	2.85E-04
A_23_P28213	PPIL3	201744763.5	rs13406545	201792123	111	3.01E-04
A_23_P39616	ORC2	201801658.5	c2_pos201472314	201764069	116	3.28E-04
A_23_P126888	KIF21B	200965670.5	rs9658867	201209117	112	3.40E-04
A_32_P58913	BZW1	201682738	rs13001274	201091838	114	3.41E-04
A_32_P58913	BZW1	201682738	chr2:201284458:D	201284458	114	3.41E-04
A_32_P58913	BZW1	201682738	chr2:201070317:I	201070317	113	3.68E-04
A_23_P28213	PPIL3	201744763.5	rs6754769	201749852	115	3.77E-04

eProbe: expression array probe for candidate gene; eGene: candidate gene; ePos: Location of candidate gene; mPosHG19: Location of SNP. Genome build GRCh37.13; N: number of samples assessed;
P-value: chi-square significance of association between SNP and gene expression.

Supplementary Table 21. Probe sequences for EMSA analyses.

Probe name	Probe sequence
rs201376807_MajorCC	CGGAGTTTCACTCTTGTTGC***CC***GGAGTATAATGGCGCGATCT
rs201376807_MajorGG	AGATCGCGCCATTATACTCC***GG***GCAACAAGAGTGAAACTCCG
rs201376807_minor1F	CGGAGTTTCACTCTTGTTGC***C*CAGAG*C***GGAGTATAATGGCGCGATCT
rs201376807_minor1R	AGATCGCGCCATTATACTCC***GCTCTGG***GCAACAAGAGTGAAACTCCG
rs4407214_Major T	TTGCTTCCGGACCTTCCCCCTCCTGCCCCCCCTTTGGGTGC
rs4407214_Major A	GCACCCAAAGGGGGGGCAGGAGGGGGAAGGTCCGGAAGCAA
rs4407214_minor G	TTGCTTCCGGACCTTCCCCCGCCTGCCCCCCCTTTGGGTGC
rs4407214_minor C	GCACCCAAAGGGGGGGCAGGCGGGGGAAGGTCCGGAAGCAA
rs67073037_MajorA	TTTCCTAATCTCATATTAAA***A***GTCATAGTTTTGGATTATGT
rs67073037_MajorT	ACATAATCCAAAACTATGAC***T***TTTAATATGAGATTAGGAAA
rs67073037_minorT	TTTCCTAATCTCATATTAAA***T***GTCATAGTTTTGGATTATGT
rs67073037_minorA	ACATAATCCAAAACTATGAC***A***TTTAATATGAGATTAGGAAA
rs67873458_MajorT	TAGGTTTCAGCATCGTTTTA***T***CTGCACTGTCTCCTTTTAAC
rs67873458_MajorA	GTTAAAAGGAGACAGTGCAG***A***TAAAACGATGCTGAAACCTA
rs67873458_minorC	TAGGTTTCAGCATCGTTTTA***C***CTGCACTGTCTCCTTTTAAC
rs67873458_minorG	GTTAAAAGGAGACAGTGCAG***G***TAAAACGATGCTGAAACCTA
rs66604446_MajorC	CCTCTAGAGTCATGGTGCCC***C***GAACTCCTGGTCAAGTGCCC
rs66604446_MajorG	GGGCACTTGACCAGGAGTTC***G***GGGCACCATGACTCTAGAGG
rs66604446_minorT	CCTCTAGAGTCATGGTGCCC***T***GAACTCCTGGTCAAGTGCCC
rs66604446_minorA	GGGCACTTGACCAGGAGTTC***A***GGGCACCATGACTCTAGAGG
rs6721781_MajorG	ACATTCCCTACTTTGAGGGT***G***TGTCCTCTTGGAGCATCAGT
rs6721781_MajorC	ACTGATGCTCCAAGAGGACA***C***ACCCTCAAAGTAGGGAATGT
rs6721781_minorA	ACATTCCCTACTTTGAGGGT***A***TGTCCTCTTGGAGCATCAGT
rs6721781_minorT	ACTGATGCTCCAAGAGGACA***T***ACCCTCAAAGTAGGGAATGT
rs66768547_MajorA	ACTTGCACATATATTATGGT*A*TAAAATTAAAAAGTCATATG
rs66768547_MajorT	CATATGACTTTTTAATTTTA***T***ACCATAATATATGTGCAAGT
rs66768547_minorG	ACTTGCACATATATTATGGT*G*TAAAATTAAAAAGTCATATG
rs66768547_minorC	CATATGACTTTTTAATTTTA***C***ACCATAATATATGTGCAAGT
rs4577244_MajorC	GTTTTTAGAGGGTTTTTGTA***C***GTTATGTAGCTGAGCATTCC
rs4577244_MajorG	GGAATGCTCAGCTACATAAC***G***TACAAAAACCCTCTAAAAAC
rs4577244_minorT	GTTTTTAGAGGGTTTTTGTA***T***GTTATGTAGCTGAGCATTCC
rs4577244_minorA	GGAATGCTCAGCTACATAAC***A***TACAAAAACCCTCTAAAAAC
rs4438440_MajorG	TCAGAGTACTACTAGCCGTG***G***GAAGTTTCTCTGTGAAGCTT
rs4438440_MajorC	AAGCTTCACAGAGAAACTTC***C***CACGGCTAGTAGTACTCTGA
rs4438440_minorC	TCAGAGTACTACTAGCCGTG***C***GAAGTTTCTCTGTGAAGCTT
rs4438440_minorG	AAGCTTCACAGAGAAACTTC***G***CACGGCTAGTAGTACTCTGA
rs11689719_MajorG	TCCTTGGGGTATTCACTTCT***G***ATGCCTTCAGGAATTGAGAT
rs11689719_MajorC	ATCTCAATTCCTGAAGGCAT***C***AGAAGTGAATACCCCAAGGA
rs11689719_minorT	TCCTTGGGGTATTCACTTCT***T***ATGCCTTCAGGAATTGAGAT
rs11689719_minorA	ATCTCAATTCCTGAAGGCAT***A***AGAAGTGAATACCCCAAGGA
rs4666140_MajorC	CGTTTAAAAATTCTGTTGAA***C***TTTAAAAAATAAATGTGAAA
rs4666140_MajorG	TTTCACATTTATTTTTTAAA***G***TTCAACAGAATTTTTAAACG
rs4666140_minorT	CGTTTAAAAATTCTGTTGAA***T***TTTAAAAAATAAATGTGAAA
rs4666140_minorA	TTTCACATTTATTTTTTAAA***A***TTCAACAGAATTTTTAAACG
rs11677283_MajorC	CCCAAAATCCTTTGTGGTTA***C***TGTACATGTCTGTGCAGAGT
rs11677283_MajorG	ACTCTGCACAGACATGTACA***G***TAACCACAAAGGATTTTGGG
rs11677283_minorT	CCCAAAATCCTTTGTGGTTA***T***TGTACATGTCTGTGCAGAGT
rs11677283_minorA	ACTCTGCACAGACATGTACA***A***TAACCACAAAGGATTTTGGG

rs35617956_majorAT	ATGCCACTATGCCTGGCTA*AT*TTTTTTATAGAGACAGAGT
rs35617956_MajorAT	ACTCTGTCTCTATAAAAAA*AT*TAGCCAGGCATAGTGGCAT
rs35617956_minor2F	ATGCCACTATGCCTGGCTA*ATT*TTTTTTATAGAGACAGAGT
rs35617956_minor2R	ACTCTGTCTCTATAAAAAA*AAT*TAGCCAGGCATAGTGGCAT
rs4666144_MajorC	TGGCTTTTCCAGTGCTCCTC*C*TGTGACCATTTGTGATCATG
rs4666144_MajorG	CATGATCACAAATGGTCACA*G*GAGGAGCACTGGAAAAGCCA
rs4666144_minorT	TGGCTTTTCCAGTGCTCCTC*T*TGTGACCATTTGTGATCATG
rs4666144_minorT	CATGATCACAAATGGTCACA*A*GAGGAGCACTGGAAAAGCCA
rs4666151_MajorC	GTCTTGTGAGATGAGGCTCTCACTTGGGATTCCCACACACA
rs4666151_MajorG	TGTGTGTGGGAATCCCAAGTGAGAGCCTCATCTCACAAGAC
rs4666151_minorA	GTCTTGTGAGATGAGGCTCTAACTTGGGATTCCCACACACA
rs4666151_minorT	TGTGTGTGGGAATCCCAAGTTAGAGCCTCATCTCACAAGAC
rs72786123_MajorA	CACGATCTCGGCTCACTGCAACCTCCACCTCCCAGGTTCAA
rs72786123_MajorT	TTGAACCTGGGAGGTGGAGG*T*TGCAGTGAGCCGAGATCGTG
rs72786123_minorG	CACGATCTCGGCTCACTGCAGCCTCCACCTCCCAGGTTCAA
rs72786123_minorC	TTGAACCTGGGAGGTGGAGG*C*TGCAGTGAGCCGAGATCGTG

Nucleotide alterations representing major and minor alleles of each SNP are italicized in red.

SUPPLEMENTARY NOTE

FUNDING and ACKNOWLEDGEMENTS

ABCFS

The Australian Breast Cancer Family Study (ABCFS) was supported by grant UM1 CA164920 from the National Cancer Institute (USA). The content of this manuscript does not necessarily reflect the views or policies of the National Cancer Institute or any of the collaborating centers in the Breast Cancer Family Registry (BCFR), nor does mention of trade names, commercial products, or organizations imply endorsement by the USA Government or the BCFR. The ABCFS was also supported by the National Health and Medical Research Council of Australia, the New South Wales Cancer Council, the Victorian Health Promotion Foundation (Australia) and the Victorian Breast Cancer Research Consortium. J.L.H. is a National Health and Medical Research Council (NHMRC) Australia Fellow and a Victorian Breast Cancer Research Consortium Group Leader. M.C.S. is a NHMRC Senior Research Fellow and a Victorian Breast Cancer Research Consortium Group Leader. The ABCFS wishes to thank Maggie Angelakos, Judi Maskiell, and Gillian Dite.

ABCS

The ABCS study was supported by the Dutch Cancer Society [grants NKI 2007-3839; 2009 4363]; BBMRI-NL, which is a Research Infrastructure financed by the Dutch government (NWO 184.021.007); and the Dutch National Genomics Initiative. The ABCS would like to thank Sten Cornelissen, Richard van Hien, Linde Braaf, Senno Verhoef, Laura van't Veer, C Ellen van der Schoot, and Femke Atsma.

ABCTB

The Australian Breast Cancer Tissue Bank is generously supported by the National Health and Medical Research Council of Australia, The Cancer Institute NSW and the National Breast Cancer Foundation. The ABCTB investigators wish to thank Christine Clarke, Rosemary Balleine, Robert Baxter, Stephen Braye, Jane Carpenter, Jane Dahlstrom, John Forbes, Soon Lee, Debbie Marsh, Adrienne Morey, Nirmala Pathmanathan, Rodney Scott, Allan Spigelman, Nicholas Wilcken, and Desmond Yip. Samples are made available to researchers on a non-exclusive basis.

BBCC

The work of the BBCC was partly funded by ELAN-Fond of the University Hospital of Erlangen.

SUPPLEMENTARY NOTE

BBCS

The BBCS is funded by Cancer Research UK and Breakthrough Breast Cancer and acknowledges NHS funding to the NIHR Biomedical Research Centre, and the National Cancer Research Network (NCRN). They wish to thank Eileen Williams, Elaine Ryder-Mills, and Kara Sargus.

BCAC

BCAC is funded by Cancer Research UK [C1287/A10118, C1287/A12014] and by the European Community´s Seventh Framework Programme under grant agreement number 223175 (grant number HEALTH-F2-2009-223175) (COGS). BCAC would like to thank all the individuals who took part in these studies and all the researchers, clinicians, technicians and administrative staff who have enabled this work to be carried out.

BCFR

The Australia, California, and Ontario sites of the Breast Cancer Family Registry were supported by grant UM1 CA164920 from the National Cancer Institute (USA). The content of this manuscript does not necessarily reflect the views or policies of the National Cancer Institute or any of the collaborating centers in the Breast Cancer Family Registry (BCFR), nor does mention of trade names, commercial products, or organizations imply endorsement by the USA Government or the BCFR. BCFR-AU thanks Maggie Angelakos, Judi Maskiell, Gillian Dite, and Helen Tsimiklis. BCFR-NY would like to thank members and participants in the New York site of the Breast Cancer Family Registry for their contributions to the study. BDFR-ON wishes to thank members and participants in the Ontario Familial Breast Cancer Registry for their contributions to the study.

BFBOCC-LT

BFBOCC-LT is partly supported by Research Council of Lithuania grant LIG-07/2012. They would like to acknowledge Vilius Rudaitis, Laimonas Griškevičius, and Ramūnas Janavičius.

BFBOCC-LV

BFBOCC-LV acknowledges Drs. Janis Eglitis, Anna Krilova and and Aivars Stengrevics.

BIDMC

BIDMC is supported by the Breast Cancer Research Foundation.

BIGGS

ES is supported by NIHR Comprehensive Biomedical Research Centre, Guy's & St. Thomas' NHS Foundation Trust in partnership with King's College London, United Kingdom. IT is supported by the Oxford Biomedical Research Centre. BIGGS would like to thank Michael Kerin, Nicola Miller, Niall McInerney, and Gabrielle Colleran.

SUPPLEMENTARY NOTE

BMBSA

BRCA-gene mutations and breast cancer in South African women (BMBSA) was supported by grants from the Cancer Association of South Africa (CANSA) to Elizabeth J. van Rensburg. They wish to thank the families who contribute to the BMBSA study.

BPC3

The BPC3 was supported by the US National Institutes of Health, National Cancer Institute under cooperative agreements U01-CA98233 (NHS, NHSII, WHS), U01-CA98710 (CPS2), U01-CA98216 (EPIC), U01-CA98758 (MEC) and Intramural Research Program of NIH/National Cancer Institute, Division of Cancer Epidemiology and Genetics (PLCO). The authors thank Drs Christine Berg and Philip Prorok, Division of Cancer Prevention, NCI, the screening center investigators and staff of the PLCO Cancer Screening Trial, Mr Thomas Riley and staff at Information Management Services, Inc., and Ms Barbara O'Brien and staff at Westat, Inc. for their contributions to the PLCO Cancer Screening Trial. We would like to thank the participants and staff of the NHS and NHSII for their valuable contributions as well as the following state cancer registries for their help: AL, AZ, AR, CA, CO, CT, DE, FL, GA, ID, IL, IN, IA, KY, LA, ME, MD, MA, MI, NE, NH, NJ, NY, NC, ND, OH, OK, OR, PA, RI, SC, TN, TX, VA, WA, WY. The authors assume full responsibility for analyses and interpretation of these data.

BRICOH

SLN was partially supported by the Morris and Horowitz Families Endowed Professorship. BRICOH wishes to thank Yuan Chun Ding and Linda Steele for their work in participant enrollment and biospecimen and data management.

BSUCH

The BSUCH study was supported by the Dietmar-Hopp Foundation, the Helmholtz Society and the German Cancer Research Center (DKFZ). They wish to acknowledge Peter Bugert and Medical Faculty Mannheim.

CBCS

This work was supported by the NEYE Foundation.

CECILE

The CECILE study was funded by Fondation de France, Institut National du Cancer (INCa), Ligue Nationale contre le Cancer, Ligue contre le Cancer Grand Ouest, Agence Nationale de Sécurité Sanitaire (ANSES), Agence Nationale de la Recherche (ANR).

CGEMS

The Nurses’ Health Studies are supported by NIH grants CA65725, CA87969, CA49449, CA67262, CA50385 and 5UO1CA098233.

SUPPLEMENTARY NOTE

CGPS

The CGPS was supported by the Chief Physician Johan Boserup and Lise Boserup Fund, the Danish Medical Research Council, and Herlev Hospital. CGPS wishes to thank staff and participants of the Copenhagen General Population Study and Dorthe Uldall Andersen, Maria Birna Arnadottir, Anne Bank, and Dorthe Kjeldgård Hansen for their excellent technical assistance.

CIMBA CORE

The CIMBA data management and data analysis were supported by Cancer Research – UK grants C12292/A11174 and C1287/A10118. SH is supported by an NHMRC Program Grant to GCT. ACA is a Cancer Research -UK Senior Cancer Research Fellow. GCT is an NHMRC Senior Principal Research Fellow.

CNIO

This work was partially supported by Spanish Association against Cancer (AECC08), RTICC 06/0020/1060, FISPI08/1120, Mutua Madrileña Foundation (FMMA) and SAF2010-20493. They wish to thank Alicia Barroso, Rosario Alonso, and Guillermo Pita for their assistance.

CNIO-BCS

The CNIO-BCS was supported by the Genome Spain Foundation, the Red Temática de Investigación Cooperativa en Cáncer and grants from the Asociación Española Contra el Cáncer and the Fondo de Investigación Sanitario (PI11/00923 and PI081120). The Human Genotyping-CEGEN Unit (CNIO) is supported by the Instituto de Salud Carlos III.

COH-CCGCRN

City of Hope Clinical Cancer Genetics Community Network and the Hereditary Cancer Research Registry was supported in part by Award Number RC4CA153828 (PI: J. Weitzel) from the National Cancer Institute and the Office of the Director, National Institutes of Health. The content is solely the responsibility of the authors and does not necessarily represent the official views of the National Institutes of Health.

CONSIT TEAM and MBCSG

CONSiT TEAM and MBCSG studies acknowledge the contribution of Paolo Peterlongo of the Istituto FIRC di Oncologia Molecolare, Milan, Italy; Daniela Zaffaroni and Giulietta Scuvera of the Fondazione IRCCS Istituto Nazionale dei Tumori, Milano, Italy; Irene Feroce of the Istituto Europeo di Oncologia, Milan, Italy; Loris Bernard and the personnnel of the Cogentech Cancer Genetic Test Laboratory, Milan, Italy; Alessandra Viel of the CRO Aviano National Cancer Institute, Aviano (PN), Italy; Liliana Varesco and Viviana Gismondi of IRCCS AOU San Martino - IST Istituto Nazionale per la Ricerca sul Cancro, Genoa, Italy; Laura Papi of the University of Florence, Florence, Italy; Laura Ottini and Giuseppe Giannini of the "Sapienza" University, Rome, Italy; Antonella Savarese and Aline Martayan of the Istituto Nazionale Tumori Regina Elena, Rome, Italy; Stefania Tommasi and Brunella Pilato of the Istituto Nazionale Tumori "Giovanni Paolo II", Bari, Italy.

SUPPLEMENTARY NOTE

CTS

The CTS was initially supported by the California Breast Cancer Act of 1993 and the California Breast Cancer Research Fund (contract 97-10500) and is currently funded through the National Institutes of Health (R01 CA77398). Collection of cancer incidence data was supported by the California Department of Public Health as part of the statewide cancer reporting program mandated by California Health and Safety Code Section 103885. HAC receives support from the Lon V Smith Foundation (LVS39420). The CTS Steering Committee includes Leslie Bernstein, Susan Neuhausen, James Lacey, Sophia Wang, Huiyan Ma, Yani Lu, and Jessica Clague DeHart at the Beckman Research Institute of City of Hope, Dennis Deapen, Rich Pinder, Eunjung Lee, and Fred Schumacher at the University of Southern California, Pam Horn-Ross, Peggy Reynolds, Christina Clarke Dur and David Nelson at the Cancer Prevention Institute of California, and Hoda Anton-Culver, Argyrios Ziogas, and Hannah Park at the University of California Irvine.

DEMOKRITOS

This research has been co-financed by the European Union (European Social Fund – ESF) and Greek national funds through the Operational Program "Education and Lifelong Learning" of the National Strategic Reference Framework (NSRF) - Research Funding Program of the General Secretariat for Research & Technology: ARISTEIA. Investing in knowledge society through the European Social Fund.

DKFZ

The DKFZ study was supported by the DKFZ.

ESTHER

The ESTHER study was supported by a grant from the Baden Württemberg Ministry of Science, Research and Arts. Additional cases were recruited in the context of the VERDI study, which was supported by a grant from the German Cancer Aid (Deutsche Krebshilfe). They wish to thank Hartwig Ziegler, Sonja Wolf, Volker Hermann, Christa Stegmaier, Aida Karina Dieffenbach, and Katja Butterbach.

FCCC

The FCCC authors acknowledge support from The University of Kansas Cancer Center (P30 CA168524) and the Kansas Bioscience Authority Eminent Scholar Program. A.K.G. was funded by 5U01CA113916, R01CA140323, and by the Chancellors Distinguished Chair in Biomedical Sciences Professorship. The FCCC thanks Ms. JoEllen Weaver and Dr. Betsy Bove for their technical support.

GC-HBOC

The German Consortium of Hereditary Breast and Ovarian Cancer (GC-HBOC) is supported by the German Cancer Aid (grant no 109076, Rita K. Schmutzler) and by the Center for Molecular Medicine Cologne (CMMC).

GENICA

SUPPLEMENTARY NOTE

The GENICA was funded by the Federal Ministry of Education and Research (BMBF) Germany grants 01KW9975/5, 01KW9976/8, 01KW9977/0 and 01KW0114, the Robert Bosch Foundation, Stuttgart, Deutsches Krebsforschungszentrum (DKFZ), Heidelberg, the Institute for Prevention and Occupational Medicine of the German Social Accident Insurance, Institute of the Ruhr University Bochum (IPA), Bochum, as well as the Department of Internal Medicine, Evangelische Kliniken Bonn gGmbH, Johanniter Krankenhaus, Bonn, Germany. The GENICA Network: Dr. Margarete Fischer-Bosch-Institute of Clinical Pharmacology, Stuttgart, and University of Tübingen, Germany [HB, Wing-Yee Lo, Christina Justenhoven], German Cancer Consortium (DKTK) and German Cancer Research Center (DKFZ) [HB], Department of Internal Medicine, Evangelische Kliniken Bonn gGmbH, Johanniter Krankenhaus, Bonn, Germany [YDK, Christian Baisch], Institute of Pathology, University of Bonn, Germany [Hans-Peter Fischer], Molecular Genetics of Breast Cancer, Deutsches Krebsforschungszentrum (DKFZ), Heidelberg, Germany [Ute Hamann], Institute for Prevention and Occupational Medicine of the German Social Accident Insurance, Institute of the Ruhr University Bochum (IPA), Bochum, Germany [Thomas Brüning, Beate Pesch, Sylvia Rabstein, Anne Lotz]; and Institute of Occupational Medicine and Maritime Medicine, University Medical Center Hamburg-Eppendorf, Germany [Volker Harth].

GEORGETOWN

CI received support from the Non-Therapeutic Subject Registry Shared Resource at Georgetown University (NIH/NCI grant P30-CA051008), the Fisher Center for Familial Cancer Research, and Swing Fore the Cure.

G-FAST

Kim De Leeneer is supported by GOA grant BOF10/GOA/019 (Ghent University) and spearhead financing of Ghent University Hospital

GOG

This study was supported by National Cancer Institute grants to the Gynecologic Oncology Group (GOG) Administrative Office and Tissue Bank (CA 27469), the GOG Statistical and Data Center (CA 37517), and GOG's Cancer Prevention and Control Committee (CA 101165). Drs. Greene, Mai and Savage were supported by funding from the Intramural Research Program, NCI. GOG wishes to thank the investigators of the Australia New Zealand Gynaecological Oncology Group (ANZGOG).

HCSC

HCSC was supported by a grant RD12/00369/0006 and 12/00539 from ISCIII (Spain), partially supported by European Regional Development FEDER funds. They acknowledge Alicia Tosar and Paula Diaque for their assistance.

HEBCS

The HEBCS was financially supported by the Helsinki University Central Hospital Research Fund, Academy of Finland (266528), the Finnish Cancer Society, The Nordic Cancer Union, and the Sigrid Juselius Foundation. HEBCS would like to thank Dr. Karl von Smitten and RN Irja Erkkilä for their help with the HEBCS data and samples.

SUPPLEMENTARY NOTE

HMBCS

The HMBCS was supported by a grant from the Friends of Hannover Medical School and by the Rudolf Bartling Foundation. They thank Natalia Antonenkova, Peter Hillemanns, Hans Christiansen, and Johann H. Karstens

HRBCP

HRBCP is supported by The Hong Kong Hereditary Breast Cancer Family Registry and the Dr. Ellen Li Charitable Foundation, Hong Kong. They wish to thank Hong Kong Sanatoriuma and Hospital for their continual support.

HUNBOCS

Hungarian Breast and Ovarian Cancer Study was supported by Hungarian Research Grants KTIA-OTKA CK-80745 and OTKA K-112228. HUNBOCS wishes to thank the Hungarian Breast and Ovarian Cancer Study Group members (Janos Papp, Tibor Vaszko, Aniko Bozsik, Timea Pocza, Judit Franko, Maria Balogh, Gabriella Domokos, Judit Ferenczi, Department of Molecular Genetics, National Institute of Oncology, Budapest, Hungary) and the clinicians and patients for their contributions to this study.

HVH

The HVH would like to thank the Oncogenetics Group (VHIO), and the High Risk and Cancer Prevention Unit of the University Hospital Vall d'Hebron.

ICO

Contract grant sponsor: Asociación Española Contra el Cáncer, Spanish Health Research Fund; Carlos III Health Institute; Catalan Health Institute and Autonomous Government of Catalonia. Contract grant numbers: ISCIIIRETIC RD06/0020/1051, RD12/0036/008, PI10/01422, PI10/00748, PI13/00285, PIE13/00022, 2009SGR290 and 2014SGR364. They wish to thank the ICO Hereditary Cancer Program team led by Dr. Gabriel Capella.

IHCC

The IHCC was supported by Grant PBZ_KBN_122/P05/2004

ILUH

The ILUH group was supported by the Icelandic Association "Walking for Breast Cancer Research" and by the Landspitali University Hospital Research Fund.

INHERIT

This work was supported by the Canadian Institutes of Health Research for the "CIHR Team in Familial Risks of Breast Cancer" program, the Canadian Breast Cancer Research Alliance-grant #019511 and the Ministry of Economic Development, Innovation and Export Trade – grant # PSR-SIIRI-701. INHERIT would like to thank Dr. Martine Dumont, Martine Tranchant for sample

management and skillful technical assistance. J.S. is Chairholder of the Canada Research Chair in Oncogenetics. J.S. and P.S. were part of the QC and Genotyping coordinating group of iCOGS (BCAC and CIMBA).

IOVHBOCS

IOVHBOCS is supported by Ministero della Salute and “5x1000” Istituto Oncologico Veneto grant.

IPOBCS

This study was in part supported by Liga Portuguesa Contra o Cancro. IPOBCS wishes to thank Drs. Ana Peixoto, Catarina Santos, Patrícia Rocha and Pedro Pinto for their skillful contribution to the study.

KARBAC

Financial support for KARBAC was provided through the regional agreement on medical training and clinical research (ALF) between Stockholm County Council and Karolinska Institutet, the Swedish Cancer Society, The Gustav V Jubilee foundation, and Bert von Kantzows foundation.

KBCP

The KBCP was financially supported by the special Government Funding (EVO) of Kuopio University Hospital grants, Cancer Fund of North Savo, the Finnish Cancer Organizations, and by the strategic funding of the University of Eastern Finland. The KBCP wishes to thank Eija Myöhänen and Helena Kemiläinen.

KOHBRA

KOHBRA is supported by a grant from the National R&D Program for Cancer Control, Ministry for Health, Welfare and Family Affairs, Republic of Korea (1020350).

LMBC

LMBC is supported by the 'Stichting tegen Kanker' (232-2008 and 196-2010). Diether Lambrechts is supported by the FWO and the KULPFV/10/016-SymBioSysII. LMBC would like to acknowledge Gilian Peuteman, Dominiek Smeets, Thomas Van Brussel. and Kathleen Corthouts.

MARIE

The MARIE study was supported by the Deutsche Krebshilfe e.V. [70-2892-BR I], the Hamburg Cancer Society, the German Cancer Research Center and the genotype work in part by the Federal Ministry of Education and Research (BMBF) Germany [01KH0402]. MARIE would like to thank Lars Beckmann, Thomas Illig, Kirsten Mittelstraß for their valuable contributions to analysis and generation of the GWAS data, and S. Behrens, U. Eilber, and B. Kaspereit for their excellent technical assistance.

MAYO

SUPPLEMENTARY NOTE

MAYO is supported by NIH grants CA116167, CA128978 and CA176785, an NCI Specialized Program of Research Excellence (SPORE) in Breast Cancer (CA116201), a U.S. Department of Defence Ovarian Cancer Idea award (W81XWH-10-1-0341), a grant from the Breast Cancer Research Foundation, a generous gift from the David F. and Margaret T. Grohne Family Foundation and the Ting Tsung and Wei Fong Chao Foundation.

MCBCS

The MCBCS was supported by the NIH grants CA128978, CA116167, CA176785 an NIH Specialized Program of Research Excellence (SPORE) in Breast Cancer [CA116201], and the Breast Cancer Research Foundation and a generous gift from the David F. and Margaret T. Grohne Family Foundation and the Ting Tsung and Wei Fong Chao Foundation

MCCS

MCCS cohort recruitment was funded by VicHealth and Cancer Council Victoria. The MCCS was further supported by Australian NHMRC grants 209057, 251553 and 504711 and by infrastructure provided by Cancer Council Victoria.

MCGILL

Jewish General Hospital Weekend to End Breast Cancer, Quebec Ministry of Economic Development, Innovation and Export Trade.

MEC

The MEC was support by NIH grants CA63464, CA54281, CA098758 and CA132839.

MODSQUAD

MODSQUAD was supported by MH CZ - DRO (MMCI, 00209805) and by the European Regional Development Fund and the State Budget of the Czech Republic (RECAMO, CZ.1.05/2.1.00/03.0101) to LF, and by Charles University in Prague project UNCE204024 (MZ). Modifier Study of Quantitative Effects on Disease (MODSQUAD) acknowledges ModSQuaD members Lenka Foretova and Eva Machackova (Department of Cancer Epidemiology and Genetics, Masaryk Memorial Cancer Institute and MF MU, Brno, Czech Republic); and Michal Zikan, Petr Pohlreich and Zdenek Kleibl (Oncogynecologic Center and Department of Biochemistry and Experimental Oncology, First Faculty of Medicine, Charles University, Prague, Czech Republic).

MSKCC

MSKCC is supported by grants from the Breast Cancer Research Foundation, the Robert and Kate Niehaus Clinical Cancer Genetics Initiative, and the Andrew Sabin Research Fund. They would like to thank Anne Lincoln and Lauren Jacobs.

MTLGEBCS

The work of MTLGEBCS was supported by the Quebec Breast Cancer Foundation, the Canadian Institutes of Health Research for the “CIHR Team in Familial Risks of Breast Cancer”

program – grant # CRN-87521 and the Ministry of Economic Development, Innovation and Export Trade – grant # PSR-SIIRI-701. The MTLGEBCS would like to thank Martine Tranchant (CHU de Québec Research Center), Marie-France Valois, Annie Turgeon and Lea Heguy (McGill University Health Center, Royal Victoria Hospital; McGill University) for DNA extraction, sample management and skillful technical assistance. J.S. is Chairholder of the Canada Research Chair in Oncogenetics.

NAROD

NAROD is supported by R01 CA149429-01 from the National Institutes of Health.

NBCS

The NBCS was supported by grants from the Norwegian Research council, 155218/V40, 175240/S10 to ALBD, FUGE-NFR 181600/V11 to VNK and a Swizz Bridge Award to ALBD. NBCS includes the following clinical collaborators: Prof. Per Eystein Lønning, MD (Section of Oncology, Institute of Medicine, University of Bergen and Department of Oncology, Haukeland University Hospital, Bergen, Norway), Prof. Em. Sophie D. Fosså, MD (National Resource Centre for Long-term Studies after Cancer, Rikshospitalet-Radiumhospitalet Cancer Clinic Montebello, Oslo, Norway), Head physician Tone Ikdahl, MD (Department of Oncology, Oslo University Hospital, Oslo, Norway), Dr. Lars Ottestad, MD (Department of Genetics and Department of Oncology, Oslo University Hospital Radiumhospitalet), Dr. Marit Muri Holmen, MD (Department of Radiology, Oslo University Hospital Radiumhospitalet, Oslo, Norway), Dr. Vilde Haakensen, MD (Department of Genetics and Department of Oncology, Oslo University Hospital Radiumhospitalet and Institute for Clinical Medicine, Faculty of Medicine, University of Oslo, Oslo, Norway), Prof. Bjørn Naume, MD (Division of Cancer Medicine and Radiotherapy, Department of Oncology, Oslo University Hospital Radiumhospitalet, Oslo, Norway), Prof. Eiliv Lund (Department of Community Medicine, Faculty of Health Sciences, University of Tromsø - The Arctic University of Norway, Tromsø, Norway) , Assoc. Prof. Åslaug Helland, MD (Department of Genetics, Institute for Cancer Research and Department of Oncology, Oslo University Hospital Radiumhospitalet, Oslo, Norway and Institute of Clinical Medicine, Faculty of Medicine, University of Oslo, Oslo, Norway), Prof. Inger Torhild Gram, MD (Department of Community Medicine, Faculty of Health Sciences, University of Tromsø and Norwegian Centre for Integrated Care and Telemedicine, University Hospital of North Norway, Tromsø, Norway), Prof. Em. Rolf Kåresen, MD (Department of Breast and Endocrine Surgery, Institute for Clinical Medicine, Ullevaal Hospital, Oslo University Hospital and Institute of Clinical Medicine, Faculty of Medicine, University of Oslo, Oslo, Norway), Dr. Ellen Schlichting, MD (Department for Breast and Endocrine Surgery, Oslo University Hospital Ullevaal, Oslo, Norway), Prof. Toril Sauer, MD (Department of Pathology at Akershus University hospital, Lørenskog, Norway), Dr. Olav Engebråten, MD (Institute for Clinical Medicine, Faculty of Medicine, University of Oslo and Department of Oncology, Oslo University Hospital, Oslo, Norway), Dr. Margit Riis, MD (Department of Surgery, Akershus University Hospital and Department of Clinical Molecular Biology (EpiGen), Institute of Clinical Medicine, Akershus University Hospital, University of Oslo, Lørenskog, Norway).

NBHS

The NBHS was supported by NIH grant R01CA100374. Biological sample preparation was conducted the Survey and Biospecimen Shared Resource, which is supported by P30 CA68485. They wish to thank study participants and research staff for their contributions and commitment to this study.

SUPPLEMENTARY NOTE

NCI

The research of Drs. MH Greene and PL Mai was supported by the Intramural Research Program of the US National Cancer Institute, NIH, and by support services contracts NO2-CP-11019-50 and N02-CP-65504 with Westat, Inc, Rockville, MD.

NICCC

NICCC is supported by Clalit Health Services in Israel. Some of its activities are supported by the Israel Cancer Association and the Breast Cancer Research Foundation (BCRF), NY. They would like to thank the NICCC National Familial Cancer Consultation Service team led by Sara Dishon, the lab team led by Dr. Flavio Lejbkowicz, and the research field operations team led by Dr. Mila Pinchev.

NNPIO

This work has been supported by the Russian Federation for Basic Research (grants 13-04-92613, 14-04-93959 and 15-04-01744).

OBCS

The OBCS was supported by research grants from the Finnish Cancer Foundation, the Academy of Finland (grant number 250083, 122715 and Center of Excellence grant number 251314), the Finnish Cancer Foundation, the Sigrid Juselius Foundation, the University of Oulu, the University of Oulu Support Foundation and the special Governmental EVO funds for Oulu University Hospital-based research activities. OBCS thanks Arja Jukkola-Vuorinen, Mervi Grip, Saila Kauppila, Kari Mononen, and Meeri Otsukka for data collection and sample preparation.

OCGN

The OCGN wishes to thank members and participants in the Ontario Cancer Genetics Network for their contributions to the study.

OFBCR

The Ontario Familial Breast Cancer Registry (OFBCR) was supported by grant UM1 CA164920 from the National Cancer Institute (USA). The content of this manuscript does not necessarily reflect the views or policies of the National Cancer Institute or any of the collaborating centers in the Breast Cancer Family Registry (BCFR), nor does mention of trade names, commercial products, or organizations imply endorsement by the USA Government or the BCFR. They would like to thank Teresa Selander and Nayana Weerasooriya.

ORIGO

The ORIGO study was supported by the Dutch Cancer Society (RUL 1997-1505) and the Biobanking and Biomolecular Resources Research Infrastructure (BBMRI-NL CP16). ORIGO would like to thank E. Krol-Warmerdam, and J. Blom for patient accrual, administering questionnaires, and managing clinical information. The LUMC survival data were retrieved from the Leiden hospital-based cancer registry system (ONCDOC) with the help of Dr. J. Molenaar.

SUPPLEMENTARY NOTE

OSU CCG

OSUCCG is supported by the Ohio State University Comprehensive Cancer Center and the Stefanie Spielman Breast Cancer Fund. Leigha Senter, Kevin Sweet, Caroline Craven, and Michelle O'Conor were instrumental in accrual of study participants, ascertainment of medical records and database management. Samples were processed by the OSU Human Genetics Sample Bank.

PBCS

The PBCS was funded by Intramural Research Funds of the National Cancer Institute, Department of Health and Human Services, USA. They acknowledge Louise Brinton, Mark Sherman, Neonila Szeszenia-Dabrowska, Beata Peplonska, Witold Zatonski, Pei Chao, and Michael Stagner.

pKARMA

The pKARMA study was supported by Märit and Hans Rausings Initiative Against Breast Cancer and acknowledges the Swedish Medical Research Counsel.

POSH

The POSH cohort was established with support from Cancer Research UK C22524 and C1275/A11699. The support of Lorraine Durcan, Linda Haywood and Louise Jones has been invaluable in establishing the oestrogen receptor status of the POSH cases and Nikki Graham in managing the DNA samples for the studyRBCS

The RBCS was funded by the Dutch Cancer Society (DDHK 2004-3124, DDHK 2009-4318). The RBCS wishes to thank Petra Bos, Jannet Blom, Ellen Crepin, Elisabeth Huijskens, Annette Heemskerk, and the Erasmus MC Family Cancer Clinic.

RPCI

RPCI biospecimens and data are from the DataBank and Biorepository (DBBR), a cancer center support grant shared resource supported by National Institutes of Health (P30 CA016056-37)

SASBAC

The SASBAC study was supported by funding from the Agency for Science, Technology and Research of Singapore (A*STAR), the US National Institute of Health (NIH) and the Susan G. Komen Breast Cancer Foundation. SASBAC acknowledges the Swedish Medical Research Counsel.

SBCS

The SBCS was supported by Yorkshire Cancer Research S295, S299, S305PA and Sheffield Experimental Cancer Medicine Centre. The SBCS would like to acknowledge Sue Higham, Helen Cramp, Ian Brock, Sabapathy Balasubramanian, and Dan Connley

SEABASS

SUPPLEMENTARY NOTE

SEABASS was supported by the Ministry of Science, Technology and Innovation, Ministry of Higher Education (UM.C/HlR/MOHE/06) and Cancer Research Initiatives Foundation. They wish to thank Sue Higham, Helen Cramp, Ian Brock, Sabapathy Balasubramanian, and Dan Connley.

SKKDKFZS

SKKDKFZS is supported by the DKFZ. They would like to thank all study participants, clinicians, family doctors, researchers and technicians for their contributions and commitment to this study.

SMC

This project was partially funded through a grant by the Israel Cancer Association and the funding for the Israeli Inherited Breast Cancer Consortium. The SMC team wishes to acknowledge the assistance of the Meirav Comprehensice breast cancer center team at the Sheba Medical Center for assistance in this study.

SWE-BRCA

SWE-BRCA collaborators are supported by the Swedish Cancer Society. Swedish scientists participating as SWE-BRCA collaborators are: from Lund University and University Hospital: Åke Borg, Håkan Olsson, Helena Jernström, Karin Henriksson, Katja Harbst, Maria Soller, Ulf Kristoffersson; from Gothenburg Sahlgrenska University Hospital: Anna Öfverholm, Margareta Nordling, Per Karlsson, Zakaria Einbeigi; from Stockholm and Karolinska University Hospital: Anna von Wachenfeldt, Annelie Liljegren, Annika Lindblom, Brita Arver, Gisela Barbany Bustinza, Johanna Rantala; from Umeå University Hospital: Beatrice Melin, Christina Edwinsdotter Ardnor, Monica Emanuelsson; from Uppsala University: Hans Ehrencrona, Maritta Hellström Pigg, Richard Rosenquist; from Linköping University Hospital: Marie Stenmark-Askmalm, Sigrun Liedgren.

SZBCS

The SZBCS was supported by Grant PBZ_KBN_122/P05/2004

TNBCC

The TNBCC was supported by: a Specialized Program of Research Excellence (SPORE) in Breast Cancer (CA116201), a grant from the Breast Cancer Research Foundation, a generous gift from the David F. and Margaret T. Grohne Family Foundation, the Stefanie Spielman Breast Cancer fund and the OSU Comprehensive Cancer Center, the Hellenic Cooperative Oncology Group research grant (HR R_BG/04) and the Greek General Secretary for Research and Technology (GSRT) Program, Research Excellence II, the European Union (European Social Fund – ESF), and Greek national funds through the Operational Program "Education and Lifelong Learning" of the National Strategic Reference Framework (NSRF) - Robert Pilarski and Charles Shapiro were instrumental in the formation of the OSU Breast Cancer Tissue Bank. The TNBCC thanks the Human Genetics Sample Bank for processing of samples and providing OSU Columbus area control samples.

UCHICAGO

SUPPLEMENTARY NOTE

UCHICAGO is supported by NCI Specialized Program of Research Excellence (SPORE) in Breast Cancer (CA125183), R01 CA142996, 1U01CA161032 and by the Ralph and Marion Falk Medical Research Trust, the Entertainment Industry Fund National Women's Cancer Research Alliance and the Breast Cancer research Foundation. OIO is an ACS Clinical Research Professor. UCHICAGO would like to thank Cecilia Zvocec, Qun Niu, physicians, genetic counselors, research nurses and staff of the Cancer Risk Clinic for their contributions to this resource, and the many families who contribute to their program.

UCLA

Jonsson Comprehensive Cancer Center Foundation; Breast Cancer Research Foundation. UCLA thanks Joyce Seldon MSGC and Lorna Kwan, MPH for assembling the data for this study.

UCSF

UCSF Cancer Risk Program and Helen Diller Family Comprehensive Cancer Center. They would like to thank Dr. Robert Nussbaum and the following genetic counselors for participant recruitment: Beth Crawford, Kate Loranger, Julie Mak, Nicola Stewart, Robin Lee, Amie Blanco and Peggy Conrad. UCSF also thanks Ms. Salina Chan for her data management.

SUPPLEMENTARY NOTE

UK2

The UK2 GWAS was funded by Wellcome Trust and Cancer Research UK. It included samples collected through the FBCS study which is funded by Cancer Research UK [C8620/A8372]. It included control data obtained through the WTCCC which was funded by the Wellcom Trust.

UKBGS

The UKBGS is funded by Breakthrough Breast Cancer and the Institute of Cancer Research (ICR), London. ICR acknowledges NHS funding to the NIHR Biomedical Research Centre. The UKBGS thanks the study participants, study staff, and the doctors, nurses and other health care providers and health information sources who have contributed to the study.

UKFOCR

UKFOCR was supported by a project grant from CRUK to Paul Pharoah. They thank Susan Ramus, Carole Pye, Patricia Harrington and Eva Wozniak for their contributions towards the UKFOCR.

UPENN

National Institutes of Health (NIH) (R01-CA102776 and R01-CA083855; Breast Cancer Research Foundation; Susan G. Komen Foundation for the cure, Basser Research Center for BRCA

VFCTG

The VFCTG was supported by the Victorian Cancer Agency, Cancer Australia, and the National Breast Cancer Foundation. They wish to thank Geoffrey Lindeman, Marion Harris, and Martin Delatycki of the Victorian Familial Cancer Trials Group; Sarah Sawyer and Rebecca Driessen for assembling this data; and Ella Thompson for performing all DNA amplification.

WCP

The Women's Cancer Program (WCP) at the Samuel Oschin Comprehensive Cancer Institute is funded by the American Cancer Society Early Detection Professorship (SIOP-06-258-01-COUN).

<u>Contributing consortia</u>

AOCS

Financial support for the AOCS was provided by the United States Army Medical Research and Materiel Command [DAMD17-01-1-0729], Cancer Council Victoria, Queensland Cancer Fund, Cancer Council New South Wales, Cancer Council South Australia, The Cancer Foundation of Western Australia, Cancer Council Tasmania and the National Health and Medical Research Council of Australia (NHMRC; 400413, 400281, 199600). G.C.T. and P.W. are supported by the NHMRC. RB was a Cancer Institute NSW Clinical Research Fellow. AOCS is comprised of Georgia Chenevix-Trench, Jonathan Beesley, Kelly-Anne Phillips, Rosemary Balleine, Juliet French, Karen McCue, and Stacey Edwards.

SUPPLEMENTARY NOTE

EMBRACE

Epidemiological study of BRCA1 & BRCA2 mutation carriers (EMBRACE): Douglas F. Easton is the PI of the study. EMBRACE is supported by Cancer Research UK Grants C1287/A10118 and C1287/A11990. D. Gareth Evans and Fiona Lalloo are supported by an NIHR grant to the Biomedical Research Centre, Manchester. The Investigators at The Institute of Cancer Research and The Royal Marsden NHS Foundation Trust are supported by an NIHR grant to the Biomedical Research Centre at The Institute of Cancer Research and The Royal Marsden NHS Foundation Trust. Ros Eeles and Elizabeth Bancroft are supported by Cancer Research UK Grant C5047/A8385. EMBRACE Collaborating Centres are: Coordinating Centre, Cambridge: Debra Frost, Steve Ellis, Elena Fineberg, Radka Platte. North of Scotland Regional Genetics Service, Aberdeen: Zosia Miedzybrodzka, Helen Gregory. Northern Ireland Regional Genetics Service, Belfast: Patrick Morrison, Lisa Jeffers. West Midlands Regional Clinical Genetics Service, Birmingham: Trevor Cole, Kai-ren Ong, Jonathan Hoffman. South West Regional Genetics Service, Bristol: Alan Donaldson, Margaret James. East Anglian Regional Genetics Service, Cambridge: Marc Tischkowitz, Joan Paterson, Amy Taylor. Medical Genetics Services for Wales, Cardiff: Alexandra Murray, Mark T. Rogers, Emma McCann. St James's Hospital, Dublin & National Centre for Medical Genetics, Dublin: M. John Kennedy, David Barton. South East of Scotland Regional Genetics Service, Edinburgh: Mary Porteous, Sarah Drummond. Peninsula Clinical Genetics Service, Exeter: Carole Brewer, Emma Kivuva, Anne Searle, Selina Goodman, Kathryn Hill. West of Scotland Regional Genetics Service, Glasgow: Rosemarie Davidson, Victoria Murday, Nicola Bradshaw, Lesley Snadden, Mark Longmuir, Catherine Watt, Sarah Gibson, Eshika Haque, Ed Tobias, Alexis Duncan. South East Thames Regional Genetics Service, Guy's Hospital London: Louise Izatt, Chris Jacobs, Caroline Langman. North West Thames Regional Genetics Service, Harrow: Huw Dorkins. Leicestershire Clinical Genetics Service, Leicester: Julian Barwell. Yorkshire Regional Genetics Service, Leeds: Julian Adlard, Gemma Serra-Feliu. Cheshire & Merseyside Clinical Genetics Service, Liverpool: Ian Ellis, Catherine Houghton. Manchester Regional Genetics Service, Manchester: D Gareth Evans, Fiona Lalloo, Jane Taylor. North East Thames Regional Genetics Service, NE Thames, London: Lucy Side, Alison Male, Cheryl Berlin. Nottingham Centre for Medical Genetics, Nottingham: Jacqueline Eason, Rebecca Collier. Northern Clinical Genetics Service, Newcastle: Fiona Douglas, Oonagh Claber, Irene Jobson. Oxford Regional Genetics Service, Oxford: Lisa Walker, Diane McLeod, Dorothy Halliday, Sarah Durell, Barbara Stayner. The Institute of Cancer Research and Royal Marsden NHS Foundation Trust: Ros Eeles, Susan Shanley, Nazneen Rahman, Richard Houlston, Elizabeth Bancroft, Elizabeth Page, Audrey Ardern-Jones, Kelly Kohut, Jennifer Wiggins, Elena Castro, Emma Killick, Sue Martin, Gillian Rea, Anjana Kulkarni. North Trent Clinical Genetics Service, Sheffield: Jackie Cook, Oliver Quarrell, Cathryn Bardsley. South West Thames Regional Genetics Service, London: Shirley Hodgson, Sheila Goff, Glen Brice, Lizzie Winchester, Charlotte Eddy, Vishakha Tripathi, Virginia Attard, Anna Lehmann. Wessex Clinical Genetics Service, Princess Anne Hospital, Southampton: Diana Eccles, Anneke Lucassen, Gillian Crawford, Donna McBride, Sarah Smalley.

GEMO

Genetic Modifiers of Cancer Risk in BRCA1/2 Mutation Carriers (GEMO) study: National Cancer Genetics Network «UNICANCER Genetic Group», France. The study was supported by the Ligue Nationale Contre le Cancer; the Association "Le cancer du sein, parlons-en!" Award; the Canadian Institutes of Health Research for the "CIHR Team in Familial Risks of Breast Cancer" program and the French National Institute of Cancer (INCa). They wish to thank all the GEMO

collaborating groups for their contribution to this study. GEMO Collaborating Centers are: Coordinating Centres, Unité Mixte de Génétique Constitutionnelle des Cancers Fréquents, Hospices Civils de Lyon - Centre Léon Bérard, & Equipe «Génétique du cancer du sein», Centre de Recherche en Cancérologie de Lyon: Olga Sinilnikova†, Sylvie Mazoyer, Francesca Damiola, Laure Barjhoux, Carole Verny-Pierre, Mélanie Léone, Nadia Boutry-Kryza, Alain Calender, Sophie Giraud; and Service de Génétique Oncologique, Institut Curie, Paris: Dominique Stoppa-Lyonnet, Marion Gauthier-Villars, Bruno Buecher, Claude Houdayer, Etienne Rouleau, Lisa Golmard, Agnès Collet, Virginie Moncoutier, Cédrick Lefol, Muriel Belotti, Antoine de Pauw, Camille Elan, Catherine Nogues, Emmanuelle Fourme, Anne-Marie Birot. Institut Gustave Roussy, Villejuif: Brigitte Bressac-de-Paillerets, Olivier Caron, Marine Guillaud-Bataille. Centre Jean Perrin, Clermont–Ferrand: Yves-Jean Bignon, Nancy Uhrhammer. Centre Léon Bérard, Lyon: Christine Lasset, Valérie Bonadona, Sandrine Handallou. Centre François Baclesse, Caen: Agnès Hardouin, Pascaline Berthet, Dominique Vaur, Laurent Castera. Institut Paoli Calmettes, Marseille: Hagay Sobol, Violaine Bourdon, Tetsuro Noguchi, Audrey Remenieras, François Eisinger. CHU Arnaud-de-Villeneuve, Montpellier: Isabelle Coupier, Pascal Pujol. Centre Oscar Lambret, Lille: Jean-Philippe Peyrat, Joëlle Fournier, Françoise Révillion, Philippe Vennin†, Claude Adenis. Centre Paul Strauss, Strasbourg: Danièle Muller, Jean-Pierre Fricker. Institut Bergonié, Bordeaux: Emmanuelle Barouk-Simonet, Françoise Bonnet, Virginie Bubien, Nicolas Sevenet, Michel Longy. Institut Claudius Regaud, Toulouse: Christine Toulas, Rosine Guimbaud, Laurence Gladieff, Viviane Feillel. CHU Grenoble: Dominique Leroux, Hélène Dreyfus, Christine Rebischung, Magalie Peysselon. CHU Dijon: Fanny Coron, Laurence Faivre. CHU St-Etienne: Fabienne Prieur, Marine Lebrun, Caroline Kientz. Hôtel Dieu Centre Hospitalier, Chambéry: Sandra Fert Ferrer. Centre Antoine Lacassagne, Nice: Marc Frénay. CHU Limoges: Laurence Vénat-Bouvet. CHU Nantes: Capucine Delnatte. CHU Bretonneau, Tours: Isabelle Mortemousque. Groupe Hospitalier Pitié-Salpétrière, Paris: Florence Coulet, Chrystelle Colas, Florent Soubrier, Mathilde Warcoin. CHU Vandoeuvre-les-Nancy : Johanna Sokolowska, Myriam Bronner. CHU Besançon: Marie-Agnès Collonge-Rame, Alexandre Damette. Creighton University, Omaha, USA: Henry T. Lynch, Carrie L. Snyder.

HEBON

The HEBON study is supported by the Dutch Cancer Society grants NKI1998-1854, NKI2004-3088, NKI2007-3756, the Netherlands Organization of Scientific Research grant NWO 91109024, the Pink Ribbon grant 110005 and the BBMRI grant NWO 184.021.007/CP46. HEBON thanks the registration teams of the Comprehensive Cancer Centre Netherlands and Comprehensive Centre South (together the Netherlands Cancer Registry) and PALGA (Dutch Pathology Registry) for part of the data collection. The Hereditary Breast and Ovarian Cancer Research Group Netherlands (HEBON) consists of the following Collaborating Centers: Coordinating center: Netherlands Cancer Institute, Amsterdam, NL: M.A. Rookus, F.B.L. Hogervorst, F.E. van Leeuwen, S. Verhoef, M.K. Schmidt, N.S. Russell, J.L. de Lange, R. Wijnands; Erasmus Medical Center, Rotterdam, NL: J.M. Collée, A.M.W. van den Ouweland, M.J. Hooning, C. Seynaeve, C.H.M. van Deurzen, I.M. Obdeijn; Leiden University Medical Center, NL: C.J. van Asperen, J.T. Wijnen, R.A.E.M. Tollenaar, P. Devilee, T.C.T.E.F. van Cronenburg; Radboud University Nijmegen Medical Center, NL: C.M. Kets, A.R. Mensenkamp; University Medical Center Utrecht, NL: M.G.E.M. Ausems, R.B. van der Luijt, C.C. van der Pol; Amsterdam Medical Center, NL: C.M. Aalfs, T.A.M. van Os; VU University Medical Center, Amsterdam, NL: J.J.P. Gille, Q. Waisfisz, H.E.J. Meijers-Heijboer; University Hospital Maastricht, NL: E.B. Gómez-Garcia, M.J. Blok; University Medical Center Groningen, NL: J.C. Oosterwijk, A.H. van der Hout, M.J. Mourits, G.H. de Bock; The Netherlands Foundation for the detection of hereditary tumours, Leiden, NL: H.F. Vasen; The Netherlands Comprehensive

Cancer Organization (IKNL): S. Siesling, J.Verloop; The Dutch Pathology Registry (PALGA): L.I.H. Overbeek. The HEBON study is supported by the Dutch Cancer Society grants NKI1998-1854, NKI2004-3088, NKI2007-3756, the Netherlands Organization of Scientific Research grant NWO 91109024, the Pink Ribbon grants 110005 and 2014-187.WO76, the BBMRI grant NWO 184.021.007/CP46 and the Transcan grant JTC 2012 Cancer 12-054. HEBON thanks the registration teams of IKNL and PALGA for part of the data collection.

kCONFAB

kConFab is supported by a grant from the National Breast Cancer Foundation, and previously by the National Health and Medical Research Council (NHMRC), the Queensland Cancer Fund, the Cancer Councils of New South Wales, Victoria, Tasmania and South Australia, and the Cancer Foundation of Western Australia. kConFab wishes to thank Heather Thorne, Eveline Niedermayr, all the kConFab research nurses and staff, the heads and staff of the Family Cancer Clinics, and the Clinical Follow Up Study (which has received funding from the NHMRC, the National Breast Cancer Foundation, Cancer Australia, and the National Institute of Health (USA)) for their contributions to this resource, and the many families who contribute to kConFab. kConFab consists of Amanda B. Spurdle, Helene Holland, Jonathan Beesley, Kelly-Anne Phillips, and Xiaoqing Chen.
